# Supplementary material for: A living biobank of patient-derived ductal carcinoma in situ mouse-intraductal xenografts identifies risk factors for invasive progression
Source: Cancer Cell. 2023 May 8;41(5):986–1002.e9. doi: 10.1016/j.ccell.2023.04.002 (PMC10171335; doi:10.1016/j.ccell.2023.04.002)
Supplement: Document S1. Figures S1–S7 [file mmc1.pdf]

Cancer Cell, Volume *41*

Supplemental information

A living biobank of patient-derived ductal carcinoma *in situ* mouse-intraductal xenografts identifies risk factors for invasive progression

Stefan J. Hutten, Roebi de Bruijn, Catrin Lutz, Madelon Badoux, Timo Eijkman, Xue Chao, Marta Ciwinska, Michael Sheinman, Hendrik Messal, Andrea Herencia-Ropero, Petra Kristel, Lennart Mulder, Rens van der Waal, Joyce Sanders, Mathilde M. Almekinders, Alba Llop-Guevara, Helen R. Davies, Matthijs J. van Haren, Nathaniel I. Martin, Fariba Behbod, Serena Nik-Zainal, Violeta Serra, Jacco van Rheenen, Esther H. Lips, Lodewyk F.A. Wessels, Grand Challenge PRECISION Consortium, Jelle Wesseling, Colinda L.G.J. Scheele, and Jos Jonkers

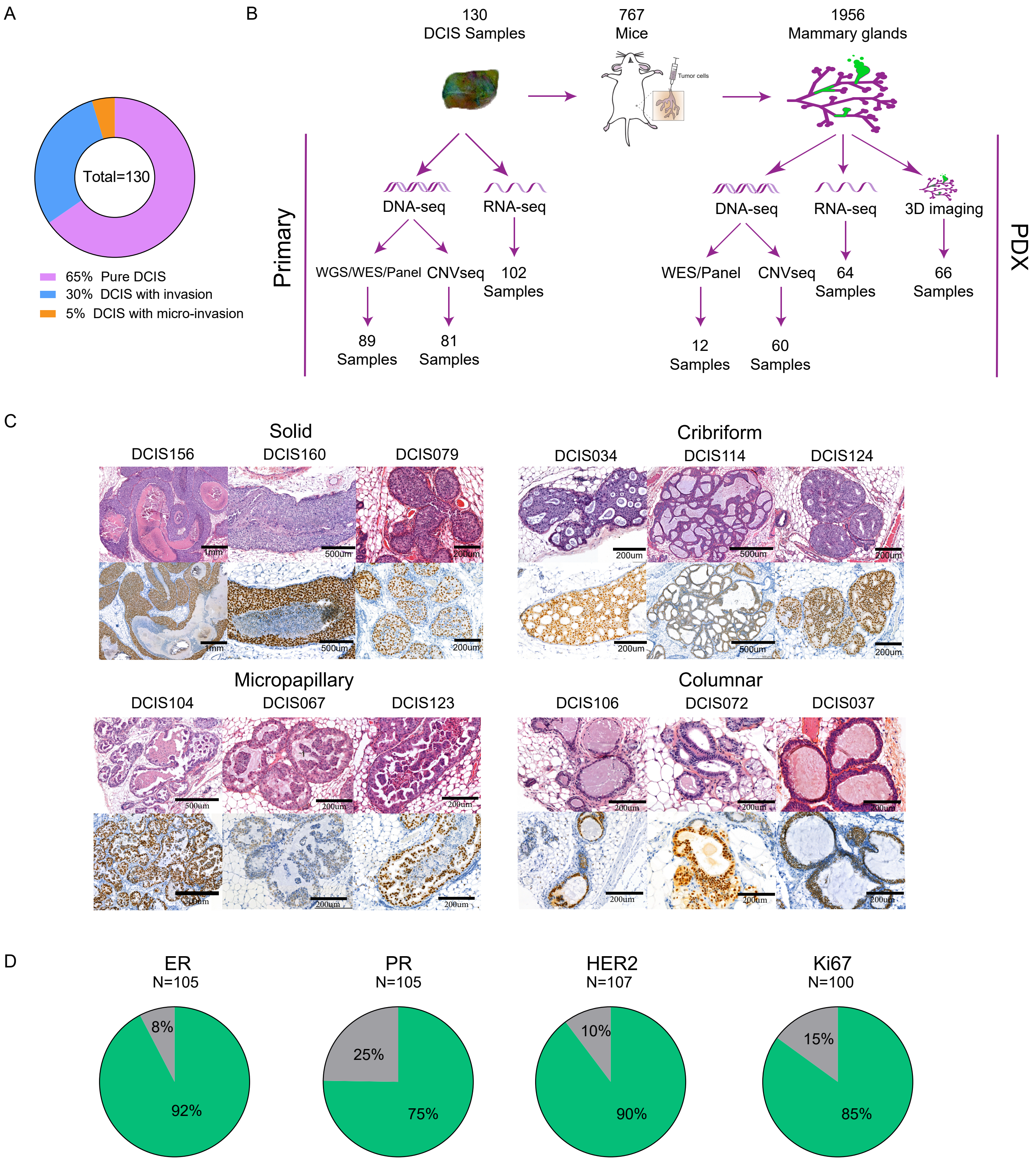
A
Total=130
65% Pure DCIS
30% DCIS with invasion
5% DCIS with micro-invasion
B
130
DCIS Samples
767
Mice
Tumor cells
1956
Mammary glands
Primary
DNA-seq
RNA-seq
WGS/WES/Panel
CNVseq
102
Samples
89
Samples
81
Samples
DNA-seq
RNA-seq
3D imaging
WES/Panel
CNVseq
64
Samples
66
Samples
12
Samples
60
Samples
PDX
C
Solid
DCIS156
DCIS160
DCIS079
Cribriform
DCIS034
DCIS114
DCIS124
Micropapillary
DCIS104
DCIS067
DCIS123
Columnar
DCIS106
DCIS072
DCIS037
D
ER
N=105
8%
92%
PR
N=105
25%
75%
HER2
N=107
10%
90%
Ki67
N=100
15%
85%

Figure S1. A comprehensive collection of 130 DCIS-MIND models, related to figure 1.

A) Pie chart of origin of DCIS with pure DCIS, DCIS with adjacent IBC or DCIS with micro-invasion. **B)** Schematic overview of the DCIS-MIND model collection and analyses performed on DCIS-MIND samples and primary DCIS lesions. **C)** Additional representative H&E (top) and human-specific Ku80 (bottom) images of DCIS-MIND models with a solid, cribriform, (micro-)papillary or columnar growth pattern. **D)** Pie charts of concordance between the primary DCIS lesion and matched DCIS-MIND lesion for ER, PR, HER2 and Ki67 respectively (green is concordant, grey is discordant). ER, PR and HER2 is concordant when both cases are positive (≥10% cells positive) or negative (≤10% cells positive), Ki67 is concordant when the percentage of positive cells is within 10% difference.

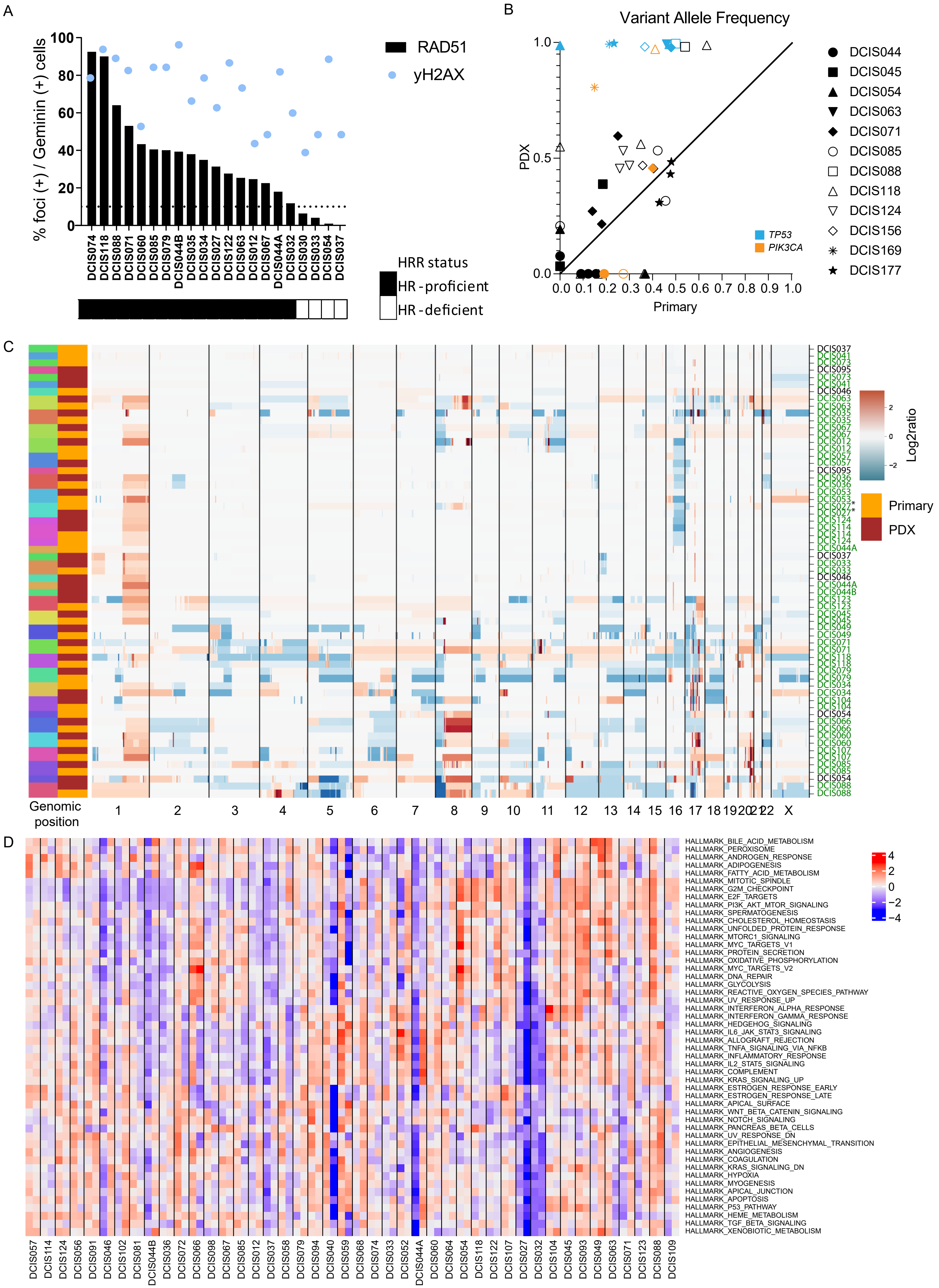
A
% foci (+) / Geminin (+) cells
0
20
40
60
80
100
RAD51
yH2AX
DCIS074
DCIS118
DCIS088
DCIS071
DCIS060
DCIS085
DCIS079
DCIS044B
DCIS035
DCIS034
DCIS027
DCIS122
DCIS063
DCIS012
DCIS067
DCIS044A
DCIS032
DCIS030
DCIS033
DCIS054
DCIS037
HRR status
HR-proficient
HR-deficient
B
Variant Allele Frequency
PDX
Primary
0.0
0.5
1.0
0.0 0.1 0.2 0.3 0.4 0.5 0.6 0.7 0.8 0.9 1.0
TP53
PIK3CA
DCIS044
DCIS045
DCIS054
DCIS063
DCIS071
DCIS085
DCIS088
DCIS118
DCIS124
DCIS156
DCIS169
DCIS177
C
Genomic position
1 2 3 4 5 6 7 8 9 10 11 12 13 14 15 16 17 18 19 20 21 22 X
Log2ratio
2
0
-2
Primary
PDX
D
HALLMARK_BILE_ACID_METABOLISM
HALLMARK_PEROXISOME
HALLMARK_ANDROGEN_RESPONSE
HALLMARK_ADIPOGENESIS
HALLMARK_FATTY_ACID_METABOLISM
HALLMARK_MITOTIC_SPINDLE
HALLMARK_G2M_CHECKPOINT
HALLMARK_E2F_TARGETS
HALLMARK_PI3K_AKT_MTOR_SIGNALING
HALLMARK_SPERMATOGENESIS
HALLMARK_CHOLESTEROL_HOMEOSTASIS
HALLMARK_UNFOLDED_PROTEIN_RESPONSE
HALLMARK_MTORC1_SIGNALING
HALLMARK_MYC_TARGETS_V1
HALLMARK_PROTEIN_SECRETION
HALLMARK_OXIDATIVE_PHOSPHORYLATION
HALLMARK_MYC_TARGETS_V2
HALLMARK_DNA_REPAIR
HALLMARK_GLYCOLYSIS
HALLMARK_REACTIVE_OXYGEN_SPECIES_PATHWAY
HALLMARK_UV_RESPONSE_UP
HALLMARK_INTERFERON_ALPHA_RESPONSE
HALLMARK_INTERFERON_GAMMA_RESPONSE
HALLMARK_HEDGEHOG_SIGNALING
HALLMARK_IL6_JAK_STAT3_SIGNALING
HALLMARK_ALLOGRAFT_REJECTION
HALLMARK_TNFA_SIGNALING_VIA_NFKB
HALLMARK_INFLAMMATORY_RESPONSE
HALLMARK_IL2_STAT5_SIGNALING
HALLMARK_COMPLEMENT
HALLMARK_KRAS_SIGNALING_UP
HALLMARK_ESTROGEN_RESPONSE_EARLY
HALLMARK_ESTROGEN_RESPONSE_LATE
HALLMARK_APICAL_SURFACE
HALLMARK_WNT_BETA_CATENIN_SIGNALING
HALLMARK_NOTCH_SIGNALING
HALLMARK_PANCREAS_BETA_CELLS
HALLMARK_UV_RESPONSE_DN
HALLMARK_EPITHELIAL_MESENCHYMAL_TRANSITION
HALLMARK_ANGIOGENESIS
HALLMARK_COAGULATION
HALLMARK_KRAS_SIGNALING_DN
HALLMARK_HYPOXIA
HALLMARK_MYOGENESIS
HALLMARK_APICAL_JUNCTION
HALLMARK_APOPTOSIS
HALLMARK_P53_PATHWAY
HALLMARK_HEME_METABOLISM
HALLMARK_TGF_BETA_SIGNALING
HALLMARK_XENOBIOTIC_METABOLISM
4
2
0
-2
-4
DCIS057
DCIS114
DCIS124
DCIS056
DCIS091
DCIS046
DCIS102
DCIS081
DCIS044B
DCIS036
DCIS072
DCIS066
DCIS098
DCIS067
DCIS085
DCIS012
DCIS037
DCIS058
DCIS079
DCIS094
DCIS040
DCIS059
DCIS068
DCIS074
DCIS033
DCIS052
DCIS044A
DCIS060
DCIS064
DCIS054
DCIS118
DCIS122
DCIS107
DCIS027
DCIS032
DCIS104
DCIS045
DCIS093
DCIS049
DCIS063
DCIS071
DCIS123
DCIS088
DCIS109

Figure S2. RAD51 assay and RNA expression profile correlation, related to figure 2

A) RAD51 score of a subset of DCIS-MIND models. Models are homologous recombination proficient (HR- proficient), when ≥10% of cells have ≥5 RAD51 foci. **B)** Variant allele frequency (VAF) analyses comparing matched primary DCIS and DCIS-MIND samples, TP53 mutations are indicated in blue and PIK3CA mutations in orange. **C)** Unsupervised clustering of primary DCIS lesions and corresponding DCIS-MIND lesions, based on Copy number aberration (CNA), where blue indicates losses and red indicates gains. Matched primary/MIND models are color-coded and green model annotation indicates a high concordance in CNA. **D)** ssGSEA analyses performed on matched primary DCIS and DCIS-MIND samples and clustered together to show retained expression.

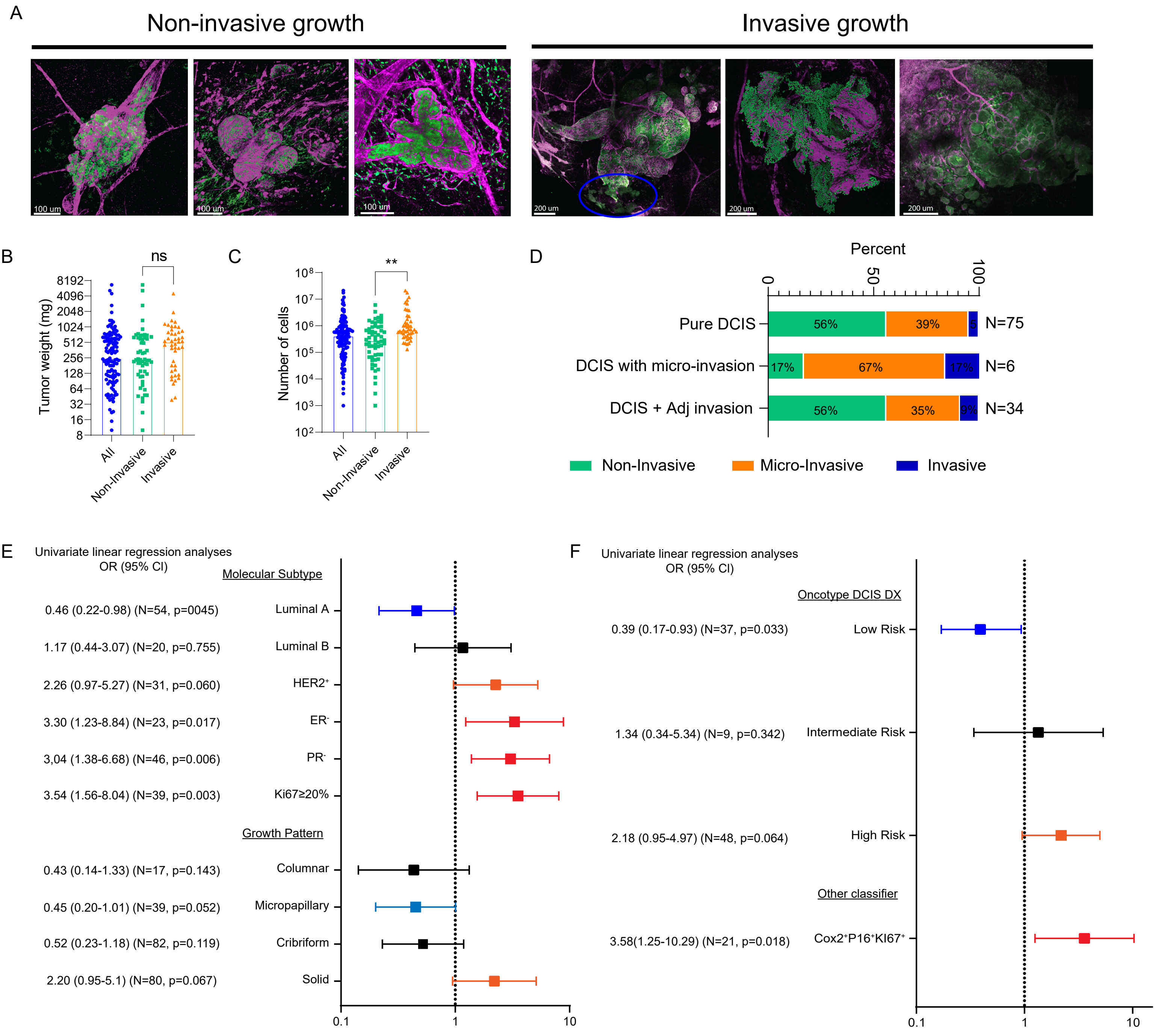
A
Non-invasive growth
Invasive growth
100 um
200 um
B
ns
Tumor weight (mg)
8192
4096
2048
1024
512
256
128
64
32
16
8
All
Non-Invasive
Invasive
C
**
Number of cells
10^8
10^7
10^6
10^5
10^4
10^3
10^2
D
Percent
0
50
100
Pure DCIS
56%
39%
N=75
DCIS with micro-invasion
17%
67%
17%
N=6
DCIS + Adj invasion
56%
35%
9%
N=34
Non-Invasive
Micro-Invasive
Invasive
E
Univariate linear regression analyses
OR (95% CI)
Molecular Subtype
0.46 (0.22-0.98) (N=54, p=0045) Luminal A
1.17 (0.44-3.07) (N=20, p=0.755) Luminal B
2.26 (0.97-5.27) (N=31, p=0.060) HER2+
3.30 (1.23-8.84) (N=23, p=0.017) ER-
3,04 (1.38-6.68) (N=46, p=0.006) PR-
3.54 (1.56-8.04) (N=39, p=0.003) Ki67≥20%
Growth Pattern
0.43 (0.14-1.33) (N=17, p=0.143) Columnar
0.45 (0.20-1.01) (N=39, p=0.052) Micropapillary
0.52 (0.23-1.18) (N=82, p=0.119) Cribriform
2.20 (0.95-5.1) (N=80, p=0.067) Solid
0.1
1
10
F
Univariate linear regression analyses
OR (95% CI)
Oncotype DCIS DX
0.39 (0.17-0.93) (N=37, p=0.033) Low Risk
1.34 (0.34-5.34) (N=9, p=0.342) Intermediate Risk
2.18 (0.95-4.97) (N=48, p=0.064) High Risk
Other classifier
3.58(1.25-10.29) (N=21, p=0.018) Cox2+P16+KI67+
0.1
1
10

Figure S3. Whole-mount images of non-invasive vs invasive growth and additional parameters related to invasive progression, related to figure 3

A) Additional whole-mount images of non-invasive growth (left) and invasive growth (right). Cells of human origin are marked with Ku80 (green), myoepithelial cells are marked with αSMA (magenta). Blue circle indicates invasive cells. **B)** Boxplot of tumor weight of DCIS samples received, divided by all, non-invasive and invasive DCIS-MIND models. Data are represented as mean ± SEM. Unpaired two-tailed Student's t test. ns, not significant. **C)** Bar plot of number of cells obtained after processing of DCIS samples received, divided by all, non-invasive and invasive DCIS-MIND models. Data are represented as mean ± SEM. Unpaired two-tailed Student's t test. $**p < 0.01$. **D**) Diagram showing the distribution of non-invasive, micro-invasive and invasive progression of DCIS-MIND models, comparing models originating from pure DCIS, DCIS with micro-invasion and DCIS with adjacent invasion **E)** Odds ratio table showing univariate risk scores for association between molecular subtype and growth pattern parameters and invasive progression of DCIS in MIND-PDX models based on patient characteristics. **F)** Odds ratio table showing univariate risk scores for the Oncotype DX DCIS- and $COX2^+P16^+Ki67^+$ classifier based on primary data.

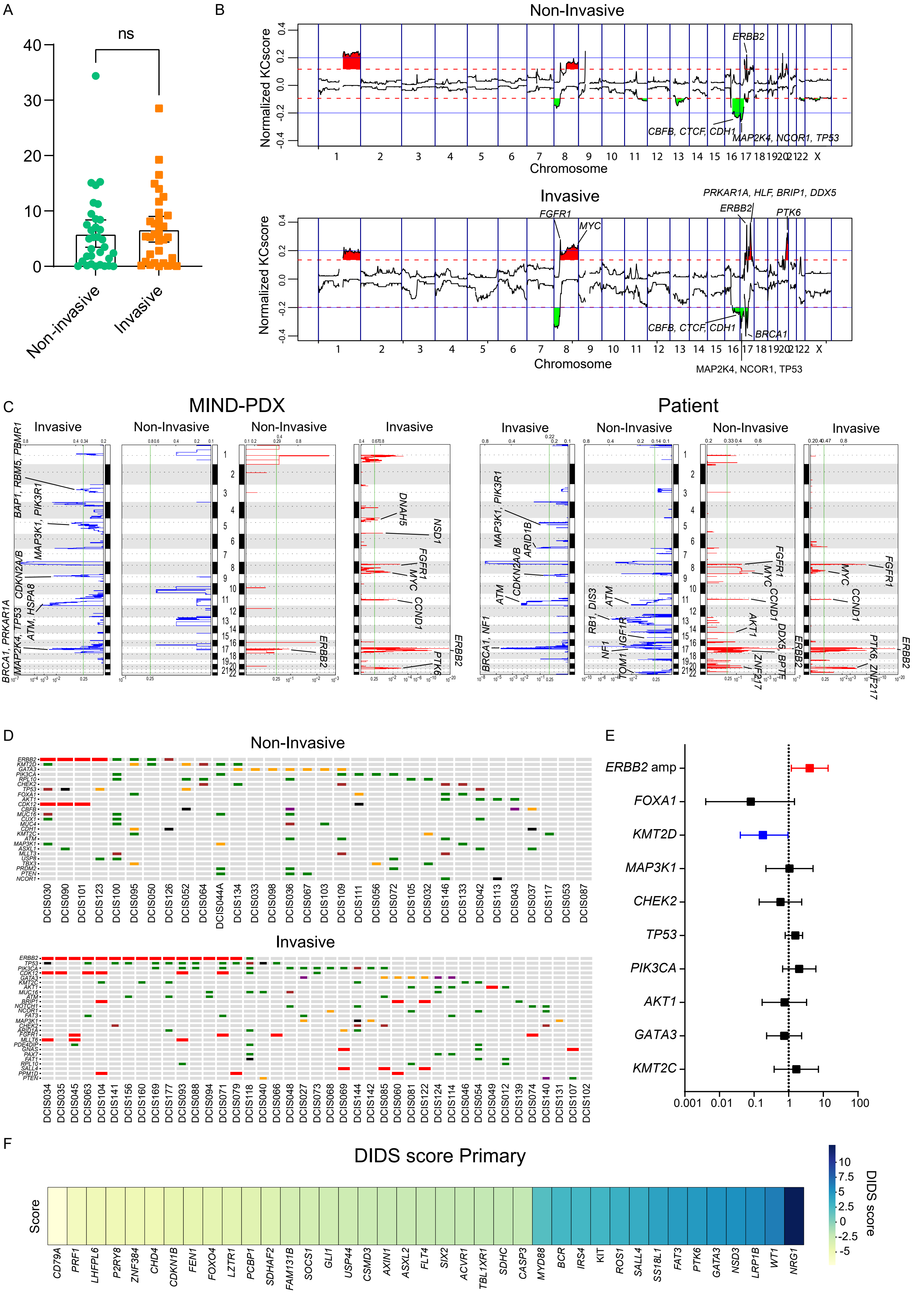
A
ns
40
30
20
10
0
Non-invasive
Invasive
B
Non-Invasive
Normalized KCscore
ERBB2
CBFB, CTCF, CDH1
MAP2K4, NCOR1, TP53
1 2 3 4 5 6 7 8 9 10 11 12 13 14 15 16 17 18 19 20 21 22 X
Chromosome
Invasive
PRKAR1A, HLF, BRIP1, DDX5
ERBB2
PTK6
FGFR1
MYC
BRCA1
MAP2K4, NCOR1, TP53
C
MIND-PDX
Patient
Invasive
Non-Invasive
Non-Invasive
Invasive
Invasive
Non-Invasive
Non-Invasive
Invasive
BAP1, RBM5, PBMR1
MAP3K1, PIK3R1
CDKN2A/B
ATM, HSPA8
BRCA1, PRKAR1A
MAP2K4, TP53
ERBB2
DNAH5
NSD1
FGFR1
MYC
CCND1
PTK6
MAP3K1, PIK3R1
ARID1B
CDKN2A/B
ATM
BRCA1, NF1
RB1, DIS3
NF1
IGF1R
TOM1
AKT1
DDX5
BPTF
ZNF217
D
Non-Invasive
Invasive
E
ERBB2 amp
FOXA1
KMT2D
MAP3K1
CHEK2
TP53
PIK3CA
AKT1
GATA3
KMT2C
0.001 0.01 0.1 1 10 100
F
DIDS score Primary
Score
CD79A PRF1 LHFPL6 P2RY8 ZNF384 CHD4 CDKN1B FEN1 FOXO4 LZTR1 PCBP1 SDHAF2 FAM131B SOCS1 GLI1 USP44 CSMD3 AXIN1 ASXL2 FLT4 SIX2 ACVR1 TBL1XR1 SDHC CASP3 MYD88 BCR IRS4 KIT ROS1 SALL4 SS18L1 FAT3 PTK6 GATA3 NSD3 LRP1B WT1 NRG1
DIDS score
10
7.5
5
2.5
0
-2.5
-5

Figure S4. Comprehensive molecular analyses of primary DCIS for low-risk DCIS vs high-risk DCIS and GISTIC2, related to figure 4

A) Scatter plot of percentage of genome with copy number alterations in matched primary DCIS lesions of DCIS-MIND models. Data are represented as mean ± SEM. Unpaired two-tailed Student's t test. ns, not significant. **B)** KCSmart analysis of CNV-seq data of primary DCIS from non-invasive DCIS models (top panel) and invasive DCIS models (lower panel), showing recurrent DNA copy number losses and gains. Non-invasive DCIS models show recurrent 1q gains and 16q losses, whereas invasive DCIS models show recurrent amplifications of *FGFR1*, *MYC*, *CCND1*, *ERBB2* and *PTK6* as well as losses of *MAP3K1* and *CDKN2A/B*. Genes from our breast cancer gene panel were annotated. **C)** GISTIC2 analysis of CNV-seq data of DCIS-MIND models (left panels) and primary DCIS lesions (right panels) from non-invasive DCIS models and invasive DCIS models. Blue: deletion; Red: Gain, Genes from our breast cancer gene panel were annotated. **D)** Oncoprint showing the mutational landscape of the primary DCIS lesions corresponding to the non-invasive (Top) and invasive (Bottom) DCIS-MIND models, respectively, including amplifications, single-nucleotide variants and insertion-deletions (indels) for the top mutated genes. **E)** Odds ratio table showing risk scores for commonly mutated genes, indicating ERBB2 amplifications and KMT2D mutations as significant odds ratio scores. **F)** Detection of Imbalanced Differential Signal (DIDS) scores for differentially expressed genes between primary DCIS lesions from non-invasive and invasive DCIS models.

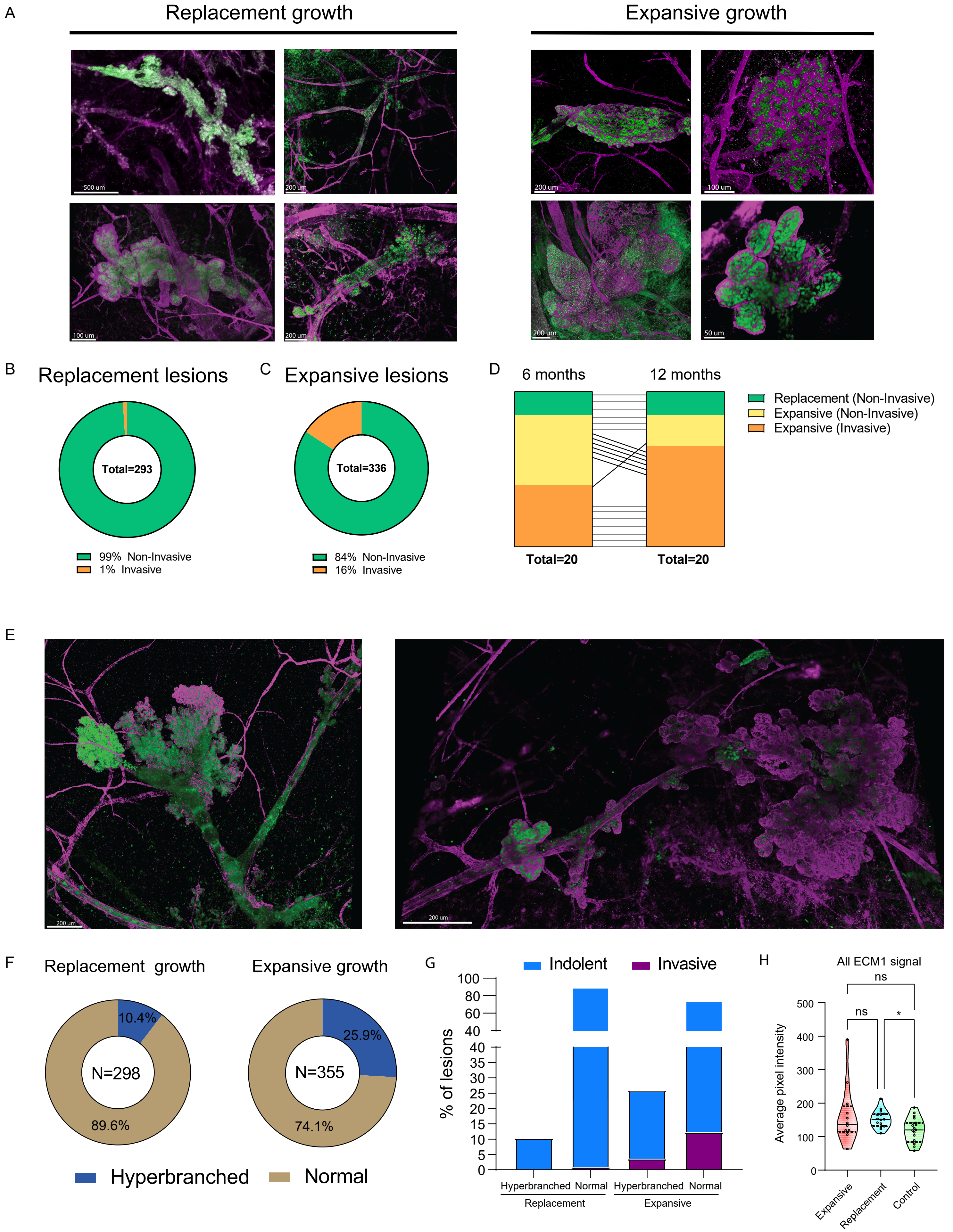

A
Replacement growth
Expansive growth
500 um
200 um
100 um
50 um
B
Replacement lesions
Total=293
99% Non-Invasive
1% Invasive
C
Expansive lesions
Total=336
84% Non-Invasive
16% Invasive
D
6 months
12 months
Total=20
Total=20
Replacement (Non-Invasive)
Expansive (Non-Invasive)
Expansive (Invasive)
E
F
Replacement growth
Expansive growth
10.4%
N=298
89.6%
25.9%
N=355
74.1%
Hyperbranched
Normal
G
Indolent
Invasive
% of lesions
Hyperbranched Normal
Replacement
Hyperbranched Normal
Expansive
H
All ECM1 signal
ns
ns
*
Average pixel intensity
Expansive
Replacement
Control

Figure S5. 3D growth pattern analyses and hyperbranching, related to figure 5

A) Examples of 3D whole-mount imaging of DCIS-MIND lesions, showing replacement growth (left panel) and expansive growth (right panel). Cells of human origin are marked with Ku80 (green), myoepithelial cells are marked with αSMA (magenta). **B)** Percentage of DCIS lesions showing invasion with replacement growth. **C)** Percentage of DCIS lesions showing invasion with expansive growth. **D)** Comparison of 3D growth pattern and invasion at an early- and late time point, 6 months and 12 months respectively. Grey line indicates concordance between early- and late time point, whereas a black line indicates a discordance between early-and late time point. **E)** Representative confocal whole-mount images of hyperbranching occurring around DCIS lesions. Cells of human origin are marked with Ku80 (green), myoepithelial cells are marked with αSMA (magenta) **F)** Percentage of replacement (left) and expansive (right) lesions with hyperbranching. **G)** Bar chart of percentage of DCIS lesions with invasive progression per 3D growth pattern, with or without hyperbranching. **H)** Quantification of total ECM1 expression in DCIS lesions for expansive or replacement growth, and normal mammary ducts. Lines indicate Q^1, Median and Q^3. Two-tailed Mann-Whitney test. ∗ $p < 0.05$, ns, not significant.

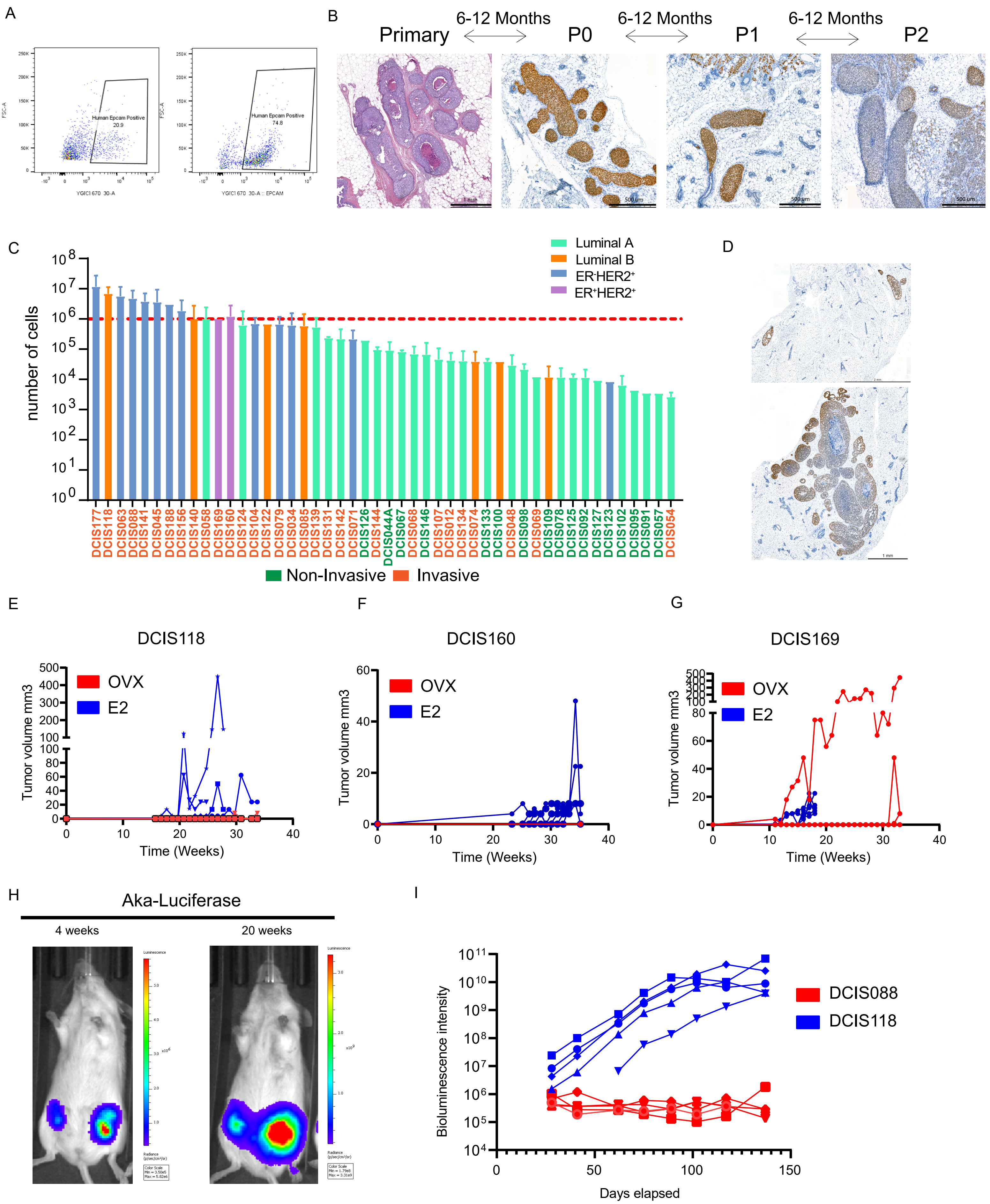
A
Human Epcam Positive
20.9
Human Epcam Positive
74.8
FSC-A
B
Primary
6-12 Months
P0
6-12 Months
P1
6-12 Months
P2
1 mm
500 um
C
Luminal A
Luminal B
ER-HER2+
ER+HER2+
number of cells
10^8
10^7
10^6
10^5
10^4
10^3
10^2
10^1
10^0
DCIS177
DCIS118
DCIS063
DCIS088
DCIS141
DCIS045
DCIS188
DCIS156
DCIS140
DCIS058
DCIS169
DCIS160
DCIS124
DCIS104
DCIS122
DCIS079
DCIS034
DCIS085
DCIS139
DCIS131
DCIS142
DCIS071
DCIS126
DCIS144
DCIS044A
DCIS067
DCIS068
DCIS146
DCIS107
DCIS012
DCIS134
DCIS074
DCIS133
DCIS100
DCIS048
DCIS098
DCIS069
DCIS109
DCIS078
DCIS125
DCIS092
DCIS127
DCIS123
DCIS102
DCIS095
DCIS091
DCIS057
DCIS054
Non-Invasive
Invasive
D
1 mm
E
DCIS118
OVX
E2
Tumor volume mm3
Time (Weeks)
F
DCIS160
OVX
E2
Tumor volume mm3
Time (Weeks)
G
DCIS169
OVX
E2
Tumor volume mm3
Time (Weeks)
H
Aka-Luciferase
4 weeks
20 weeks
I
Bioluminescence intensity
Days elapsed
DCIS088
DCIS118

Figure S6. Sequential transplantation of DCIS and applications, related to figure S6

A) Representative FACS plot of human epithelial cells in a passaged sample marked with EpCAM, before (left) and after (right) magnetic bead sorting. **B)** Representative images of the growth pattern of DCIS118 as primary lesion (H&E), P0 (Ku80), P1 (Ku80) and P2 (Ku80). **C)** Distribution of the average number of tumor cells harvested from DCIS passages. Nineteen transplantable DCIS models yield more than 1,000,000 cells per passage and are therefore suitable for distribution to other labs. Data are represented as mean ± SEM. **D)** Representative images of DCIS085 outgrowth (Ku80) in ovariectomized (OVX) mice (top) and in E2 supplemented mice (bottom) **E-G)** Growth curves of DCIS118 **(E)**, DCIS160 **(F)** and DCIS169 **(G)** outgrowth in OVX mice and in E2-supplemented mice (E2 supplemented mice of DCIS169 had to be sacrificed at 18 weeks due to an infection). **H)** Representative images of bioluminescence of DCIS118 with Akaluciferase at 4 weeks (left) and 20 weeks (right) after injection. **I)** Bioluminescence intensity curves of DCIS118-Akaluciferase and DCIS088-Akaluciferase.

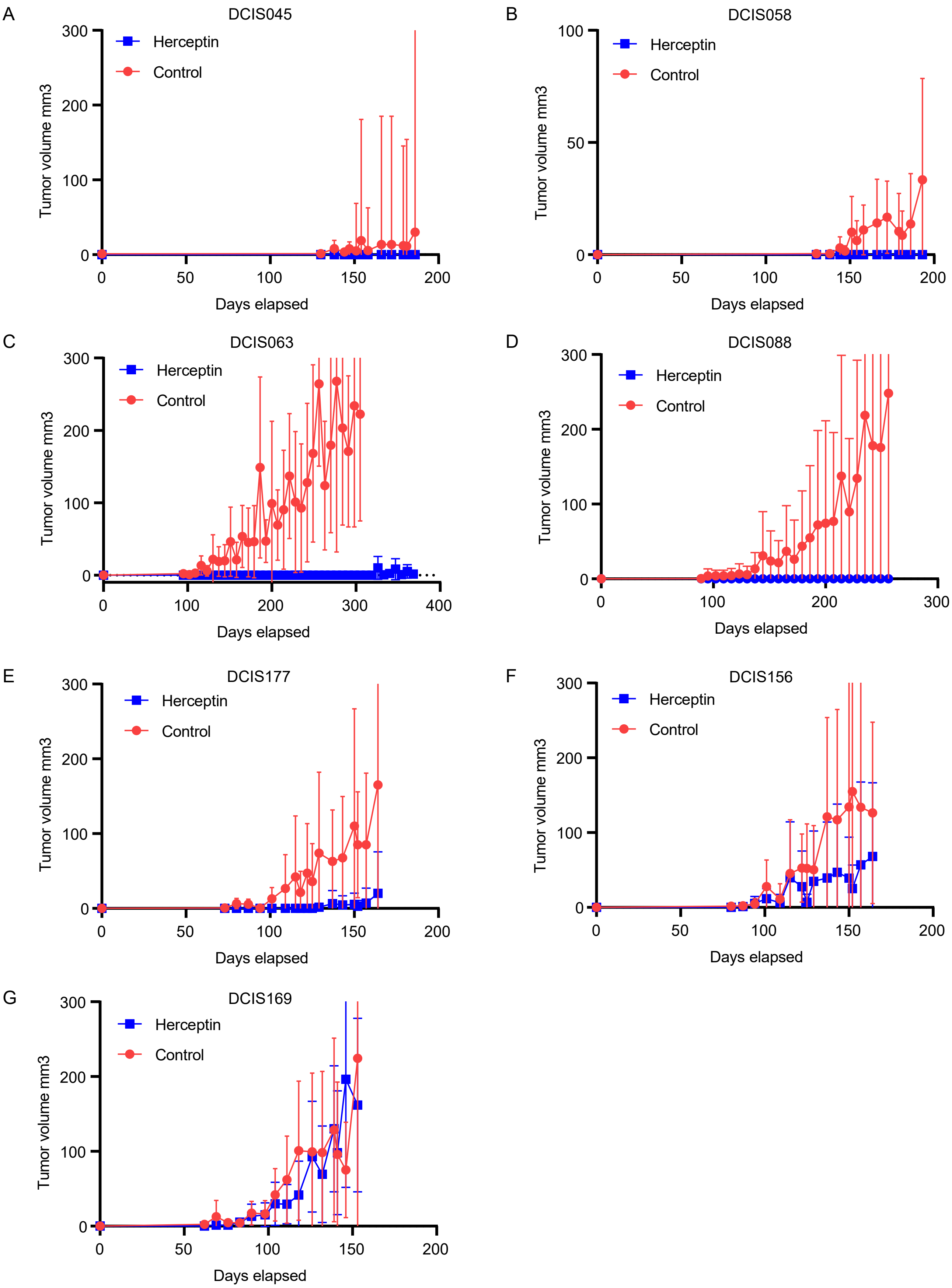

Figure S7. Altering HER2 expression changes growth kinetics of DCIS, related to figure S7

A-G) Growth curves of DCIS045 **(A)**, DCIS058 **(B)**, DCIS063 **(C)**, DCIS088 **(D)**, DCIS177 **(E)**, DCIS156 **(F)**, DCIS169 **(G)** with Herceptin treatment (10 mg/kg) or vehicle.
