# Supplementary material for: A living biobank of patient-derived ductal carcinoma in situ mouse-intraductal xenografts identifies risk factors for invasive progression
Source: Cancer Cell. 2023 May 8;41(5):986–1002.e9. doi: 10.1016/j.ccell.2023.04.002 (PMC10171335; doi:10.1016/j.ccell.2023.04.002)
Supplement: Document S2. Article plus supplemental information [file mmc7.pdf]

Cancer Cell

Article

A living biobank of patient-derived ductal carcinoma *in situ* mouse-intraductal xenografts identifies risk factors for invasive progression

Graphical abstract

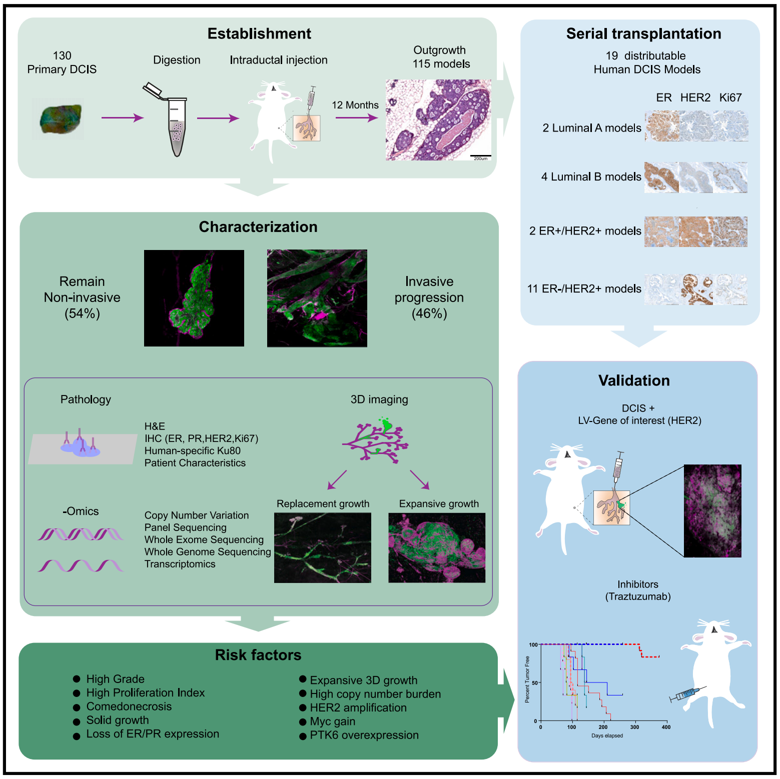

Highlights

- Development of a biobank of DCIS-MIND models retaining primary features
- Identification of multiple factors associated with invasive progression of DCIS
- Identification of two distinct 3D growth patterns associated with outcome
- Development of a collection of 19 distributable DCIS models

Authors

Stefan J. Hutten, Roebi de Bruijn, Catrin Lutz, ..., Jelle Wesseling, Colinda L.G.J. Scheele, Jos Jonkers

Correspondence

j.jonkers@nki.nl

In brief

Hutten et al. generate 115 DCIS-MIND models and characterize them with genomic, transcriptomic, and imaging data. With this biobank they identify risk factors associated with progression of ductal carcinoma *in situ* to invasive breast cancer and create a collection of 19 distributable DCIS models, providing a resource for further research.

Hutten et al., 2023, Cancer Cell *41*, 986–1002
May 8, 2023 © 2023 The Author(s). Published by Elsevier Inc.
https://doi.org/10.1016/j.ccell.2023.04.002

CellPress

Cancer Cell

Article

A living biobank of patient-derived ductal carcinoma *in situ* mouse-intraductal xenografts identifies risk factors for invasive progression

Stefan J. Hutten,[1,2] Roebi de Bruijn,[1,2,11] Catrin Lutz,[1,2] Madelon Badoux,[1,2] Timo Eijkman,[1,2] Xue Chao,[1,2] Marta Ciwinska,[3] Michael Sheinman,[2,11] Hendrik Messal,[1,2] Andrea Herencia-Ropero,[4,5] Petra Kristel,[1] Lennart Mulder,[1] Rens van der Waal,[6] Joyce Sanders,[1] Mathilde M. Almekinders,[1] Alba Llop-Guevara,[4] Helen R. Davies,[7,8] Matthijs J. van Haren,[9] Nathaniel I. Martin,[9] Fariba Behbod,[10] Serena Nik-Zainal,[7,8] Violeta Serra,[4] Jacco van Rheenen,[1,2] Esther H. Lips,[1] Lodewyk F.A. Wessels,[2,11] Grand Challenge PRECISION Consortium, Jelle Wesseling,[1,12,13,14] Colinda L.G.J. Scheele,[3,14] and Jos Jonkers[1,2,14,15,*]
[1]Division of Molecular Pathology, The Netherlands Cancer Institute, 1066 CX Amsterdam, the Netherlands
[2]Oncode Institute, Amsterdam, the Netherlands
[3]Center for Cancer Biology, VIB, Department of Oncology, KU Leuven, 3000 Leuven, Belgium
[4]Experimental Therapeutics Group, Vall d'Hebron Institute of Oncology, 08035 Barcelona, Spain
[5]Department of Biochemistry and Molecular Biology, Autonomous University of Barcelona, Barcelona, Spain
[6]Core Facility Molecular Pathology & Biobanking, The Netherlands Cancer Institute, 1066 CX Amsterdam, the Netherlands
[7]Academic Department of Medical Genetics, School of Clinical Medicine, University of Cambridge, CB2 0QQ Cambridge, UK
[8]Early Cancer Institute, University of Cambridge, CB2 0XZ Cambridge, UK
[9]Biological Chemistry Group, Institute of Biology Leiden, Leiden University, 2302 BH Leiden, the Netherlands
[10]Department of Pathology and Laboratory Medicine, The University of Kansas Medical Center, Kansas City, KS 66103, USA
[11]Division of Molecular Carcinogenesis, The Netherlands Cancer Institute, 1066 CX Amsterdam, the Netherlands
[12]Division of Diagnostic Oncology, Netherlands Cancer Institute – Antonie van Leeuwenhoek Hospital, 1066 CX Amsterdam, the Netherlands
[13]Department of Pathology, Leiden University Medical Center, 2333 ZA Leiden, the Netherlands
[14]Senior author
[15]Lead contact
*Correspondence: j.jonkers@nki.nl
https://doi.org/10.1016/j.ccell.2023.04.002

SUMMARY

Ductal carcinoma *in situ* (DCIS) is a non-obligate precursor of invasive breast cancer (IBC). Due to a lack of biomarkers able to distinguish high- from low-risk cases, DCIS is treated similar to early IBC even though the minority of untreated cases eventually become invasive. Here, we characterized 115 patient-derived mouse-intraductal (MIND) DCIS models reflecting the full spectrum of DCIS observed in patients. Utilizing the possibility to follow the natural progression of DCIS combined with omics and imaging data, we reveal multiple prognostic factors for high-risk DCIS including high grade, HER2 amplification, expansive 3D growth, and high burden of copy number aberrations. In addition, sequential transplantation of xenografts showed minimal phenotypic and genotypic changes over time, indicating that invasive behavior is an intrinsic phenotype of DCIS and supporting a multiclonal evolution model. Moreover, this study provides a collection of 19 distributable DCIS-MIND models spanning all molecular subtypes.

INTRODUCTION

Each year, over 70,000 women are diagnosed with ductal carcinoma *in situ* (DCIS) in the USA, UK, and the Netherlands alone.[1–3] DCIS is a non-invasive lesion that respects the natural tissue barriers of the breast and is, therefore, not life threatening. However, a subset of DCIS lesions eventually overrules the natural tissue barriers imposed by the healthy breast tissue, leading to invasive breast cancer (IBC), a potentially life-threatening disease. Therefore, all women diagnosed with DCIS undergo breast-conserving surgery followed by radiotherapy or mastectomy, with a subset also receiving endocrine therapy.[4] Currently, it is impossible to predict whether a DCIS lesion will stay indolent or progress into invasive disease. This poses a major clinical challenge in determining which DCIS patients to treat, with the risk of overtreating patients with a DCIS that will never progress into invasive disease. To reduce the negative impact of overtreatment in low-risk DCIS patients, yet assure proper treatment for high-risk DCIS patients, it is crucial to identify which factors determine the invasive progression of DCIS.

Most studies investigating the genetic progression of DCIS to IBC focused on synchronous DCIS-IBC, i.e., DCIS with proven

 © 2023 The Author(s). Published by Elsevier Inc.
This is an open access article under the CC BY license (http://creativecommons.org/licenses/by/4.0/).

CellPress
OPEN ACCESS

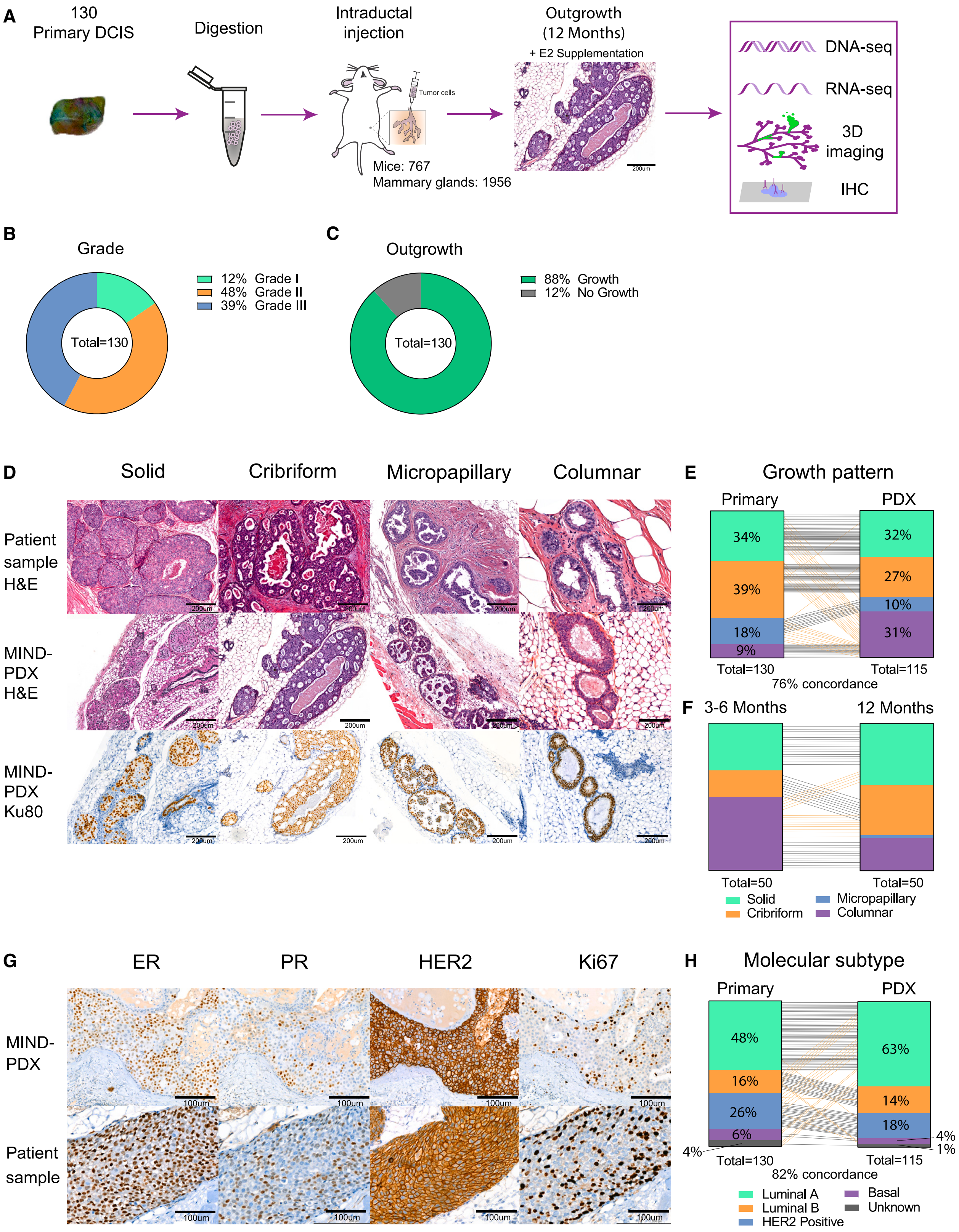

Figure 1. A living biobank of MIND-PDX models of DCIS retaining histological and molecular features of primary lesions

(A) Schematic overview of the generation and characterization of 130 MIND-PDX models of DCIS.

(B) Pie chart of the grade distribution of the primary DCIS lesions.

(legend continued on next page)

potential to progress to IBC, and are therefore not informative for what drives progression of pure DCIS to IBC. To date, these studies were unable to identify a single genomic alteration predictive of progression into invasive disease,[5–7] indicating that progression of DCIS is likely a complex process involving various tumor cell-intrinsic and microenvironmental factors that ultimately determine whether or not a DCIS lesion remains indolent.

Mouse models of DCIS are limited and do not recapitulate hormone dependence and progression from atypical cell growth into DCIS and subsequent IBC.[8] Furthermore, the study of human DCIS tissue from patients is limited by the fact that the natural progression cannot be followed over time within the same patient. As a result, the dynamics and natural course of DCIS progression remain poorly understood. Since human DCIS lesions initiate inside the mammary ducts, a mouse-intraductal (MIND) injection technique was developed to inject human cell lines and patient-derived DCIS samples into the mammary ducts of immunocompromised mice.[9,10] This method results in DCIS lesions retaining the sample-specific estrogen receptor (ER), progesterone receptor (PR), and HER2 expression, and can be used to follow the progression of DCIS lesions over time.[8,11–13]

Here, we utilize the MIND injection technique to generate an extensively characterized living biobank of 115 patient-derived DCIS xenograft models representing the full spectrum of human DCIS. Similar to DCIS patients, a subset of DCIS-MIND models showed progression into IBC over time, enabling us to link the natural progression of DCIS to histopathological and molecular data. Using this approach, we identified HER2, MYC, and PTK6 amplification, high copy number aberration (CNA) burden, solid growth pattern, grade 3, high Ki67 level, and distinct 3D growth pattern as risk factors, whereas a luminal A subtype or columnar growth correlate with low-risk DCIS. Sequential transplantation resulted in a collection of 19 distributable DCIS-MIND models, including 2 luminal A, 4 luminal B, 2 ER^+HER2^+, and 11 ER^-HER2^+ models. Our DCIS-MIND biobank provides a useful resource to study the natural progression of human DCIS and to identify factors associated with invasive progression.

RESULTS

Establishing a living biobank of DCIS-MIND models

To study the natural progression of DCIS, we set out to create a living biobank of patient-derived MIND models of DCIS that reflects the full histopathological spectrum of DCIS, including pure DCIS, DCIS with adjacent IBC, and DCIS with micro-invasion. To this end, we collected fresh DCIS samples from patients who underwent surgery at the Netherlands Cancer Institute. Fresh DCIS tissue was immediately processed into single cells by overnight enzymatic digestion and intraductally injected into immunodeficient *NOD-scid;Il2rg^{null}* (NSG) mice. In total, we obtained 130 DCIS samples, which were intraductally injected into 1,956 mammary glands of 767 NSG mice supplemented with estradiol (E2) (Figure 1A; Table S1). The 130 patient samples included 85 pure DCIS (65%), 6 DCIS with micro-invasion (5%), and 39 DCIS with adjacent IBC (30%) (Figure S1A). Twelve months after intraductal injection, the injected mammary glands were dissected and subsequently analyzed using immunohistochemistry (IHC), whole-gland 3D imaging, and genomic and transcriptomic analysis (Figures 1A and S1B). The collection of samples included grade I (12%), II (48%), and III (39%) DCIS, with an overall take rate of 88% (115 out of 130) (Figures 1B and 1C).

DCIS models retain histological and mutational features of the primary lesions

DCIS can be classified into subgroups based on their histological features, including growth pattern, hormone receptor status, and grade.[14] The collected DCIS-MIND models reflected four types of growth patterns described in patients: solid, cribriform, micropapillary, and columnar (Figures 1D and S1C). The growth patterns of the primary DCIS lesions were retained in 76% (87 out of 115) of the MIND models. In most cases where the growth pattern did not match, the DCIS-MIND lesions showed columnar growth instead of a solid, cribriform, or micropapillary growth pattern observed in the primary DCIS (Figure 1E). Time point analyses revealed that columnar lesions observed at early time points frequently developed into a different growth pattern at later time points, indicating that these DCIS lesions may require a longer time to progress into their final growth pattern (Figure 1F).

We also checked whether the DCIS-MIND models recapitulated the expression of established biomarkers in breast cancer, i.e., ER, PR, HER2, and Ki67. Indeed, we identified a high concordance between the original patient sample and the DCIS-MIND models for the expression of hormone receptors, Ki67, and the surrogate molecular DCIS subtypes: luminal A, luminal B, $HER2^+$, and basal DCIS (Figures 1G and 1H). Expression was similar in 97 out of 105 models (92%) for ER, 79/105 models (75%) for PR, 96/107 models (90%) for HER2, and 85/100 models (85%) for Ki67 (Figure S1D). The discordance observed in certain models might be caused by heterogeneity in the patient sample or progression of the DCIS cells after engraftment.

Comparative genomic analyses of the primary DCIS lesions and the corresponding MIND models using whole-exome sequencing (WES) (n = 60), whole-genome sequencing (WGS) (n = 11), or panel sequencing (n = 18) revealed that our collection

(C) Pie chart of the DCIS take rate in MIND-PDX models.
(D) Examples of H&E-stained sections of the different growth patterns observed in primary DCIS lesions (top row) or DCIS-MIND outgrowths (middle row). Bottom row: human-specific Ku80 staining showing that DCIS-MIND outgrowths have a human origin.
(E) Distribution of growth patterns of primary DCIS lesions and the corresponding DCIS-MIND outgrowths. A black line indicates concordance between primary and PDX, whereas an orange line indicates a discordance between primary and PDX.
(F) Growth pattern analyses between an early (3–6 months) and late (12 months) time points.
(G) Examples of immunohistochemistry for ER, PR, HER2, and Ki67 expression in DCIS-MIND lesions (top row) vs. matched primary DCIS lesions (bottom row).
(H) Distribution of molecular subtypes (luminal A: ER^+, $PR^{+/-}$, $HER2^-$, Ki67 < 20%; luminal B: ER^+, $PR^{+/-}$, $HER2^-$, Ki67 $\geq$ 20%; $HER2^+$: $ER^{+/-}$, $PR^{+/-}$, and $HER2^+$; basal: ER^-, PR^-, $HER2^-$) of primary DCIS lesions and the corresponding DCIS-MIND lesions. A black line indicates concordance between primary and PDX, whereas an orange line indicates a discordance between primary and PDX. See also Figure S1 and Table S1.

CellPress
OPEN ACCESS

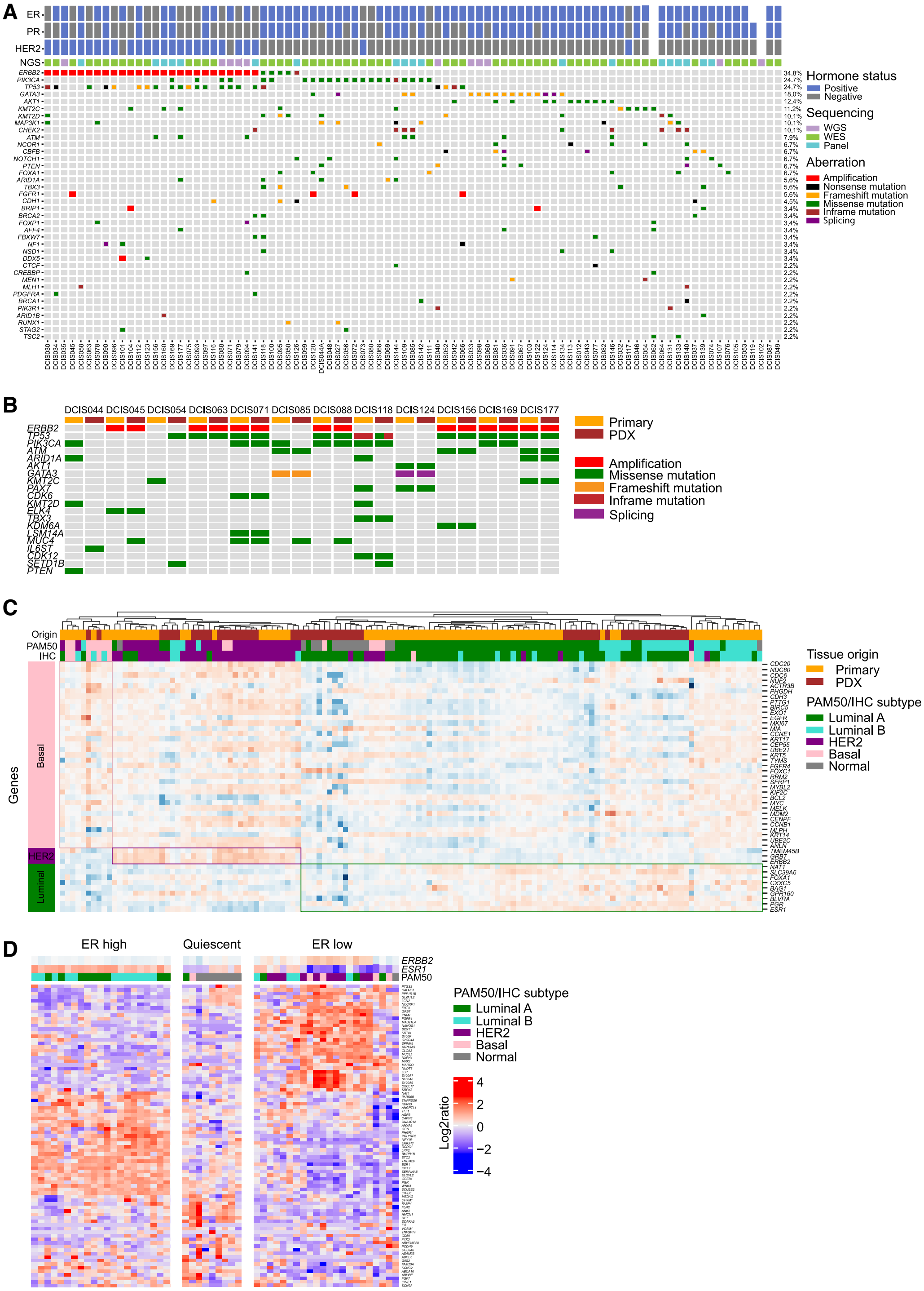

Figure 2. DCIS-MIND models retain mutational and transcriptional features of the primary DCIS lesions

(A) Oncoprint showing the mutational landscape of the primary DCIS lesions, including amplifications, single-nucleotide variants, and insertion-deletions (indels) for the top mutated genes in our breast cancer gene panel. Annotations for each model includes ER, PR, and HER2 status.

(B) Oncoprint showing amplifications, single-nucleotide variants, and insertion-deletions (indels) in cancer genes in primary DCIS lesions and corresponding DCIS-MIND lesions for the top mutated genes.

(C) Unsupervised clustering of DCIS-MIND lesions based on PAM50 genes, showing clustering of luminal, HER2^{+}, and basal-like DCIS lesions. Annotations include origin (primary or PDX) and molecular subtype based on PAM50 or IHC.

(D) Unsupervised clustering of DCIS-MIND lesions based on 90 informative genes resulting in three DCIS subtypes proposed by Strand et al.[15] See also Figure S2 and Tables S2 and S3.

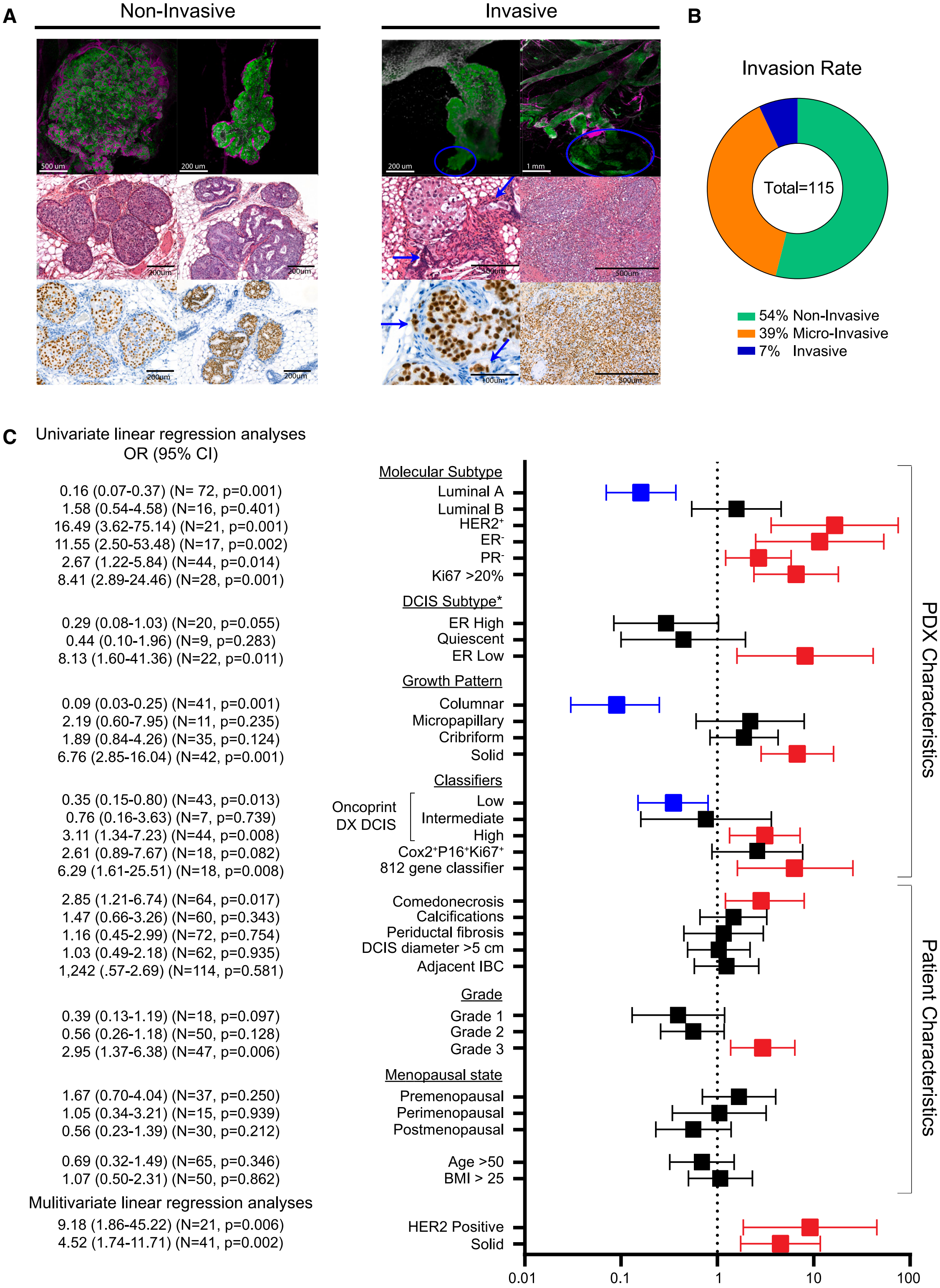

Figure 3. Comparison of clinical biomarkers related to invasive progression of DCIS

(A) Whole-mount analysis (top row), H&E staining (middle row), and human-specific Ku80 staining (bottom row) of DCIS-MIND lesions showing non-invasive growth (left panel) or invasive growth (right panel). Cells of human origin are marked with Ku80 (green), myoepithelial cells are marked with alpha-smooth muscle actin (αSMA) (magenta). Blue arrows/circles indicate invasive cells.

(B) Pie chart of the percentage of DCIS-MIND lesions with non-invasive growth, micro-invasion, or invasive progression.

(legend continued on next page)

of DCIS-MIND models reflects the heterogeneity of human DCIS with respect to mutations in known cancer genes and genomic aberrations (Figure 2; Tables S2 and S3).[16] The most common alterations in our DCIS biobank include *ERBB2* amplifications (34.8%) and mutations in *TP53* (24.7%), *PIK3CA* (24.7%), *GATA3* (18.0%), and *AKT1* (12.4%) (Figure 2A). RAD51 foci analyses on a subset of MIND models (n = 20) showed that DCIS accumulate high levels of dsDNA damage (yH2AX foci) and 20% of DCIS PDX are predicted to be homologous recombination deficient (HRD), comparable with the proportion of IBC with genetic HRD signatures and/or genomic scars (Figure S2A).[17]

To further validate the concordance between primary DCIS lesions and the MIND models we performed DNA CNA analysis for 34 matched pairs of primary and DCIS-MIND samples (Figure S2C), as well as WES or targeted sequencing for 12 pairs (Figure 2B). Both analyses showed high concordance between the primary lesion and MIND model on a global level (Figure 2B). At the local level, small variations were detected, such as absence or addition of a mutation, which could be explained by variant allele frequencies (Figure S2B), or an additional gain or loss (Figure S2C), which might indicate evolution of the DCIS-MIND model or heterogeneity of the primary lesion.

In addition, we performed transcriptomic analyses of matched primary and DCIS-MIND samples. Single sample gene set enrichment analysis (GSEA) showed correlation at the hallmark gene set level (Figure S2D), and analyses of PAM50 genes[18,19] revealed high concordance with the IHC subtype (83%, 111 out of 134) (Figure 2C). Moreover, primary and DCIS-MIND samples clustered together based on the PAM50 signature (Figure 2C). Finally, we performed DCIS-specific subtyping as published by Strand et al.,[15] which showed clear separation of DCIS-MIND samples into ER-high, ER-low, and quiescent subtypes (Figure 2D).

Overall, the high concordance for growth patterns, molecular subtype, mutational landscape, CNA profiles, and gene expression patterns indicates that our DCIS-MIND models faithfully recapitulate the original patient lesions and reflect the heterogeneity observed in the DCIS patient population, and are therefore a representative collection of models to study DCIS progression.

HER2 overexpression, solid growth pattern, high proliferative index, and grade 3 correlate to invasive progression

We utilized our DCIS-MIND biobank to follow the natural progression of DCIS over 12 months and identify which cases represent high-risk DCIS or low-risk DCIS. Progression of DCIS lesions was scored using both 2D H&E analysis and a 3D whole-gland imaging technique, which enabled us to identify all human-derived DCIS lesions using human-specific Ku80 immunolabeling. In both 2D sections and 3D images, we scored the number of progressed samples showing protrusion of DCIS cells through the murine myoepithelial cell layer (identified by alpha-smooth muscle actin labeling). Lesions were classified into non-invasive (no protrusions), micro-invasive (<2 mm protrusion), or invasive (>2 mm protrusion) subtypes (Figures 3A and S3A). Overall, 54% of DCIS samples remained non-invasive, while 39% and 7% of DCIS samples showed micro-invasion or invasion, respectively (Figure 3B).

We next sought to identify differences between the progressed and non-progressed DCIS samples. Weight of the tissue received from the surgical material did not influence the likelihood of DCIS invasive progression, although the total number of collected DCIS cells was somewhat higher in DCIS samples showing invasive progression compared with the non-progressive DCIS samples (Figures S3B and S3C). In addition, no difference in invasive progression was observed between pure DCIS samples and DCIS samples with adjacent IBC (Figures 3C and S3D). Hence, the natural evolution of DCIS in MIND models is not affected by the size of the obtained resection material or the presence of adjacent IBC.

The ability to follow the natural progression of DCIS-MIND lesions provides a unique opportunity to identify clinical biomarkers associated with invasive progression. We therefore performed univariate linear regression analysis to calculate odds ratio (OR) related to invasive progression for clinical parameters, such as molecular subtype, growth pattern, grade, menopausal state, age, and BMI, as well as the new DCIS subtyping. We found significantly higher OR scores for DCIS lesions with a $HER2^+$ molecular subtype (OR = 16.49; 95% CI, 3.62–75.14), ER negativity (OR = 11.55; 95% CI, 2.50–53.48), PR negativity (OR = 2.67; 95% CI, 1.22–5.84), Ki67 $\geq$ 20% (OR = 8.41; 95% CI, 2.89–24.46), ER-low DCIS subtype (OR = 8.31; 95% CI, 1.60–41.36), solid growth (OR = 6.76; 95% CI, 2.85–16.04), comedonecrosis (OR = 2.85; 95% CI, 1.21–6.74), or grade 3 (OR = 2.95; 95% CI, 1.37–6.38). Luminal A (OR = 0.16; 95% CI, 0.07–0.37) DCIS lesions or lesions with columnar growth (OR = 0.09; 95% CI, 0.03–0.37) showed a significantly lower risk of developing IBC. Multivariate linear regression analysis of the clinical parameters identified HER2 positivity (OR = 9.18; 95% CI, 1.86–45.22) and solid growth (OR = 4.52; 95% CI, 1.74–11.71) as independent risk factors (Figures 3C and S3E).

To further test the clinical validity of our dataset, we used the onco-type DX DCIS,[20] $COX2^+P16^+$ Ki67,[21,22] and 812-gene[15] classifiers for risk of DCIS recurrence. On primary DCIS data, the $COX2^+P16^+$ $Ki67^+$ classifier showed a significant OR for DCIS progression, while the onco-type DX DCIS showed a trend for predicting low-, intermediate-, or high-risk DCIS. On the PDX data, both the onco-type DX DCIS and 812-gene classifiers showed significant prediction of DCIS progression, whereas the $COX2^+P16^+$ $Ki67^+$ showed a trend toward a higher risk of invasive progression (Figures 3C and S3F).

Overall CNAs as well as specific aberrations in MYC, ERBB2, and PTK6 correlate to invasive progression

We next used genomics data from the patient samples and DCIS-MIND models to identify additional biomarkers. CNA-seq analysis revealed a significant higher percentage of overall

(C) Odds ratio table showing risk scores (univariate and multivariate linear regression models) for association between common clinical parameters, DCIS subtypes as proposed by Strand et al.,[15] and multiple invasive recurrence classifiers for invasive progression of DCIS-MIND lesions, identifying HER2 overexpression and solid growth patterns as independent risk factors. Molecular subtype and growth pattern parameters are based on DCIS-MIND characteristics while the other parameters are based on patient characteristics. See also Figure S3.

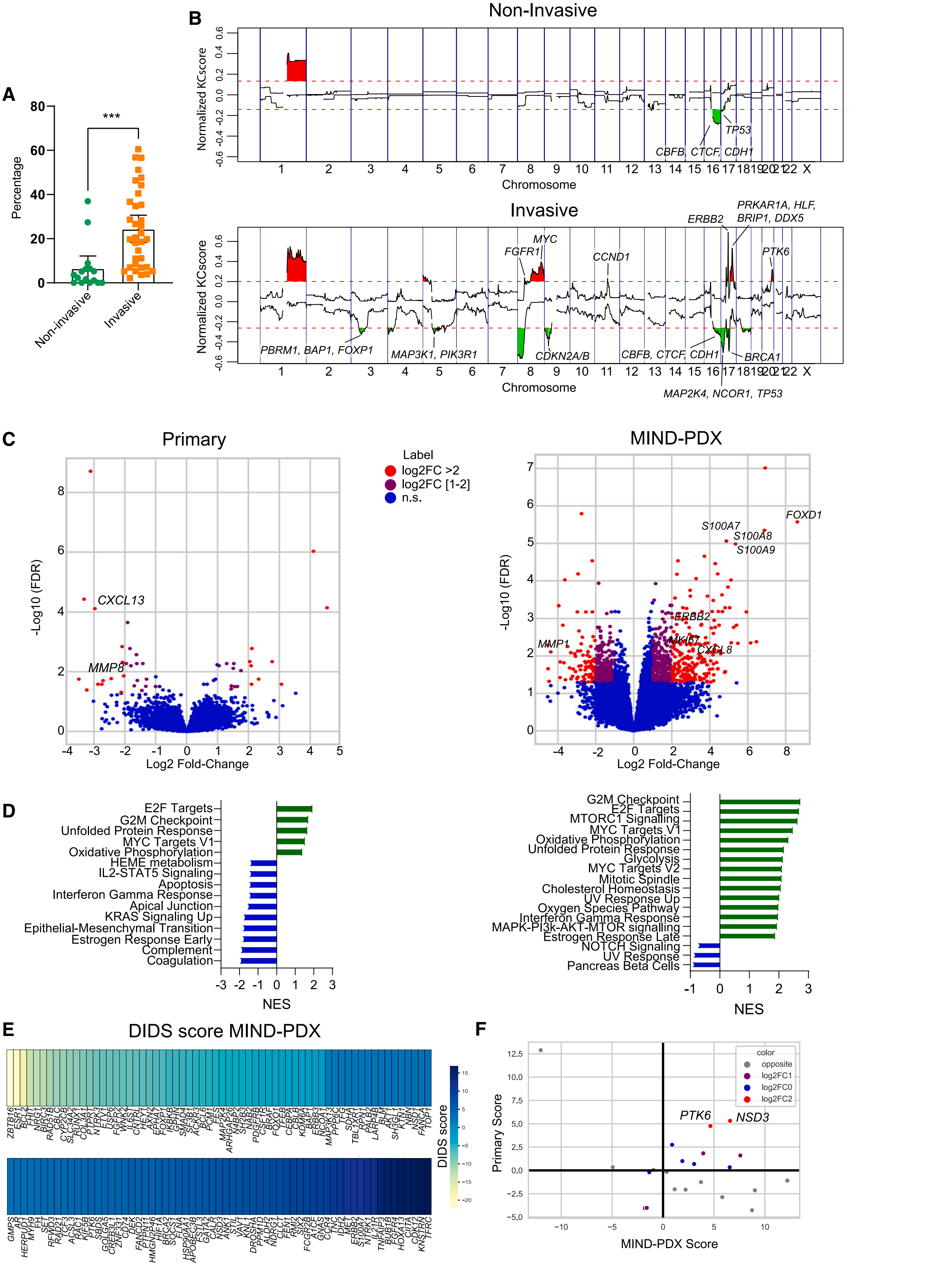

Figure 4. Comprehensive molecular analysis of low-risk vs. high-risk DCIS

(A) Scatterplot of percentage of genome with copy number alterations, showing significantly higher numbers of alterations in DCIS-MIND lesions with invasive progression. Data are represented as mean ± SEM. Unpaired two-tailed Student's t test. ***$p < 0.001$.

(legend continued on next page)

CNAs in DCIS samples with invasive progression compared with non-invasive DCIS samples in the MIND models (Figure 4A), whereas this trend was not visible in the patient samples (Figure S4A). Notably, the overall lower CNA percentage in the primary DCIS samples could be caused by quenching of the CNA signals due to the presence of normal DNA from myoepithelial and stromal cells, resulting in CNAs with smaller amplitude that are more difficult to pick up.

To identify CNAs that are more common in DCIS with invasive progression, we performed both KCSmart and GISTIC2 analyses (Table S4). KCSmart identified 1q gains and 16q losses as common aberrations in indolent DCIS and DCIS with invasion, suggesting that these aberrations represent early events during development of DCIS. In addition, both KCSmart and GISTIC2 identified gains/amplifications of *FGFR1*, *MYC*, *CCND1*, *ERBB2*, and *PTK6*, as well as losses as *CDKN2A/B*, *BRCA1*, and *MAP3K1* to occur more frequently in DCIS-MIND models with invasive progression (Figures 4B and S4C). These findings were confirmed in the CNA-seq analysis of the primary DCIS lesions, with the exception of *CCND1* (Figures S4B and S4C). Mutational analysis did not reveal significant differences between non-invasive DCIS and DCIS with invasive progression except for mutations in *KMT2D*, which were more common in non-invasive DCIS (Figures S4D and S4E).

Furthermore, we examined the transcriptomes of primary DCIS lesions and DCIS-MIND lesions to validate our correlations linked to DCIS progression and identify additional factors (Figure 4C; Table S5). Interestingly, we only found limited significant differences in the gene expression profiles from the matched primary DCIS lesions when comparing progressed with non-progressed DCIS-MIND models. However, analysis of transcriptomic data from the MIND models did reveal more significant differences in gene expression between indolent DCIS and DCIS with invasive progression, suggesting that stromal cells might obscure significant changes in the primary patient samples. Transcriptomic analysis of the MIND models again identified high expression of *ERBB2* and *Ki67* as risk factors, as well as additional factors such as *S100A8/A9* and *FOXD1*, which are described to drive breast cancer proliferation.[23–25] GSEA of primary DCIS lesions and the corresponding PDX lesions identified upregulation of hallmark gene sets associated with proliferation (E2F targets, G2M checkpoint, MYC targets) (Figure 4D, Table S5).

As DCIS is a heterogeneous disease, we did not expect one molecular marker to explain all cases with invasive progression. Therefore, we used detection of imbalanced differential signal (DIDS) scores[26] to identify genes that are differentially expressed in subgroups of the DCIS models. DIDS analysis on DCIS-MIND samples identified previously mentioned genes such as high expression of *ERBB2* in DCIS with invasive progression or high expression of *ESR1* in non-invasive DCIS (Figure 4E; Table S5). DIDS scores that were found in both DCIS-MIND samples and primary samples with a score in the same direction were *NSD3* and *PTK6* (Figures 4F and S4F). Increased copy number gains of *PTK6* were also identified in DCIS models with invasive progression (Figure 4B). PTK6 is a tyrosine kinase, which was previously associated with breast cancer progression and may therefore be a candidate biomarker for high-risk DCIS, as well as a potential therapeutic target.[27,28]

Whole-mount 3D analysis reveals two distinct growth patterns with strong correlation to invasive progression

To assess whether growth characteristics of DCIS are associated with indolent or invasive growth, we analyzed the 3D whole-mount images. Interestingly, based on their 3D morphology, DCIS lesions could be classified into two distinct growth patterns: replacement growth and expansive growth. In DCIS with replacement growth, the human DCIS cells replace the existing mouse luminal epithelium and populate the ducts, but do not severely disturb the architecture of the ducts. DCIS with expansive growth is characterized by tumor cells that do not grow within the ducts, but rather expand perpendicularly to the ducts (Figures 5A and S5A). The expansive lesions showed a higher volume and more spherical morphology when compared with replacement lesions, which had a smaller volume and a more elongated shape (Figures 5B and 5C). Expansive lesions remained more localized within the ductal tree and caused loss of contacts between the myoepithelial cells resulting in a discontinuous myoepithelial cell layer (Figures 5A and S5A).

As a result of the potential mechanical pressure on the myoepithelial cells and the basement membrane, we identified two types of morphological aberrations. On the one hand, lesion outgrowth led to bulging and hyperbranching of the ductal epithelium (Figure S5E). Not only did the epithelium containing the lesion show this hyperbranching phenotype, but the healthy neighboring ducts also adopted a hyperbranched morphology (Figure S5E). This suggests that hyperbranching might be induced by a paracrine signal from either the stressed myoepithelial cells or the tumor cells. On the other hand, expansive lesion growth resulted in breakage of the myoepithelial cell layer and the basement membrane and subsequent invasive growth (Figures 3A, 5A, S3A, and S5B).

Importantly, invasive events almost exclusively occurred in DCIS models with expansive growth, whereas the vast majority of the models with replacement growth stayed indolent (Figures 5D, S5B, and S5C). Analysis of early and late time points (6 vs. 12 months after injection) showed that lesions with replacement growth remained indolent, while most expansively growing lesions that were indolent at the early time point progressed to invasive growth at the late time point (Figure S5D).

(B) KCSmart analysis of CNV-seq data from non-invasive DCIS models (top panel) and invasive DCIS models (lower panel), showing recurrent DNA copy number losses and gains. Genes from our breast cancer gene panel were annotated.

(C) Volcano plot showing differentially expressed genes between non-invasive and invasive DCIS models (left plot: primary DCIS lesions; right plot: DCIS-MIND models).

(D) Gene set enrichment analysis for hallmark gene sets (left plot: primary DCIS lesions; right plot: DCIS-MIND models).

(E) Detection of imbalanced differential signal (DIDS) scores for differentially expressed genes between non-invasive and invasive DCIS models.

(F) Scatterplot of DIDS scores from primary DCIS lesions and PDX models, showing *PTK6* and *NSD3* as concordant hits. See also Figure S4 and Tables S4 and S5.

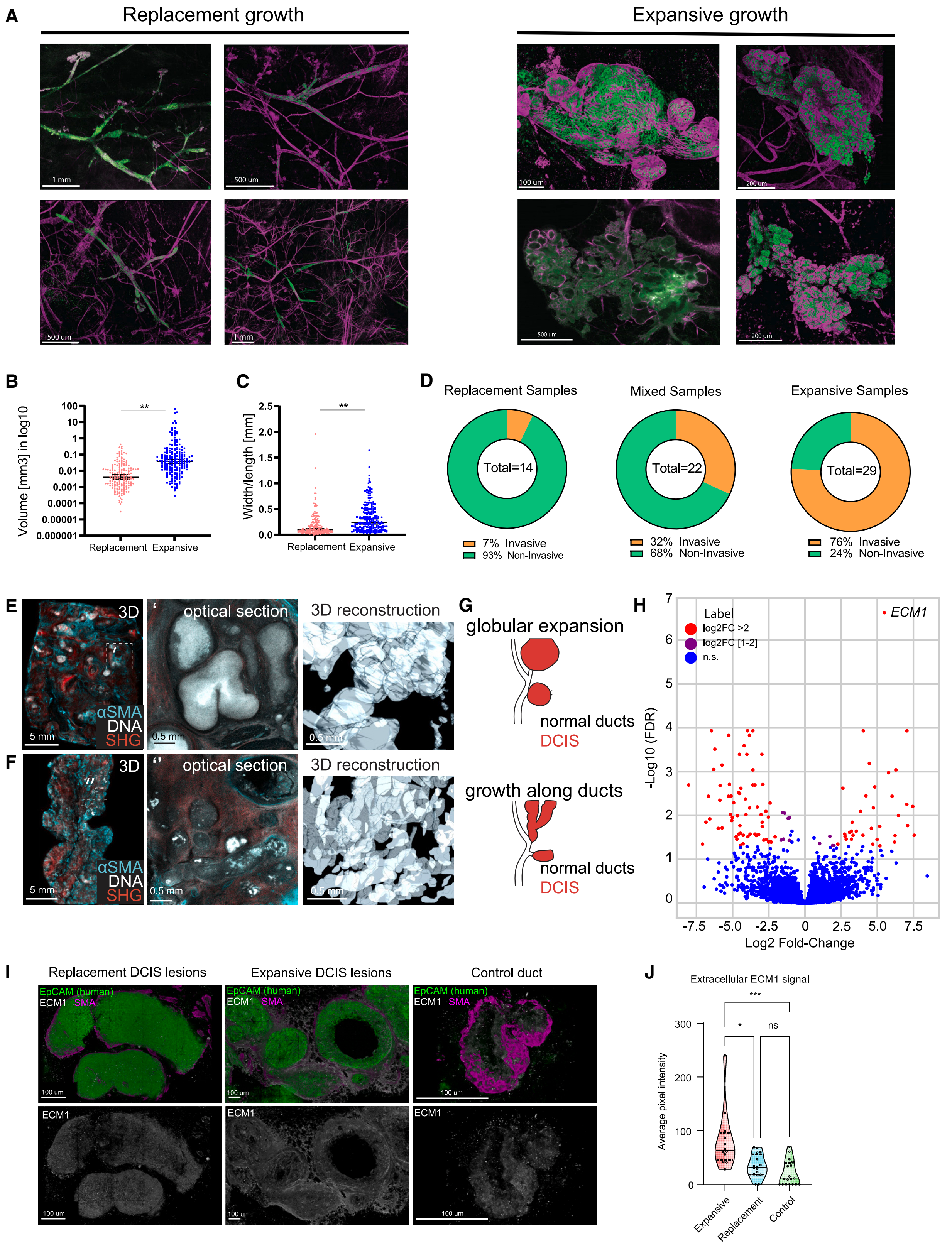

Figure 5. 3D imaging reveals two distinct DCIS growth patterns with strong correlation to invasive progression

(A) Examples of 3D whole-mount imaging of DCIS lesions in PDX models, showing replacement growth (left panel) and expansive growth (right panel). Cells of human origin are marked with Ku80 (green), myoepithelial cells are marked with αSMA (magenta).

(legend continued on next page)

Interestingly, the hyperbranched expansive lesions did not result in a significant difference in invasive growth compared with lesions without hyperbranching (Figures S5F and S5G). To assess whether these 3D growth patterns represent DCIS growth in patients we used a modified FLASH tissue-clearing protocol[29] to perform 3D imaging of FFPE DCIS patient samples, which revealed two similar growth patterns (Figures 5E–5G).

To find an explanation for the difference in 3D growth pattern, we compared gene expression profiles of replacement and expansive samples and found extracellular matrix protein 1 (ECM1) to be significantly upregulated in expansive lesions (Figure 5H). ECM1 is a secreted glycoprotein reported to be a marker of poor prognosis in multiple cancer types, including breast cancer.[30] We validated this result using immunofluorescence staining of ECM1 in thick tissue sections of DCIS-MIND lesions (Figure 5I). Interestingly, the overall ECM1 signal did not differ between lesions with replacement and expansive growth (Figure S5H). However, extracellular ECM1 was significantly more present in expansive lesions compared with replacement lesions and normal ducts (Figure 5J). Together, these results indicate that the 3D growth pattern is a potential predictor of invasive progression of DCIS, which could be driven by increased deposition of extracellular ECM1.

DCIS-MIND models show phenotypic and genotypic stability over multiple passages

To study whether DCIS samples evolve and acquire a more aggressive phenotype over time, we sequentially transplanted DCIS cells over multiple generations in MIND models. Six to 12 months after transplantation, the human DCIS cells were isolated from the injected mammary glands using magnetic bead sorting. This leads to an enrichment of human EpCAM$^+$ cells that were subsequently re-injected into NSG mice (Figures 6A and S6A). Of the 115 first-generation DCIS models (P0), we were able to successfully re-transplant 42 models (36%) to a second-generation (P1) (Figure 6B). For 16 of these models, a third-generation (P2) could be generated (Figure S6B).

Importantly, throughout these serial transplantations, intrinsic phenotypes such as molecular subtype, growth pattern, and invasive potential were maintained (Figures 6C–6E). Also, analysis of CNA and RNA-seq data from a subset of models revealed high stability between successive transplantations (Figures 6F and 6G). Only the expression profile of DCIS123 P0 and P1 did not show a good correlation, potentially caused by a heterogeneous lesion with the presence of both DCIS and LCIS. The limited phenotypic and genotypic changes over multiple generations are in line with the fact that synchronous DCIS-IBC have little genomic differences.[31,32] Therefore, our data strongly suggest that the propensity to become invasive is a DCIS-intrinsic phenotype that does not evolve over time.

In addition, we created a biobank of 19 DCIS-MIND models that can be distributed to other labs as we obtained more than 1 million cells after each generation of xenografts. This collection includes 2 luminal A, 4 luminal B, 2 ER$^+$HER2$^+$, and 11 ER$^-$HER2$^+$ DCIS models (Figures 6H and S6C). These distributable models are suited for future research into biomarkers and therapeutics for prevention of DCIS invasive progression. As an example, we tested estrogen dependence of four ER$^+$ models by transplanting them in mice that were either supplemented with E2 in the drinking water or deprived of estrogen by ovariectomy. For all four ER$^+$ models, DCIS lesions grew significantly faster in the estrogen-supplemented mice than in the ovariectomized mice, indicating that the DCIS-MIND models retained their estrogen sensitivity (Figures 6I and S6D–S6G). Intraductal growth can be difficult to assess via palpation and caliper measurements. Therefore, we introduced AkaLuciferase (AkaLuc) in the DCIS118 and DCIS088 models and could measure intraductal growth using bioluminescence imaging as early as 4 weeks after intraductal injection (Figures S6H and S6I).

HER2 expression promotes invasive progression of DCIS

The distributable DCIS-MIND models also permit experimental validation of candidate drivers of invasive progression of DCIS. HER2 expression was consistently found to be correlated with invasive progression in multiple analyses, including CNA, IHC, and RNA expression (Figures 3C, 4, and S4). To test whether HER2 overexpression also leads to downstream pathway activation, we performed IHC stainings for p-ERK and p-AKT (Figure 7A). The vast majority of HER2$^+$ DCIS (18 out of 19) showed high expression of p-ERK and/or p-AKT, while most HER2$^-$ controls (3 out of 4) did not (Figure 7B).

To investigate the effect of HER2 expression on DCIS progression, we tested the effect of HER2 inhibition in HER2$^+$ MIND

(B) Volumes of DCIS lesions for replacement and expansive growth. Data are represented as mean ± SEM. Two-tailed Mann-Whitney test. **p < 0.01.
(C) Width to length ratios of DCIS lesions for replacement and expansive growth, revealing a more elongated shape for lesions with replacement growth vs. a more spherical shape for lesions with expansive growth. Data are represented as mean ± SEM. Two tailed Mann Whitney test. **p < 0.01.
(D) Invasive progression rates of DCIS samples showing replacement growth, mixed growth, and expansive growth, respectively.
(E–G) 3D morphologies of FFPE blocks from patients with DCIS. FFPE blocks were tissue-cleared and imaged for αSMA (cyan), nuclei (gray), and second harmonics generation detecting collagen 1 (red).
(E) Example of globular DCIS morphology, representing expansive growth. Left, 3D visualization of the intact FFPE block. Middle, optical section through the indicated area in the 3D view. Right, 3D surface reconstruction of DCIS ducts in the indicated area.
(F) Example for tubular DCIS morphology, representing replacement growth. Left, 3D view of the intact FFPE block. Middle, optical section through the indicated area. Right, 3D surface reconstruction of the indicated area.
(G) Scheme illustrating DCIS morphologies in human resections that can be distinguished as globular expansion and tubular growth along ducts.
(H) Volcano plot of differentially expressed genes in DCIS lesions for replacement vs. expansive growth.
(I) Immunofluorescence staining of ECM1 (white) in a DCIS lesion showing replacement growth (top panels), a lesion showing expansive growth (middle panels), and a normal mammary duct (bottom panels). High extracellular expression of ECM1 protein is detected in the expansive DCIS lesion. Cells of human origin are marked with Ku80 (green), myoepithelial cells are marked with αSMA (magenta).
(J) Quantification of extracellular ECM1 expression in DCIS lesions for expansive or replacement growth and normal mammary ducts. Lines indicate Q^1, median, and Q^3. Two-tailed Mann-Whitney test. ***p < 0.001, *p < 0.05, ns, not significant. See also Figure S5.

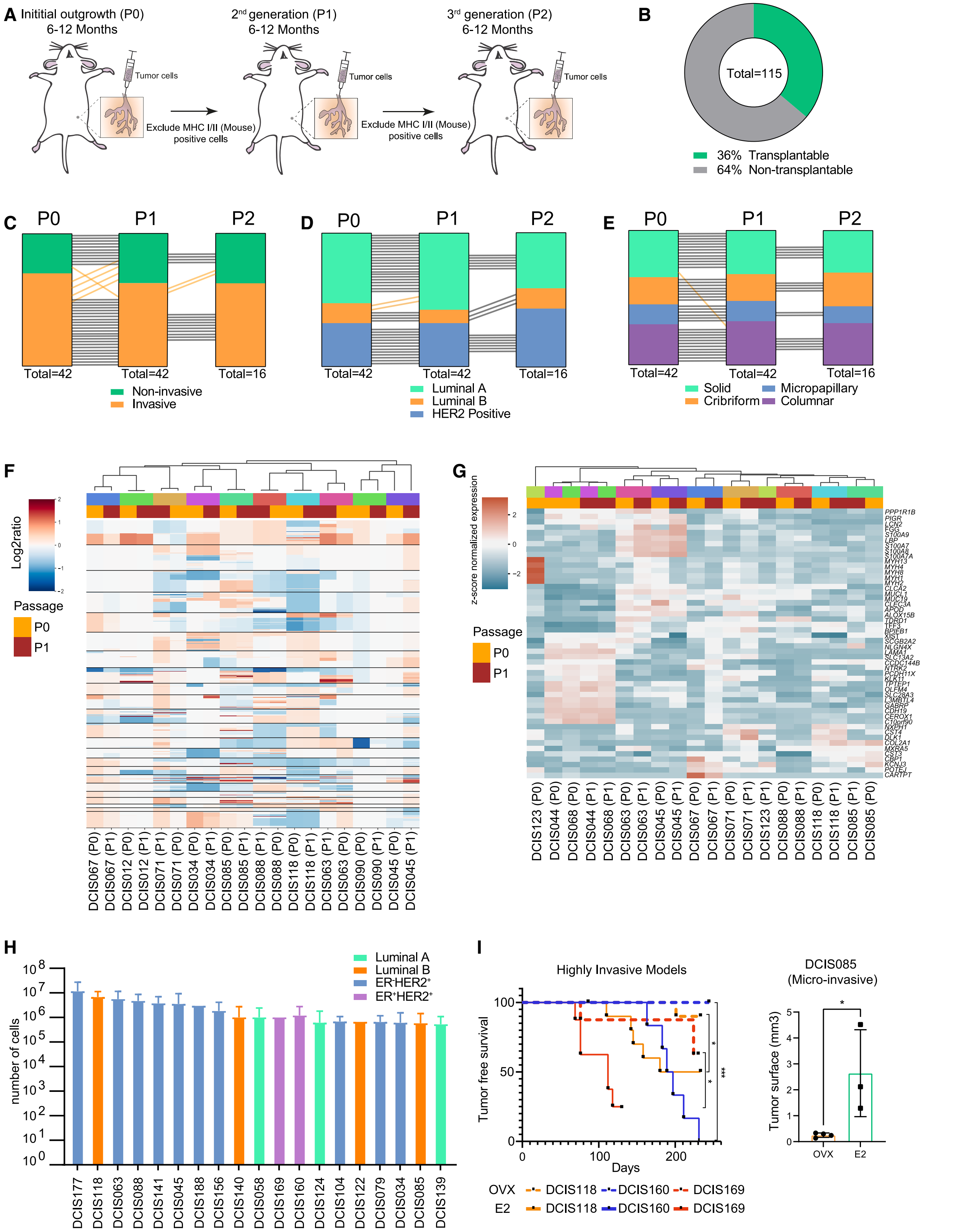

Figure 6. DCIS-MIND models retain stable phenotypes and genotypes over multiple passages

(A) Schematic representation of serial transplantation of DCIS outgrowths by magnetic bead sorting. Exclusion of mouse cells based on mouse-specific MHC class I and MHC class II.

(legend continued on next page)

models and conversely overexpressed HER2 in a HER2$^-$ MIND model. Treatment of seven invasive HER2$^+$ DCIS models with herceptin effectively prevented outgrowth of palpable tumors in 5 out of 7 models (Figure 7C and S7A–S7E). Only two models (DCIS156 and DCIS169) did not respond to herceptin treatment (Figures 7D and S7F–S7G). Whole-mount analyses revealed significant growth with invasion in the control group, while the responsive treatment groups showed few non-invasive and small lesions (Figure 7E).

In addition, we overexpressed HER2 by lentiviral transduction in a non-invasive HER2$^-$ DCIS model (DCIS098). Stable and functional overexpression of HER2 upon transduction was validated in HEK293T cells prior to transduction of DCIS098 cells (Figures 7F, 7G, and S7B). Six months after injection, whole-mount analyses revealed that the mock-treated cells grew as non-invasive replacement lesions, whereas the HER2-overexpressing cells grew as an invasive expansive lesion (Figure 7H). Together, these data demonstrate the causal role of HER2 expression in the invasive progression of DCIS and underscore the utility of DCIS-MIND models for functional validation studies.

DISCUSSION

Although DCIS accounts for 20%–25% of all newly diagnosed breast cancers, its natural progression is still poorly understood and consensus biomarkers for DCIS progression are lacking, prompting the need for preclinical *in vivo* models that permit longitudinal monitoring of DCIS growth and progression. Previous studies using cell lines such as MCF10DCIS.com and SUM225 or small collections of DCIS xenografts have not led to reliable biomarkers for DCIS progression, mainly because cell lines only model ER$^-$ DCIS and because the small number of available xenografts did not account for the heterogeneity of the disease.[13,33,34] Here, we report the generation of a large biobank of 115 orthotopic DCIS-MIND models recapitulating the molecular and histological heterogeneity of the patient population.[35,36] Monitoring of the natural progression of DCIS of our biobank showed that 46% of DCIS cases progress into invasive disease, suggesting that around half of the DCIS lesions in patients would stay indolent if left untreated. Moreover, we provide a collection of 19 distributable DCIS-MIND models, including luminal A, luminal B, ER$^+$HER2$^+$, and ER$^-$HER2$^+$ models. This diverse and phenotypically stable platform vastly increases the number and diversity of *in vivo* models of DCIS and fulfills the need for better models to study the natural evolution of DCIS. Finally, this extensively characterized biobank vastly increases the amount of omics data available for DCIS and offers the opportunity to identify and validate biomarkers related to invasive progression of DCIS.

Commonly studied biomarkers in DCIS include nuclear grade, comedonecrosis, tumor size, and expression of ER, PR, HER2, and Ki67, but this has so far not led to any consensus.[37–39] For example, there are conflicting reports regarding the role of HER2 expression in DCIS progression.[7,40–43] These studies focus on different outcomes including recurrence of DCIS or IBC and the samples analyzed have a large variation in treatment history, such as extent of surgery and adjuvant treatment. Our models do not have these limitations, and thus enabled us to identify multiple prognostic factors for invasive progression of DCIS, including solid growth, comedonecrosis, grade 3, ER and PR negativity, HER2 positivity, and high Ki67, whereas columnar growth or a luminal A subtype are indicative of low-risk DCIS. These findings may aid stratification of DCIS patients into high- and low-risk subgroups, as is currently being done in clinical trials such as LORIS, LORD, COMET, and LORETTA.[44–47] Indeed, our findings provide strong support for the decision to exclude patients with grade 3, comedonecrosis, or a HER2$^+$ subtype DCIS from active surveillance in these trials.

Comprehensive molecular profiling of all DCIS-MIND models suggests that 1q gain and 16q loss are necessary for DCIS initiation, while additional aberrations are needed for promoting invasive progression. These findings are in line with Geyer et al.,[48] who propose that low-grade DCIS have 1q gains and 16q losses, whereas more aggressive high-grade lesions have additional aberrations in for example 8q (*MYC*), 17q (*ERBB2*) or 20q (*PTK6*). Interestingly, our study identified significant CNAs and genes that strongly overlap with the non-significant CNAs identified by Strand et al.[15] (i.e., *MYC* (8q24), *ERBB2* (17q12), *PTK6* (20q13), *FGFR1* (8p11.23)). RNA-seq analyses further confirmed the correlation between risk of invasive progression and high expression of *ERBB2* (HER2), *PTK6* and *Ki67*, which have all been described previously as potential risk factors for tumor progression.[27,28,37,41,42] RNA-seq analyses also highlighted the relevance of DCIS recurrence classifiers (onco-type DX DCIS-, COX2$^+$P16$^+$ Ki67$^+$ and 812 gene classifier) for prediction of invasive progression in untreated DCIS. Altogether, this demonstrates the potential of our *in vivo* DCIS-MIND platform to identify significant associations for non-significant trends observed in human studies.

Beyond classical genomic and transcriptomic biomarkers, lesion morphology may provide another source of predictive information, as demonstrated by our observation that two distinct 3D growth patterns, i.e., replacement or expansive growth, correlate strongly with invasive progression and are able to predict invasive progression better than any other marker.

(B) Pie chart of success rate of transplantable DCIS models.

(C–E) Maintenance of invasive potential (C), molecular subtype (D), and growth pattern (E) of DCIS lesions during serial transplantation. A black line indicates concordance between passages, whereas an orange line indicates a discordance between passages.

(F and G) Unsupervised clustering of CNA (F) and gene expression (G) profiles of P0 and P1 outgrowths from 10 transplantable DCIS models, showing stable profiles during serial transplantation. Matched samples are color coded.

(H) Distribution of the average number of tumor cells harvested from DCIS passages of the 19 transplantable DCIS models, which yield more than 1,000,000 cells per passage and are therefore suitable for distribution to other labs. Data are represented as mean ± SEM.

(I) Effects of estrogen on *in vivo* growth of ER$^+$ DCIS lesions. Mice were either ovariectomized (dotted plots) or treated with estradiol via the drinking water (continuous plots). Left panel: tumor-free survival of three ER$^+$ models (DCIS118, DCIS160, and DCIS169) with log rank test. ***$p < 0.001$, *$p < 0.05$. Right panel: extent of tumor outgrowth in ER$^+$ DCIS model DCIS085. Data are represented as mean ± SEM. Unpaired two-tailed Student's t test. *$p < 0.05$. See also Figure S6.

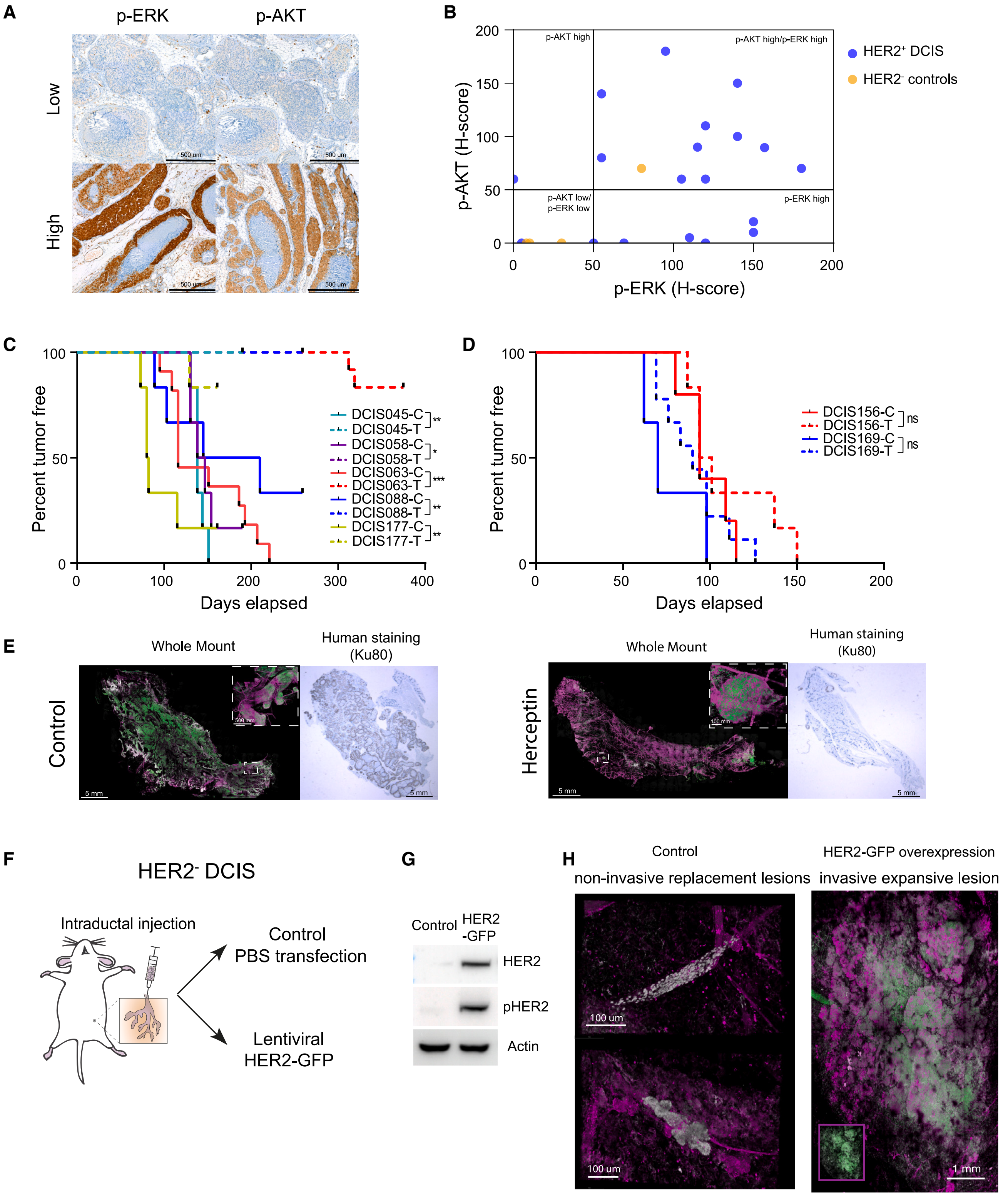

Figure 7. HER2 overexpression is a driver for invasive progression of DCIS

(A) Representative IHC images of p-ERK and p-AKT.

(B) Plotted H scores for p-ERK and p-AKT expression in HER2$^+$ DCIS and HER2$^-$ DCIS controls.

(C) Tumor-free survival curves of responsive HER2$^+$ DCIS models treated with vehicle (C) or herceptin (T) with log rank test. ***$p < 0.001$, **$p < 0.01$, *$p < 0.05$.

(D) Tumor-free survival curves of non-responsive HER2$^+$ DCIS models treated with vehicle (C) or herceptin (T) with log rank test. ***$p < 0.001$, **$p < 0.01$, *$p < 0.05$.

(legend continued on next page)

Importantly, we show that similar growth patterns also occur in DCIS specimens from patients, indicating that 3D pathology of human breast cancers could yield prognostic biomarkers which cannot be uncovered with 2D pathology.[49,50]

The capability to monitor *in vivo* evolution of DCIS for prolonged periods of time via serial transplantation enabled us to evaluate the different evolutionary models that have been proposed. The independent evolutionary model postulates that DCIS and IBC evolve in parallel and do not necessarily share genomic aberrations, suggesting an independent origin of DCIS and IBC.[51,52] In contrast, the evolutionary bottleneck model postulates that multiple DCIS clones co-exist, after which a single clone acquires the propensity to break through the basement membrane and expand into an invasive tumor mass. This model is supported by several studies showing overlapping mutations and CNAs between DCIS and adjacent IBC.[31,53–55] Single-cell sequencing studies found evidence for a multiclonal invasion model, in which multiple cell lineages are able to invade into the stroma after the degradation of the basement membrane.[32,56]

Our MIND models show remarkable genetic and phenotypic stability during serial passaging, which is concordant with previous data showing that synchronous DCIS-IBC have marginal genomic differences.[31,32] Importantly, also the invasive behavior of our DCIS-MIND models remains stable during serial passaging, showing that the ability of DCIS lesions to become invasive is an intrinsic phenotype that does not evolve over time, thus lending strong support to the multiclonal evolutionary model[32]

In conclusion, this work provides a large, well-characterized resource of patient-derived *in vivo* models recapitulating the full heterogeneity of DCIS. These models may facilitate the identification and validation of biomarkers related to invasive progression of DCIS, and thereby foster the development of more tailored treatment strategies for DCIS patients.

Limitations of the study

An important limitation of our DCIS-MIND models is the lack of immune cells and human stroma as our model requires immunodeficient mice. Multiple studies have stressed the importance of the tumor stroma and immune infiltrate and have shown correlations between the tumor microenvironment and the progression of DCIS.[57,58] Efforts have been made to introduce human immune cells to immune deficient mouse models, but these do not fully recapitulate the human immune system.[59,60] In addition, we are limited to the 1- to 2-year lifespan of our mouse models, whereas the progression of DCIS in women can take years or even decades.

Finally, the slow growth kinetics of specifically non-invasive DCIS models limits the possibilities of growing large numbers of cells for additional experiments and multi-omics analyses. These analyses are therefore most likely slightly biased toward faster-growing and larger DCIS lesions.

STAR★METHODS

Detailed methods are provided in the online version of this paper and include the following:

- KEY RESOURCES TABLE
- RESOURCE AVAILABILITY
  - Lead contact
  - Materials availability
  - Data and code availability
- EXPERIMENTAL MODEL AND SUBJECT DETAILS
  - Cell lines
  - Mice
  - Human samples
- METHOD DETAILS
  - Patient derived DCIS xenograft models
  - Magnetic bead sorting and re-transplantation
  - Estrogen sensitivity of ER+ DCIS-MIND models
  - Herceptin (traztuzumab) treatment of mice
  - Lentiviral expression in human DCIS of ERBB2 or Aka-luciferase
  - In vivo bioluminescence imaging
  - Immunohistochemistry (IHC)
  - RAD51 assay (immunofluorescence staining and scoring)
  - Whole-mount immunofluorescence staining of mammary glands
  - Whole-mount imaging of mammary glands
  - Immunofluorescent labeling of thick tissue sections
  - 3D imaging of human FFPE DCIS resections
  - Pathology
  - Microdissection
  - CNA-seq
  - Whole exome sequencing & panel sequencing
  - Whole genome sequencing
  - RNA-seq
  - RNAseq classifiers
- QUANTIFICATION AND STATISTICAL ANALYSIS

SUPPLEMENTAL INFORMATION

Supplemental information can be found online at https://doi.org/10.1016/j.ccell.2023.04.002.

ACKNOWLEDGMENTS

This work was supported by Cancer Research UK and by the Dutch Cancer Society (KWF) (ref. C38317/A24043). This work is part of the Oncode Institute,

(E) Representative whole-mount images and Ku80-stained sections of DCIS-injected mammary glands from DCIS063 mice treated with vehicle (left panels) or herceptin (right panels).

(F) Schematic representation of the experimental setup for lentiviral overexpression of HER2 in HER2⁻ DCIS cells.

(G) Western blot showing expression of HER2 and phospho-HER2 in parental 293T cells and 293T cells transduced with the HER2-GFP lentivirus.

(H) Representative whole-mount images of intraductally injected mammary glands, showing non-invasive replacement growth of non-transduced HER2⁻ DCIS cells (left panel) and invasive expansive growth of the same DCIS cells transduced with the HER2-GFP lentivirus (right panel). Inset shows lesion without αSMA marker indicating GFP expression (green). Cells of human origin are marked with Ku80 (gray), myoepithelial cells are marked with αSMA (magenta). Inset shows lesion without αSMA marker indicating HER2-GFP expression (green). See also Figure S7.

which is partly financed by the KWF. Research at the Netherlands Cancer Institute is supported by institutional grants from the KWF and the Dutch Ministry of Health, Welfare and Sport. We wish to thank Zoya Kingsbury and Jordi Denecker, Illumina Cambridge Ltd, for performing WGS; Shinae Kizaka-Kondoh for providing the Akaluciferase plasmid; Frederieke van Duijnhoven for help with the informed consent; and Ellen Tanger, Eva Schut-Kregel, Milo van Batenburg, and Tom van Lint for technical support. Numeric analysis was partially carried out using the supercomputer cluster "Afalina" in Sevastopol State University. We also wish to acknowledge the NKI Core Facility Molecular Pathology & Biobanking (CFMPB); Genomics Core facility (GCF), specifically Roel Kluin and Charlaine van Steenis; the human and mouse pathology facilities; and the caretakers of the animal facility. We are most grateful to all patients who donated tissue samples. Finally, we thank the Grand Challenge PRECISION Consortium Steering Group: Jelle Wesseling, Jos Jonkers, Jacco van Rheenen, Esther H. Lips, Marjanka Schmidt, Lodewyk F.A. Wessels, Proteeti Bhattacharjee (Netherlands Cancer Institute, Amsterdam, The Netherlands), Alastair Thompson (Baylor College of Medicine, Houston, Texas, USA), Serena Nik-Zainal, Helen Davies (University of Cambridge, Cambridge, UK), Elinor J. Sawyer (King's College London, London, UK), Andrew Futreal, Nicholas Navin (MD Anderson Cancer Center, Houston, TX, USA), E. Shelley Hwang (Duke University School of Medicine, Durham, NC, USA), Fariba Behbod (Kansas University Medical Center, KS, USA), Daniel Rea (University of Birmingham, Birmingham, UK), Hilary Stobart (Independent Cancer Patients' Voice, UK), Deborah Collyar (Patient Advocates in Research, USA), Donna Pinto (dcis411, USA), Ellen Verschuur, Marja van Oirsouw (Borstkanker Vereniging Nederland, the Netherlands).

AUTHOR CONTRIBUTIONS

Conceptualization, S.J.H., J.J., and J.W.; methodology, S.J.H., F.B., and J.J.; formal analyses, S.J.H. and R.d.B., and M.S.; investigation, S.J.H., C.L., M.B., T.E., X.C., M.C., H.M., A.H.-R., P.K., L.M., R.v.d.W., J.S., M.M.A., A.L.-G., and H.R.D.; resources, M.J.v.H. and N.I.M.; writing – original draft, S.J.H. and C.L.G.J.S.; writing – review & editing, all authors; visualization, S.J.H., M.C., H.M., and C.L.G.J.S.; supervision, S.N.-K., V.S., J.v.R., E.H.L., L.F.A.W., J.W., C.L.G.J.S., and J.J.; funding acquisition, J.W. and J.J.

DECLARATION OF INTERESTS

The authors declare no competing interests.

Received: August 16, 2022
Revised: February 21, 2023
Accepted: April 4, 2023
Published: April 27, 2023

REFERENCES

1. Netherlands Comprehensive Cancer Organisation. (2016). www.cijfersoverkanker.nl.
2. (2017). Cancer research UK. http://www.cancerresearchuk.org/health-professional/cancer-statistics/statistics-by-cancer-type/breast-cancer/incidence-in-situ.
3. Siegel, R.L., Miller, K.D., and Jemal, A. (2020). Cancer statistics, 2020. CA A Cancer J. Clin. *70*, 7–30. https://doi.org/10.3322/caac.21590.
4. van Seijen, M., Lips, E.H., Thompson, A.M., Nik-Zainal, S., Futreal, A., Hwang, E.S., Verschuur, E., Lane, J., Jonkers, J., Rea, D.W., et al. (2019). Ductal carcinoma in situ: to treat or not to treat, that is the question. Br. J. Cancer *121*, 285–292. https://doi.org/10.1038/s41416-019-0478-6.
5. Gorringe, K.L., Hunter, S.M., Pang, J.M., Opeskin, K., Hill, P., Rowley, S.M., Choong, D.Y.H., Thompson, E.R., Dobrovic, A., Fox, S.B., et al. (2015). Copy number analysis of ductal carcinoma in situ with and without recurrence. Mod. Pathol. *28*, 1174–1184. https://doi.org/10.1038/modpathol.2015.75.
6. Pareja, F., Brown, D.N., Lee, J.Y., Da Cruz Paula, A., Selenica, P., Bi, R., Geyer, F.C., Gazzo, A., da Silva, E.M., Vahdatinia, M., et al. (2020). Whole-exome sequencing analysis of the progression from non–low-grade ductal carcinoma in situ to invasive ductal carcinoma. Clin. Cancer Res. *26*, 3682–3693. https://doi.org/10.1158/1078-0432.CCR-19-2563.
7. Lin, C.Y., Vennam, S., Purington, N., Lin, E., Varma, S., Han, S., Desa, M., Seto, T., Wang, N.J., Stehr, H., et al. (2019). Genomic landscape of ductal carcinoma in situ and association with progression. Breast Cancer Res. Treat. *178*, 307–316. https://doi.org/10.1007/s10549-019-05401-x.
8. Behbod, F., Gomes, A.M., and Machado, H.L. (2018). Modeling human ductal carcinoma in situ in the mouse. J. Mammary Gland Biol. Neoplasia *23*, 269–278. https://doi.org/10.1007/s10911-018-9408-0.
9. Valdez, K.E., Fan, F., Smith, W., Allred, D.C., Medina, D., and Behbod, F. (2011). Human primary ductal carcinoma in situ (DCIS) subtype-specific pathology is preserved in a mouse intraductal (MIND) xenograft model. J. Pathol. *225*, 565–573. https://doi.org/10.1002/path.2969.
10. Behbod, F., Kittrell, F.S., LaMarca, H., Edwards, D., Kerbawy, S., Heestand, J.C., Young, E., Mukhopadhyay, P., Yeh, H.W., Allred, D.C., et al. (2009). An intraductal human-in-mouse transplantation model mimics the subtypes of ductal carcinoma in situ. Breast Cancer Res. *11*, R66. https://doi.org/10.1186/bcr2358.
11. Russell, T.D., Jindal, S., Agunbiade, S., Gao, D., Troxell, M., Borges, V.F., and Schedin, P. (2015). Myoepithelial cell differentiation markers in ductal carcinoma in situ progression. Am. J. Pathol. *185*, 3076–3089. https://doi.org/10.1016/j.ajpath.2015.07.004.
12. Sflomos, G., Dormoy, V., Metsalu, T., Jeitziner, R., Battista, L., Scabia, V., Raffoul, W., Delaloye, J.F., Treboux, A., Fiche, M., et al. (2016). A preclinical model for ERα-positive breast cancer points to the epithelial microenvironment as determinant of luminal phenotype and hormone response. Cancer Cell *29*, 407–422. https://doi.org/10.1016/j.ccell.2016.02.002.
13. Hong, Y., Limback, D., Elsarraj, H.S., Harper, H., Haines, H., Hansford, H., Ricci, M., Kaufman, C., Wedlock, E., Xu, M., et al. (2022). Mouse-INtraDuctal (MIND): an in vivo model for studying the underlying mechanisms of DCIS malignancy. J. Pathol. *256*, 186–201. https://doi.org/10.1002/path.5820.
14. Makki, J. (2015). Diversity of breast carcinoma: histological subtypes and clinical relevance. Clin. Med. Insights Pathol. *8*, 23–31. https://doi.org/10.4137/CPath.S31563.
15. Strand, S.H., Rivero-Gutiérrez, B., Houlahan, K.E., Seoane, J.A., King, L.M., Risom, T., Simpson, L.A., Vennam, S., Khan, A., Cisneros, L., et al. (2022). Molecular classification and biomarkers of clinical outcome in breast ductal carcinoma in situ: analysis of TBCRC 038 and RAHBT cohorts. Cancer Cell *40*, 1521–1536.e7. https://doi.org/10.1016/j.ccell.2022.10.021.
16. Lips, E.H., Kumar, T., Megalios, A., Visser, L.L., Sheinman, M., Fortunato, A., Shah, V., Hoogstraat, M., Sei, E., Mallo, D., et al. (2022). Genomic analysis defines clonal relationships of ductal carcinoma in situ and recurrent invasive breast cancer. Nat. Genet. *54*, 850–860. https://doi.org/10.1038/s41588-022-01082-3.
17. Davies, H., Glodzik, D., Morganella, S., Yates, L.R., Staaf, J., Zou, X., Ramakrishna, M., Martin, S., Boyault, S., Sieuwerts, A.M., et al. (2017). HRDetect is a predictor of BRCA1 and BRCA2 deficiency based on mutational signatures. Nat. Med. *23*, 517–525. https://doi.org/10.1038/nm.4292.
18. Bernard, P.S., Mullins, M., Cheang, M.C.U., Leung, S., Voduc, D., Vickery, T., Davies, S., Fauron, C., He, X., Hu, Z., et al. (2009). Supervised risk predictor of breast cancer based on intrinsic subtypes. J. Clin. Oncol. *27*, 1160–1167. https://doi.org/10.1200/JCO.2008.18.1370.
19. Zhao, X., Rødland, E.A., Tibshirani, R., and Plevritis, S. (2015). Molecular subtyping for clinically defined breast cancer subgroups. Breast Cancer Res. *17*, 29. https://doi.org/10.1186/s13058-015-0520-4.
20. Solin, L.J., Gray, R., Baehner, F.L., Butler, S.M., Hughes, L.L., Yoshizawa, C., Cherbavaz, D.B., Shak, S., Page, D.L., Sledge, G.W., et al. (2013). A multigene expression assay to predict local recurrence risk for ductal carcinoma in situ of the breast. J. Natl. Cancer Inst. *105*, 701–710. https://doi.org/10.1093/jnci/djt067.
21. Kerlikowske, K., Molinaro, A.M., Gauthier, M.L., Berman, H.K., Waldman, F., Bennington, J., Sanchez, H., Jimenez, C., Stewart, K., Chew, K., et al. (2010). Biomarker expression and risk of subsequent tumors after initial

CellPress
OPEN ACCESS

ductal carcinoma in situ diagnosis. J. Natl. Cancer Inst. *102*, 627–637. https://doi.org/10.1093/jnci/djq101.

22. Molinaro, A.M., Sison, J.D., Ljung, B.-M., Tlsty, T.D., and Kerlikowske, K. (2016). Risk prediction for local versus regional/metastatic tumors after initial ductal carcinoma in situ diagnosis treated by lumpectomy. Breast Cancer Res. Treat. *157*, 351–361. https://doi.org/10.1007/s10549-016-3814-z.
23. Bao, Y.I., Wang, A., and Mo, J. (2016). S100A8/A9 is associated with estrogen receptor loss in breast cancer. Oncol. Lett. *11*, 1936–1942. https://doi.org/10.3892/ol.2016.4134.
24. Wang, D., Liu, G., Wu, B., Chen, L., Zeng, L., and Pan, Y. (2018). Clinical significance of elevated S100A8 expression in breast cancer patients. Front. Oncol. *8*, 496. https://doi.org/10.3389/fonc.2018.00496.
25. Zhao, Y.-F., Zhao, J.-Y., Yue, H., Hu, K.-S., Shen, H., Guo, Z.-G., and Su, X.-J. (2015). FOXD1 promotes breast cancer proliferation and chemotherapeutic drug resistance by targeting p27. Biochem. Biophys. Res. Commun. *456*, 232–237. https://doi.org/10.1016/j.bbrc.2014.11.064.
26. de Ronde, J.J., Rigaill, G., Rottenberg, S., Rodenhuis, S., and Wessels, L.F.A. (2013). Identifying subgroup markers in heterogeneous populations. Nucleic Acids Res. *41*, e200. https://doi.org/10.1093/nar/gkt845.
27. Park, S.H., Ito, K., Olcott, W., Katsyv, I., Halstead-Nussloch, G., and Irie, H.Y. (2015). PTK6 inhibition promotes apoptosis of Lapatinib-resistant Her2 + breast cancer cells by inducing Bim. Breast Cancer Res. *17*, 1–13. https://doi.org/10.1186/s13058-015-0594-z.
28. Ito, K., Park, S.H., Katsyv, I., Zhang, W., de Angelis, C., Schiff, R., and Irie, H.Y. (2017). ARTICLE OPEN PTK6 regulates growth and survival of endocrine therapy-resistant ER+ breast cancer cells. NPJ Breast Cancer *3*, 45. https://doi.org/10.1038/s41523-017-0047-1.
29. Messal, H.A., Almagro, J., Zaw Thin, M., Tedeschi, A., Ciccarelli, A., Blackie, L., Anderson, K.I., Miguel-Aliaga, I., van Rheenen, J., and Behrens, A. (2021). Antigen retrieval and clearing for whole-organ immunofluorescence by FLASH. Nat. Protoc. *16*, 239–262. https://doi.org/10.1038/s41596-020-00414-z.
30. Lal, G., Hashimi, S., Smith, B.J., Lynch, C.F., Zhang, L., Robinson, R.A., and Weigel, R.J. (2009). Extracellular matrix 1 (ECM1) expression is a novel prognostic marker for poor long-term survival in breast cancer: a hospital-based cohort study in Iowa. Ann. Surg Oncol. *16*, 2280–2287. https://doi.org/10.1245/s10434-009-0533-2.
31. Kim, S.Y., Jung, S.-H., Kim, M.S., Baek, I.-P., Lee, S.H., Kim, T.-M., Chung, Y.-J., and Lee, S.H. (2015). Genomic differences between pure ductal carcinoma in situ and synchronous ductal carcinoma in situ with invasive breast cancer. Oncotarget *6*, 7597–7607. https://doi.org/10.18632/oncotarget.3162.
32. Casasent, A.K., Schalck, A., Gao, R., Sei, E., Long, A., Pangburn, W., Casasent, T., Meric-Bernstam, F., Edgerton, M.E., and Navin, N.E. (2018). Multiclonal invasion in breast tumors identified by topographic single cell sequencing. Cell *172*, 205–217.e12. https://doi.org/10.1016/j.cell.2017.12.007.
33. Miller, F.R., Santner, S.J., Tait, L., and Dawson, P.J. (2000). MCF10DCIS.com xenograft model of human comedo ductal carcinoma in situ. J. Natl. Cancer Inst. *92*, 1185a–1186a. https://doi.org/10.1093/jnci/92.14.1185a.
34. Forozan, F., Veldman, R., Ammerman, C.A., Parsa, N.Z., Kallioniemi, A., Kallioniemi, O.-P., and Ethier, S.P. (1999). Molecular cytogenetic analysis of 11 new breast cancer cell lines. Br. J. Cancer *81*, 1328–1334. https://doi.org/10.1038/sj.bjc.6695007.
35. Pang, J.M.B., Savas, P., Fellowes, A.P., Mir Arnau, G., Kader, T., Vedururu, R., Hewitt, C., Takano, E.A., Byrne, D.J., Choong, D.Y., et al. (2017). Breast ductal carcinoma in situ carry mutational driver events representative of invasive breast cancer. Mod. Pathol. *30*, 952–963. https://doi.org/10.1038/modpathol.2017.21.
36. Lips, E.H., Kumar, T., Megalios, A., Visser, L.L., Sheinman, M., Fortunato, A., Shah, V., Hoogstraat, M., Sei, E., Mallo, D., et al. (2022). Genomic analysis defines clonal relationships of ductal carcinoma in situ and recurrent invasive breast cancer. Nat. Genet. *54*, 850–860. https://doi.org/10.1038/s41588-022-01082-3.
37. Lari, S.A., and Kuerer, H.M. (2011). Biological markers in DCIS and risk of breast recurrence: a systematic review. J. Cancer *2*, 232–261.
38. Newman, L.A., and Bensenhaver, J.M. (2015). Ductal Carcinoma in Situ and Microinvasive/borderline Breast Cancer (Springer). https://doi.org/10.1007/978-1-4939-2035-8.
39. Visser, L.L., Groen, E.J., van Leeuwen, F.E., Lips, E.H., Schmidt, M.K., and Wesseling, J. (2019). Predictors of an invasive breast cancer recurrence after DCIS: a systematic review and meta-analyses. Cancer Epidemiol. Biomarkers Prev. *28*, 835–845. https://doi.org/10.1158/1055-9965.EPI-18-0976.
40. Thorat, M.A., Levey, P.M., Jones, J.L., Pinder, S.E., Bundred, N.J., Fentiman, I.S., and Cuzick, J. (2021). Prognostic and predictive value of HER2 expression in ductal carcinoma *in situ* : results from the UK/ANZ DCIS randomized trial. Clin. Cancer Res. *27*, 5317–5324. https://doi.org/10.1158/1078-0432.CCR-21-1239.
41. Roses, R.E., Paulson, E.C., Sharma, A., Schueller, J.E., Nisenbaum, H., Weinstein, S., Fox, K.R., Zhang, P.J., and Czerniecki, B.J. (2009). HER-2/neu overexpression as a predictor for the transition from *in situ* to invasive breast cancer. Cancer Epidemiol. Biomarkers Prev. *18*, 1386–1389. https://doi.org/10.1158/1055-9965.EPI-08-1101.
42. Liao, N., Zhang, G.c., Liu, Y.h., Li, X.r., Yao, M., Xu, F.p., Li, L., and Wu, Y.l. (2011). HER2-positive status is an independent predictor for coexisting invasion of ductal carcinoma in situ of the breast presenting extensive DCIS component. Pathol. Res. Pract. *207*, 1–7. https://doi.org/10.1016/J.PRP.2010.08.005.
43. Borgquist, S., Zhou, W., Jirström, K., Amini, R.M., Sollie, T., Sørlie, T., Blomqvist, C., Butt, S., and Wärnberg, F. (2015). The prognostic role of HER2 expression in ductal breast carcinoma in situ (DCIS); a population-based cohort study. BMC Cancer *15*, 468. https://doi.org/10.1186/s12885-015-1479-3.
44. Kanbayashi, C., and Iwata, H. (2017). Current approach and future perspective for ductal carcinoma in situ of the breast. Jpn. J. Clin. Oncol. *47*, 671–677. https://doi.org/10.1093/jjco/hyx059.
45. Elshof, L.E., Tryfonidis, K., Slaets, L., van Leeuwen-Stok, A.E., Skinner, V.P., Dif, N., Pijnappel, R.M., Bijker, N., Rutgers, E.J.T., and Wesseling, J. (2015). Feasibility of a prospective, randomised, open-label, international multicentre, phase III, non-inferiority trial to assess the safety of active surveillance for low risk ductal carcinoma in situ - the LORD study. Eur. J. Cancer *51*, 1497–1510. https://doi.org/10.1016/j.ejca.2015.05.008.
46. Hwang, E.S., Hyslop, T., Lynch, T., Frank, E., Pinto, D., Basila, D., Collyar, D., Bennett, A., Kaplan, C., Rosenberg, S., et al. (2019). The COMET (Comparison of Operative versus Monitoring and Endocrine Therapy) trial: a phase III randomised controlled clinical trial for low-risk ductal carcinoma in situ (DCIS). BMJ Open *9*, e026797. https://doi.org/10.1136/bmjopen-2018-026797.
47. Francis, A., Thomas, J., Fallowfield, L., Wallis, M., Bartlett, J.M.S., Brookes, C., Roberts, T., Pirrie, S., Gaunt, C., Young, J., et al. (2015). Addressing overtreatment of screen detected DCIS; the LORIS trial. Eur. J. Cancer *51*, 2296–2303. https://doi.org/10.1016/j.ejca.2015.07.017.
48. Lopez-Garcia, M.A., Geyer, F.C., Marchió, M., Marchió, C., and Reis-Filho, J.S. (2010). Breast cancer precursors revisited: molecular features and progression pathways. Histopathology *57*, 171–192. https://doi.org/10.1111/j.1365-2559.2010.03568.x.
49. Liu, J.T.C., Glaser, A.K., Bera, K., True, L.D., Reder, N.P., Eliceiri, K.W., and Madabhushi, A. (2021). Harnessing non-destructive 3D pathology. Nat. Biomed. Eng. *5*, 203–218. https://doi.org/10.1038/s41551-020-00681-x.
50. Almagro, J., Messal, H.A., Zaw Thin, M., van Rheenen, J., and Behrens, A. (2021). Tissue clearing to examine tumour complexity in three dimensions. Nat. Rev. Cancer *21*, 718–730. https://doi.org/10.1038/s41568-021-00382-w.
51. Miron, A., Varadi, M., Carrasco, D., Li, H., Luongo, L., Kim, H.J., Park, S.Y., Cho, E.Y., Lewis, G., Kehoe, S., et al. (2010). PIK3CA mutations in in situ and invasive breast carcinomas. Cancer Res. *70*, 5674–5678. https://doi.org/10.1158/0008-5472.CAN-08-2660.

52. Yates, L.R., Gerstung, M., Knappskog, S., Desmedt, C., Gundem, G., van Loo, P., Aas, T., Alexandrov, L.B., Larsimont, D., Davies, H., et al. (2015). Subclonal diversification of primary breast cancer revealed by multiregion sequencing. Nat. Med. *21*, 751–759. https://doi.org/10.1038/nm.3886.

53. Hernandez, L., Wilkerson, P.M., Lambros, M.B., Campion-Flora, A., Rodrigues, D.N., Gauthier, A., Cabral, C., Pawar, V., Mackay, A., A'Hern, R., et al. (2012). Genomic and mutational profiling of ductal carcinomas in situ and matched adjacent invasive breast cancers reveals intra-tumour genetic heterogeneity and clonal selection. J. Pathol. *227*, 42–52. https://doi.org/10.1002/path.3990.

54. Krøigård, A.B., Larsen, M.J., Lænkholm, A.V., Knoop, A.S., Jensen, J.D., Bak, M., Mollenhauer, J., Kruse, T.A., and Thomassen, M. (2015). Clonal expansion and linear genome evolution through breast cancer progression from pre-invasive stages to asynchronous metastasis. Oncotarget *6*, 5634–5649. https://doi.org/10.18632/oncotarget.3111.

55. Cowell, C.F., Weigelt, B., Sakr, R.A., Ng, C.K.Y., Hicks, J., King, T.A., and Reis-Filho, J.S. (2013). Progression from ductal carcinoma in situ to invasive breast cancer: revisited. Mol. Oncol. *7*, 859–869. https://doi.org/10.1016/j.molonc.2013.07.005.

56. Martelotto, L.G., Baslan, T., Kendall, J., Geyer, F.C., Burke, K.A., Spraggon, L., Piscuoglio, S., Chadalavada, K., Nanjangud, G., Ng, C.K.Y., et al. (2017). Whole-genome single-cell copy number profiling from formalin-fixed paraffin-embedded samples. Nat. Med. *23*, 376–385. https://doi.org/10.1038/nm.4279.

57. Gil Del Alcazar, C.R., Huh, S.J., Ekram, M.B., Trinh, A., Liu, L.L., Beca, F., Zi, X., Kwak, M., Bergholtz, H., Su, Y., et al. (2017). Immune escape in breast cancer during *in situ* to invasive carcinoma transition. Cancer Discov. *7*, 1098–1115. https://doi.org/10.1158/2159-8290.CD-17-0222.

58. Risom, T., Glass, D.R., Averbukh, I., Liu, C.C., Baranski, A., Kagel, A., McCaffrey, E.F., Greenwald, N.F., Rivero-Gutiérrez, B., Strand, S.H., et al. (2022). Transition to invasive breast cancer is associated with progressive changes in the structure and composition of tumor stroma. Cell *185*, 299–310.e18. https://doi.org/10.1016/j.cell.2021.12.023.

59. Wang, M., Yao, L.C., Cheng, M., Cai, D., Martinek, J., Pan, C.X., Shi, W., Ma, A.H., De Vere White, R.W., Airhart, S., et al. (2018). Humanized mice in studying efficacy and mechanisms of PD-1-targeted cancer immunotherapy. Faseb. J. *32*, 1537–1549. https://doi.org/10.1096/fj.201700740R.

60. Capasso, A., Lang, J., Pitts, T.M., Jordan, K.R., Lieu, C.H., Davis, S.L., Diamond, J.R., Kopetz, S., Barbee, J., Peterson, J., et al. (2019). Characterization of immune responses to anti-PD-1 mono and combination immunotherapy in hematopoietic humanized mice implanted with tumor xenografts. J. Immunother. Cancer *7*, 37. https://doi.org/10.1186/s40425-019-0518-z.

61. Martin, M. (2011). Cutadapt removes adapter sequences from high-throughput sequencing reads. EMBnet. j. *17*, 10. https://doi.org/10.14806/ej.17.1.200.

62. Li, H., and Durbin, R. (2009). Fast and accurate short read alignment with Burrows-Wheeler transform. Bioinformatics *25*, 1754–1760. https://doi.org/10.1093/bioinformatics/btp324.

63. Ahdesmäki, M.J., Gray, S.R., Johnson, J.H., and Lai, Z. (2017). Disambiguate: an open-source application for disambiguating two species in next generation sequencing data from grafted samples. F1000Res. *5*, 2741. https://doi.org/10.12688/f1000research.10082.2.

64. Andrews, S. (2010). FastQC: a quality control tool for high throughput sequence data. https://www.bioinformatics.babraham.ac.uk/projects/fastqc/.

65. Li, H., Handsaker, B., Wysoker, A., Fennell, T., Ruan, J., Homer, N., Marth, G., Abecasis, G., and Durbin, R.; 1000 Genome Project Data Processing Subgroup (2009). The sequence alignment/map format and SAMtools. Bioinformatics *25*, 2078–2079. https://doi.org/10.1093/bioinformatics/btp352.

66. Ewels, P., Magnusson, M., Lundin, S., and Käller, M. (2016). MultiQC: summarize analysis results for multiple tools and samples in a single report. Bioinformatics *32*, 3047–3048. https://doi.org/10.1093/bioinformatics/btw354.

67. Scheinin, I., Sie, D., Bengtsson, H., van de Wiel, M.A., Olshen, A.B., van Thuijl, H.F., van Essen, H.F., Eijk, P.P., Rustenburg, F., Meijer, G.A., et al. (2014). DNA copy number analysis of fresh and formalin-fixed specimens by shallow whole-genome sequencing with identification and exclusion of problematic regions in the genome assembly. Genome Res. *24*, 2022–2032. https://doi.org/10.1101/gr.175141.114.

68. Köster, J., and Rahmann, S. (2018). Snakemake—a scalable bioinformatics workflow engine. Bioinformatics *34*, 3600. https://doi.org/10.1093/bioinformatics/bty350.

69. Mermel, C.H., Schumacher, S.E., Hill, B., Meyerson, M.L., Beroukhim, R., and Getz, G. (2011). GISTIC2.0 facilitates sensitive and confident localization of the targets of focal somatic copy-number alteration in human cancers. Genome Biol. *12*, R41. https://doi.org/10.1186/gb-2011-12-4-r41.

70. Liao, Y., Smyth, G.K., and Shi, W. (2014). featureCounts: an efficient general purpose program for assigning sequence reads to genomic features. Bioinformatics *30*, 923–930. https://doi.org/10.1093/bioinformatics/btt656.

71. Love, M.I., Huber, W., and Anders, S. (2014). Moderated estimation of fold change and dispersion for RNA-seq data with DESeq2. Genome Biol. *15*, 550. https://doi.org/10.1186/s13059-014-0550-8.

72. Korotkevich, G., Sukhov, V., Budin, N., Sphak, B., Artyomov, M., and Sergushichev, A. (2021). Fast gene set enrichment analysis. Preprint at bioRxiv. https://www.biorxiv.org/content/10.1101/060012v3.

73. Dobin, A., Davis, C.A., Schlesinger, F., Drenkow, J., Zaleski, C., Jha, S., Batut, P., Chaisson, M., and Gingeras, T.R. (2013). STAR: ultrafast universal RNA-seq aligner. Bioinformatics *29*, 15–21. https://doi.org/10.1093/bioinformatics/bts635.

74. Jones, D., Raine, K.M., Davies, H., Tarpey, P.S., Butler, A.P., Teague, J.W., Nik-Zainal, S., and Campbell, P.J. (2016). cgpCaVEManWrapper: simple execution of CaVEMan in order to detect somatic single nucleotide variants in NGS data. Curr. Protoc. Bioinformatics *56*, 15.10.1–15.10.18. https://doi.org/10.1002/cpbi.20.

75. Raine, K.M., Hinton, J., Butler, A.P., Teague, J.W., Davies, H., Tarpey, P., Nik-Zainal, S., and Campbell, P.J. (2015). cgpPindel: identifying somatically acquired insertion and deletion events from paired end sequencing. Curr. Protoc. Bioinformatics *52*, 15.7.1–15.7.12. https://doi.org/10.1002/0471250953.bi1507s52.

76. Cruz, C., Castroviejo-Bermejo, M., Gutiérrez-Enríquez, S., Llop-Guevara, A., Ibrahim, Y.H., Gris-Oliver, A., Bonache, S., Morancho, B., Bruna, A., Rueda, O.M., et al. (2018). RAD51 foci as a functional biomarker of homologous recombination repair and PARP inhibitor resistance in germline BRCA-mutated breast cancer. Ann. Oncol. *29*, 1203–1210. https://doi.org/10.1093/annonc/mdy099.

77. Castroviejo-Bermejo, M., Cruz, C., Llop-Guevara, A., Gutiérrez-Enríquez, S., Ducy, M., Ibrahim, Y.H., Gris-Oliver, A., Pellegrino, B., Bruna, A., Guzmán, M., et al. (2018). A RAD 51 assay feasible in routine tumor samples calls PARP inhibitor response beyond BRCA mutation. EMBO Mol. Med. *10*, e9172. https://doi.org/10.15252/emmm.201809172.

78. Pellegrino, B., Herencia-Ropero, A., Llop-Guevara, A., Pedretti, F., Moles-Fernández, A., Viaplana, C., Villacampa, G., Guzmán, M., Rodríguez, O., Grueso, J., et al. (2022). Preclinical *in vivo* validation of the RAD51 test for identification of homologous recombination-deficient tumors and patient stratification. Cancer Res. *82*, 1646–1657. https://doi.org/10.1158/0008-5472.CAN-21-2409.

79. De Ronde, J., Klijn, C., and Velds, A. (2009). KCsmart: multi sample aCGH analysis package using kernel convolution. http://www.bioconductor.org/packages/release/bioc/html/KCsmart.html.

80. Anders, S., and Huber, W. (2010). Differential expression analysis for sequence count data. Genome Biol. *11*, R106. https://doi.org/10.1186/gb-2010-11-10-r106.

81. Hänzelmann, S., Castelo, R., and Guinney, J. (2013). GSVA: gene set variation analysis for microarray and RNA-Seq data. BMC Bioinf. *14*, 7. https://doi.org/10.1186/1471-2105-14-7.

STAR★METHODS

KEY RESOURCES TABLE

REAGENT or RESOURCE	SOURCE	IDENTIFIER
Antibodies		
Biotin Mouse Anti-Mouse H-2K (Clone SF1-1.1)	BD pharmingen	Cat# 553564; RRID: AB_394922
Biotin Mouse Anti-Mouse I-A (Clone AMS-32.1)	BD pharmingen	Cat# 553546; RRID: AB_394913
Anti-biotin Micro-Beads	Miltenyi Biotec	Cat# 130-090-485
Anti-human CD326 (EpCAM) eFluor 660 (Clone 1B7)	eBioscience	Cat# 50-9326-42; RRID: AB_10598658
Anti-human Ku80 (Clone C48E7)	Cell Signaling	Cat# 2180S
EnVision+ System- HRP Labeled Polymer Anti-Rabbit	Dako	Cat# K4003
CONFIRM anti-Estrogen Receptor (ER) (clone SP1)	Roche/Ventana	Cat# 5278406001
CONFIRM anti-Progesterone Receptor (PR) (clone 1E2)	Roche / Ventana	Cat# 5277990001
PATHWAY anti-HER-2/neu (clone 4B5)	Roche / Ventana	Cat# 5278368001
Ki-67 (clone MIB1)	Agilent / DAKO	Cat# M724001-2; RRID: AB_2631211
Recombinant Anti-Rad51 antibody (Clone EPR4030(3))	Abcam	Cat# ab133534; RRID: AB_2722613
NovocastraTM Liquid Mouse Monoclonal Antibody Geminin	Leica	Cat# NCL-L-Geminin; RRID: AB_563738
Geminin Polyclonal antibody	Proteintech	Cat# 10802-1-AP; RRID: AB_2110945
Anti-phospho-Histone H2A.X (Ser139) Antibody (clone JBW301)	Merck Millipore	Cat# 05-636; RRID: AB_309864
Goat anti-Rabbit IgG (H+L) Cross-Adsorbed Secondary Antibody, Alexa Fluor™ 568	Invitrogen	Cat# A-11011; RRID: AB_143157
Goat anti-Mouse IgG (H+L), Superclonal™ Recombinant Secondary Antibody, Alexa Fluor™ 488	Invitrogen	Cat# A28175; RRID: AB_2536161
Donkey anti-Mouse IgG (H+L) Highly Cross-Adsorbed Secondary Antibody, Alexa Fluor™ 568	Invitrogen	Cat# A10037; RRID: AB_2534013
Goat anti-Rabbit IgG (H+L) Cross-Adsorbed Secondary Antibody, Alexa Fluor™ 488	Invitrogen	Cat# A-11008; RRID: AB_143165
Anti-Actin, α-Smooth Muscle antibody, Mouse monoclonal (Clone 1A4)	Sigma Aldrich	Cat# A5228; RRID: AB_262054
CD324 (E-Cadherin) Monoclonal Antibody (DECMA-1), eBioscience™	eBioscience	Cat# 14-3249-82; RRID: AB_1210458
Donkey anti-Rabbit IgG (H+L) Highly Cross-Adsorbed Secondary Antibody, Alexa Fluor™ 568	Invitrogen	Cat# A10042; RRID: AB_2534017
Donkey anti-Rat IgG (H+L) Highly Cross-Adsorbed Secondary Antibody, Alexa Fluor™ 488	Invitrogen	Cat# A-21208; RRID: AB_2535794
Goat anti-Mouse IgG2a Cross-Adsorbed Secondary Antibody, Alexa Fluor™ 647	Invitrogen	Cat# A-21241; RRID: AB_2535810
Traztuzumab (Trazimera)	Pfizer	N/A
HER2/ErbB2 Rabbit mAb (Clone 29D8)	Cell Signaling	Cat# 2165; RRID: AB_10692490
Phospho-HER2/ErbB2 Antibody (Clone Tyr1248)	Cell Signaling	Cat# 2247; RRID: AB_331725

(*Continued on next page*)

Continued		
REAGENT or RESOURCE	SOURCE	IDENTIFIER
Phospho-Akt (Ser473) (D9E) XP® Rabbit mAb #4060	Cell Signaling	Cat# 4060; RRID: AB_2315049
Phospho-p44/42 MAPK (Erk1/2) (Thr202/Tyr204) (D13.14.4E) XP® Rabbit mAb #4370	Cell Signaling	Cat# 4370; RRID: AB_2315112
Biological samples		
Patient-derived xenografts (PDX)	The Netherlands Cancer Institute	This Paper
Chemicals, peptides, and recombinant proteins		
DAB	Sigma-Aldrich	Cat# D7304-1SET
DAB+	Dako	Cat# K3468
EZ Prep (10x)	Roche/Ventana	Cat# 5279771001
ULTRA Cell Conditioning (ULTRA CC1)	Roche/Ventana	Cat# 5424569001
ultra-View Universal DAB Detection Kit	Roche/Ventana	Cat# 5269806001
OptiView DAB IHC Detection Kit	Roche/Ventana	Cat# 6396500001
Bluing Reagent	Roche/Ventana	Cat# 5266769001
Antibody Diluent	Roche/Ventana	Cat# 5261899001
Target Retrieval Solution, pH 9 (10X)	Agilent/Dako	Cat# S236784-2
Wash Buffer 10x	Agilent/Dako	Cat# S300685-2
Antibody Diluent	Agilent/Dako	Cat# S080983-2
ProLong™ Gold Antifade Mountant with DAPI	Invitrogen	Cat# P36931
Hyaluronidase	Sigma Aldrich	Cat# H3884; CAS: 37326-33-3
Collagenase Type IV	Thermo Fischer	Cat# 17104019
Paraformaldehyde Aqueous Solution, EM Grade	Electron Microscopy Sciences	Cat# 50-980-487
DAPI	Sigma-Aldrich	Cat# D9542; CAS: 28718-90-3
VECTASHIELD® HardSet™ Antifade Mounting Medium	Vector Laboratories	Cat# H-1400-10
Deoxyribonuclease I from bovine pancreas	Sigma-Aldrich	Cat# DN25; CAS: 9003-98-9
17β-estradiol	Sigma-Aldrich	Cat# E2758; CAS: 50-28-2
FcR Blocking Reagent, human	Miltenyi Biotec	Cat# 130-059-901
17β-ESTRADIOL with biodegradable carrier-binder	Innovative Research of America	Cat# E−121
Collagenase A	Merck	Cat# 10103578001
DISCOVERY Anti-Rabbit HQ	Ventana	Cat# 760-4815
DISCOVERY Anti-HQ HRP	Ventana	Cat# 760-4820
DISCOVERY ChromoMap DAB Kit (RUO)	Ventana	Cat# 760-159
HistoChoice Clearing agent	Sigma Aldrich	Cat# H2779
Deposited data		
CNV-seq	EGA	EGAS00001006554
RNA-seq	EGA	EGAS00001006554
WES	EGA	EGAS00001006554
WGS	EGA	EGAS00001006554
Experimental models: Cell lines		
HEK-293T	ATCC	Cat# CRL-3216 RRID: CVCL_0063
Experimental models: Organisms/strains		
NOD.Cg-Prkdcscid Il2rgtm1Wjl/SzJ (NSG)	The Jackson Laboratory	Strain# 005557; RRID: IMSR_JAX:005557
Recombinant DNA		
Her2 (ERBB2) Human Tagged ORF Clone	Origene	Cat# RC222909
pRRL SFFV d20GFP.T2A.mTagBFP Donor	Addgene	Cat# 31485
Software and algorithms		
ImageJ	Schneider et al., 2012	https://imagej.nih.gov/ij/
LAS X 3D Visualization module	Leica microsystems	https://www.leica-microsystems.com/products/microscope-software/p/leica-las-af-3d-visualization/

(Continued on next page)

Continued

REAGENT or RESOURCE	SOURCE	IDENTIFIER
Cutadapt	Martin, 2011[61]	https://cutadapt.readthedocs.io/en/stable/
BWA aln	Li and Durbin, 2009[62]	http://bio-bwa.sourceforge.net/bwa.shtml
Disambiguate	Ahdesmäki et al., 2016[63]	https://github.com/AstraZeneca-NGS/disambiguate
Fastqc	Andrews, 2010[64]	https://www.bioinformatics.babraham.ac.uk/projects/fastqc/
Samtools	Li et al., 2009[65]	http://www.htslib.org/doc/samtools.html
Multiqc	Ewels et al., 2016[66]	https://multiqc.info/
QDNAseq	Scheinin et al., 2014[67]	https://bioconductor.org/packages/release/bioc/html/QDNAseq.html
Snakemake	Johannes & Rahmann, 2012[68]	https://snakemake.readthedocs.io/en/stable/
KCsmart	de Ronde, et al., 2022	https://bioconductor.org/packages/release/bioc/html/KCsmart.html
GISTIC2.0	Mermel et al, 2011[69]	https://www.genepattern.org/modules/docs/GISTIC_2.0
Mutect2	Benjamin et al, 2019	https://gatk.broadinstitute.org/hc/en-us/articles/360037593851-Mutect2
FeatureCounts	Liao et al., 2014[70]	https://academic.oup.com/bioinformatics/article/30/7/923/232889
DESeq2	Love et al, 2014[71]	https://bioconductor.org/packages/release/bioc/html/DESeq2.html
Fgsea	Korotkevich et al., 2019[72]	https://bioconductor.org/packages/release/bioc/html/fgsea.html
DIDS	de Ronde et al., 2013[26]	https://github.com/NKI-CCB/dids
STAR	Dobin et al., 2013[73]	https://github.com/alexdobin/STAR
Caveman	Jones et al., 2016[74]	https://github.com/cancerit/CaVEMan
Pindel	Raine et al., 2015[75]	https://github.com/cancerit/cgpPindel
Other		
LD columns	Miltenyi Biotec	Cat# 130-042-901

RESOURCE AVAILABILITY

Lead contact

Further information and requests for resources and reagents should be directed to and will be fulfilled by the lead contact, Professor Jos Jonkers (j.jonkers@nki.nl).

Materials availability

DCIS-MIND models generated in this study will be made available through CancerTools (https://www.cancertools.org/), Cancer Research UK's initiative to make research tools available from and to cancer researchers around the world.

Data and code availability

- RNAseq, CNVseq and Whole exome sequencing data generated in this paper are deposited in EGA under accession number (EGAS00001006554).
- This paper does not report original code.
- Any additional information required to re-analyze the data reported in this paper is available from the lead contact upon request.

EXPERIMENTAL MODEL AND SUBJECT DETAILS

Cell lines

Human embryonic kidney cell line HEK-293T was cultured in DMEM with 10% FBS and 1% penicillin/streptomycin at 37°C supplied with 5% CO_2.

Mice

As a mouse model we used 6-8 weeks old NOD-scid IL2Rgammanull (NSG) female mice obtained from Jackson Laboratories and bred in-house for experimental cohorts. All animal experiments were approved by the Animal Welfare Committee of The Netherlands Cancer Institute (NKI) in accordance with national guidelines. Animals were maintained in the animal department of the NKI, housed in individually ventilated cages (IVC) under specific pathogen-free (SPF) conditions, and received food and water *ad libitum*.

Human samples

Freshly resected DCIS material for establishment of PDX models was provided by the gross room (Antoni van Leeuwenhoek Hospital) and dissociated to single cells for intraductal implementation into immunocompromised mice. The study was approved by the institutional review board (NKI) and all subjects provided informed consent.

METHOD DETAILS

Patient derived DCIS xenograft models

Freshly resected DCIS material was provided by the gross room and mechanically dissociated. Next an overnight digestion at 37º C with continuous movement was performed containing 5 mg Collagenase Type IV (Thermo Fischer), 0,24 mg Hyaluronidase (Sigma), 200 mg BSA (Sigma) and 5 μl Gentamycin (Invitrogen) in 10 ml Advanced DMEM/F12 (Gibco) supplemented with 1% Penicillin-Streptomycin (Invitrogen), 1% L-Glutamine (Gibco) and 1mM HEPES (Sigma) per 100 mg of tumor tissue. Cells were further digested with 0.25% Trypsin-EDTA (Invitrogen) for 1 minute followed by incubation in 10U/μl DNAse (Sigma) in 4 ml Advanced DMEM/F12 while being vortexed for 3-5 min. Finally, the solution was filtered through a 70 μm cell strainer (BD Falcon) and counted before being intraductally injected into 6-8 weeks old NOD-scid IL2Rgammanull (NSG) female mice. Intraductal injections were performed under anesthesia (2,5% Isoflurane) using a 50 μl Hamilton syringe and a 34-gauge needle (Point style 4) to deliver cells into the mammary gland as previously described Behbod et al,[10] only without snipping the nipple and surgically opening the mouse. 20 μl of PBS (with 2ul of trypan blue) containing 25.000 cells were injected. Mice received 17β-estradiol (E2) supplementation either by slow-release pellets (0.18 mg, 90-day release pellets) or E2 supplementation (Sigma, E2758) in the drinking water (4 ug/ml). After 6- or 12- months mice were sacrificed and mammary tissues were fixed and embedded or taken on PBS for further processing.

Magnetic bead sorting and re-transplantation

In order to sequentially transplant DCIS-MIND models, intraductally injected mammary glands were excised at 12 months after intraductal injection and digested overnight as described before. The obtained single-cell solution is dissolved in 300 μl PBS with 0.5% BSA plus 100 μl of human Fcr blocking reagent (Miltenyi Biotec). Mouse cells were then magnetically labeled with mouse MHCI and MHCII antibodies (BD Parmingen) followed by MACS Anti-Biotin MicroBeads UltraPure (Miltenyi Biotec), and negatively sorted for human DCIS cells using LD columns (Milteny Biotec). A small part of the sorted cells was then stained with anti-human CD326 (EpCAM)-eFluor660 (eBioscience) and analyzed by fluorescence-activated cell sorting (FACS) for purity, while the rest of the cells was intraductally injected with 25.000 DCIS cells per mammary gland.

Estrogen sensitivity of ER^+ DCIS-MIND models

Female NSG mice were ovariectomized or sham operated at 6-8 weeks of age, one week before intraductal injection. Sham operated mice received E2 supplementation (4μg/ml, Sigma) in the drinking water starting 1 week before surgery until the end of experiment. Mice were monitored weekly and tumor volume was measured with a caliper; Volume was calculated using the formula: V= (width^2)*length/2. Mice were killed by CO2 asphyxiation, 6-12 months after intraductal injection or when cumulative tumor volume exceeded 2000 mm3. Mammary glands were fixed and embedded and if no palpable tumor was present, tumor area was measured from FFPE slides using a human specific anti-Ku80 antibody (Cell Signaling).

Herceptin (traztuzumab) treatment of mice

Mice, intraductally injected with $HER2^+$ DCIS (DCIS063 or DCIS088), were treated weekly with 10 mg/kg Herceptin (Trazimera, Pfizer in 0.9% NaCl) or vehicle one month after intraductal injection by intraperitoneal injection (I.P.). Mice were monitored weekly and tumor volume was measured with a caliper; Volume was calculated using the formula: V= (width^2)*length/2. Mice were sacrificed by CO2 asphyxiation, 12 months after intraductal injection or when cumulative tumor volume exceeded 2000 mm3.

Lentiviral expression in human DCIS of ERBB2 or Akaluciferase

Primary or PDX DCIS tissue was digested to a single-cell solution as described before and was transfected with a lentivirus (ERBB2-GFP lentivirus, Origene, RC222909L4, or Akaluciferase, courtesy of the lab of Shinae Kizaka-Kondo cloned into the pRRL vector backbone, Addgene plasmid #31485) or vehicle by putting the cells and the virus in a falcon tube and spin for 2 hours at 2500 rpm at RT. Transfected cells were then directly intraductally injected into 6-8 weeks old female NSG mice. 6 - 12 months after injection mice were sacrificed and mammary glands were fixed and embedded or processed for 3D whole-mount imaging. The ERBB2-GFP lentivirus functionality was tested by transfecting HEK-293T cells (ATCC) and performing a Western Blot with a HER2/ErbB2 and pHER2/ErbB2 antibody (Cell signaling) blocked in milk powder.

In vivo bioluminescence imaging

In vivo bioluminescence imaging was performed with Akalumine-HCl (Courtesy of Leiden university). Akalumine was dissolved at 2 mg/ml in sterile H2O and stored at −20°C. Akalumine-HCl solution was injected i.p. (0.01 mL/g body weight) and animals were anesthetized with 2–3% isoflurane. Light emission was measured 15 min after Akalumine administration by using a cooled CCD camera (IVIS; Xenogen), coupled to Living Image acquisition and analysis software (Living image 4.3 PerkinElmer) over an integration time of 1 min. Signal intensity was quantified as the Flux (photons per second) measured over the region of interest.

Immunohistochemistry (IHC)

Immunohistochemistry for ER, PR, HER2, p-ERK, p-AKT and Ki67 of the FFPE tumor samples was performed on a BenchMark Ultra autostainer (Ventana Medical Systems). Briefly, paraffin sections were cut at 3 μm, heated at 75ºC for 28 minutes and deparaffinized in the instrument with EZ prep solution (Ventana Medical Systems). Heat-induced antigen retrieval was carried out using Cell Conditioning 1 (CC1, Ventanta Medical Systems) for 36 minutes at 95ºC (ER, PR and HER2) for 32 minutes at 95°C (Phospho-Akt (Ser473)) or 64 minutes at 95ºC (Ki67, Phospho-p44/42 MAPK (ERK1/2) (Thr202/Tyr204)). ER was detected using clone SP1 (Ready-to-Use, 32 minutes at 36ºC, Roche Diagnostics), PR using clone 1E2 (Ready-to-Use, 32 minutes at 36ºC, Roche Diagnostics), HER2 using clone SP3 (1/100 dilution overnight at 4ºC, Thermo Fischer), P-Akt (Ser473) using Clone D9E (1/25 dilution, 1 hour at 37ºC, Cell Signaling), Phospho-p44/42 MAPK (ERK1/2) (Thr202/Tyr204) using Clone D13.14.4E (1/400 dilution, 1 hour at 37ºC, Cell Signaling) and ki67 using clone MIB1 (1/100 dilution, 1 hour at 37ºC, Agilent/DAKO). To reduce background signal for PR staining, after primary antibody incubation slides were incubated with normal antibody diluent (Roche Diagnostics) for 24 minutes. Bound ER and PR was detected using the UltraView Universal DAB Detection Kit (Ventana Medical Systems), while detection of Ki67 was visualized using the OptiView DAB Detection Kit (Ventana Medical Systems). HER2 was detected by labelled Polymer-HRP Anti-Rabbit Envision (30 min, Dako) and visualization by DAB (3-20 min, Sigma). P-AKT and p-ERK were detected using Anti-Rabbit HQ (Ventana Medical Systems) bound for 12 minutes, followed by binding with an enzyme conjugate Anti-HQ HRP (Ventana Medical Systems) for 12 - minutes. Bound antibody was visualized using a ChromoMap DAB Detection Kit (Ventana Medical Systems). Slides were counterstained with Haematoxylin and Bluing Reagent (Ventana Medical Systems). Immunohistochemistry for Ku80 of the FFPE tumor samples was performed manually. paraffin sections were cut at 3 μm, heated at 75ºC for 28 minutes and deparaffinized in an autostainer. Heat-induced antigen retrieval is carried out using Tris/EDTA pH 9.0 for 30 minutes in a water bath at 96ºC. After 30 minutes cooldown slides are rinsed three times with PBS/0.05% Tween 20. Inactivation If Endogenous Peroxidase is performed with 3% H2O2 in Methanol for 20 minutes, after which slides are rinsed three times with PBS/0.05% Tween 20. Slides are pre-incubated with PBS/4% BSA/5% NGS for 30 minutes before detection of human cells by anti-human Ku80, clone C48E7 (1:400, o/n at 4ºC, Cell Signaling). Bound Ku80 was detected by labelled Polymer-HRP Anti-Rabbit Envision (30 min, Dako) and visualization by DAB+ (3 min, Dako). Slides were counterstained with Haematoxylin.

RAD51 assay (immunofluorescence staining and scoring)

The following primary antibodies were used for immunofluorescence: rabbit anti-RAD51 (Abcam ab133534, 1:1000), mouse anti-geminin (NovoCastra NCL-L, 1:60), rabbit anti-geminin (ProteinTech 10802-1-AP, 1:400), mouse anti-y-H2AX (Millipore #05-636, 1:200). Goat anti-rabbit Alexa fluor 568 (Invitrogen; 1:500), goat anti-mouse Alexa fluor 488 (Invitrogen; 1:500), donkey anti-mouse Alexa fluor 568 (Invitrogen; 1:500), and goat anti-rabbit Alexa fluor 488 (Invitrogen; 1:500) were used as secondary antibodies. For target antigen retrieval, FFPE sections were microwaved for 20 min at 110°C in DAKO Antigen Retrieval Buffer pH 9.0. Sections were cooled down in distilled water for 5 min, then permeabilized with DAKO Wash Buffer (contains Tween-20) for 5 min, followed by incubation in blocking buffer (DAKO Wash Buffer with 1% bovine serum albumin) for 5 min. Primary antibodies were diluted in DAKO Antibody Diluent and incubated at room temperature for 1 h. Sections were washed for 5 min in DAKO Wash Buffer followed by 5 min in blocking buffer. Secondary antibodies were diluted in blocking buffer and incubated for 30 min at room temperature. The 2-step washing was repeated followed by 5-min incubation in distilled water. Dehydration was performed with increasing concentrations of ethanol (70%, 96% and 100%). Sections were mounted with DAPI ProLong Gold antifading reagent and stored at -20°C.

Biomarkers were quantified on FFPE PDX by scoring. Biomarker scoring was performed onto life images using a 60x-immersion oil lens. The RAD51 score represents the percentage of geminin$^+$ tumor cells with five or more RAD51 nuclear foci and the pre-defined cut-off of 10% was used to call homologous recombination (HR) proficient (HRP) and deficient (HRD). Samples with low yH2AX (<25% of geminin$^+$ cells with yH2AX foci) or with <40 geminin$^+$ cells were not included in the analyses, due to insufficient endogenous DNA damage or tumor cells in the S/G2- phase of the cell cycle, respectively.[76–78]

Whole-mount immunofluorescence staining of mammary glands

Intraductally injected mammary glands were dissected and incubated in a mixture of collagenase A (1 mg/ml, 10103586001, Merck) and hyaluronidase (50 μg/ml, H3506-1G, Merck) at 37ºC for optical clearance, fixed in periodate-lysine-paraformaldehyde (PLP) buffer (1% paraformaldehyde (PFA; Electron Microscopy Science), 0.01M sodium periodate, 0.075M L-lysine and 0.0375M P-Buffer (0.081M Na2HPO4 and 0.019M NaH2PO4;pH 7.4) for 2h at room temperature, and incubated for at least 3h in blocking buffer containing 1% bovine serum albumin (Roche Diagnostics), 5% normal goat serum (Monosan) and 0.8% Triton X-100 (Merck) in PBS. Primary antibodies were diluted in blocking buffer and incubated overnight at room temperature. Secondary antibodies diluted in blocking buffer were incubated for at least 6h. Nuclei were stained with DAPI (0.1 μg/ml; Sigma-Aldrich) in PBS. Glands were washed with PBS and mounted on a microscopy slide with Vectashield hard set (H-1400, Vector Laboratories). Primary

antibodies; anti-alpha smooth muscle actin (mouse monoclonal IgG2a, Sigma-Aldrich, A5228, 1:500), anti-E-cadherin (rat, eBioscience, 14-3249-82, 1:700), and anti-Ku80 (rabbit, Cell signaling, C48E7, 1:100). Secondary antibodies: donkey, anti-rabbit Alexa 568 (Invitrogen, A10042), donkey anti-rat Alexa 488 (Invitrogen, A21208), goat anti-mouse IgG2a Alexa 647 (Invitrogen, A21241), all 1:400.

Whole-mount imaging of mammary glands

Imaging of whole-mount mammary glands was performed using an inverted Leica TCS SP8 confocal microscope, equipped with a 405nm laser, an argon laser, a DPSS 561 nm laser and HeNe 633 nm laser. Different fluorophores were excited as followed: DAPI at 405 nm, Alexa 488 at 488 nm, Alexa 568 at 561 nm and Alexa 647 at 633 nm. DAPI was collected at 440-470 nm, Alexa 488 at 495-510 nm, Alexa 568 at 610-640 nm and Alexa 647 at 650-700 nm. All images were acquired with a 20x (HCX IRAPO N.A. 0.70 WD 0.5 mm) dry objective using a Z-step size of 5 μm (total Z-stack around 200 μm) for the whole gland overview scans, and Z-step size 0.5-1 μm for the detailed images. Three-dimensional overview tile scans of the mammary glands and detailed images of the individual clones were stitched and processed in the true 3D real-time rendering LAS X 3D Visualization module (Leica microsystems, Mannheim, Germany) All images were further processed using ImageJ software (https://imagej.nih.gov/ij/).

Immunofluorescent labeling of thick tissue sections

Intraductally injected mammary glands were dissected and fixed in 4% PFA (Electron Microscopy Science) overnight at 4°C. Fixed glands were washed in PBS and incubated in 30% sucrose for at least 8h at 4°C. Next, glands were embedded in cryomolds filled with cryoprotectant Optimal Cutting Temperature (OCT) (Tissue Tek). Thick sections of 50-100μm were cut along the sagittal plane of the mammary glands using a cryostat. Tissue sections were collected on adhesive glass slides (SuperFrost Ultra Plus, Fisher Scientific). Sections were washed in PBS for 5-10 minutes and incubated in blocking and permeabilization buffer containing 1% bovine serum albumin (Roche Diagnostics), 5% normal goat serum (Monosan) and 0.5% Triton X-100 (Merck) in PBS for 2h at room temperature. Primary antibodies were diluted in blocking/permeabilization buffer and incubated overnight at 4°C. Secondary antibodies diluted in blocking/permeabilization buffer were incubated for 2h at room temperature. Nuclei were stained with DAPI (0.1 μg/ml; Sigma-Aldrich) in PBS. Glands were washed with PBS and mounted on a microscopy slide with Vectashield hard set (H-1400, Vector Laboratories). Primary antibodies; anti-alpha smooth muscle actin (mouse monoclonal IgG2a, Sigma-Aldrich, A5228, 1:1000), anti-CD326 directly conjugated to Alexa Fluor 488 (EpCAM) (monoclonal, eBioscience, 1B7, 1;500), ECM1 (rabbit polyclonal, Thermo Fisher, BS-0776R, 1:200). Secondary antibodies; donkey, anti-rabbit Alexa 568 (Invitrogen, A10042), goat anti-mouse IgG2a Alexa 647 (Invitrogen, A21241), all 1:400.

3D imaging of human FFPE DCIS resections

FFPE blocks were tissue-cleared and stained with a modified FLASH protocol.[29]

Intact embedded tissue pieces were cut out of the histology cassettes with a razor blade, and deparaffinized in HistoChoice® for 2 hours at 54°C. The following steps were carried out at room temperature. Samples were washed 3 times in 100% MetOH, at least 1 hour each, followed by incubation in dichlormethane for 3 hours. Dichlormethane was refreshed for a second incubation overnight. Samples were washed twice in 100% MetOH for 1 hour each, and bleached in 15% DMSO, 15% H2O2 in MetOH. Bleaching solution was refreshed after 6 hours and kept overnight. The samples were then rehydrated through incubations in 75% and 30% MetOH in dH2O (1 hour each), followed by 2 washes in dH2O for 1 hour. The FLASH non-destructive antigen retrieval was carried out by incubating the samples in 200 mM boric acid, 4M urea and 8% 3-(Decyldimethylammonio)propanesulfonate inner salt (CAS 15163-36-7) in dH2O (pH ~7). Samples were incubated at RT for 1 hour, followed by overnight incubation at 37°C, after which the solution was refreshed and temperature increased to 54°C for 24 hours. Samples were washed in PBT (0,2% Triton X-100 in PBS) at least 3 times for 1 hour per wash at room temperature. Blocking for antibody labeling was carried out for 3 hours in blocking buffer (10% FBS, 1% BSA, 5% DMSO, 0,2% Triton X-100, 0,02% NaAzide in PBS). Samples were incubated with mouse αSMA antibody, clone 1A4 (Sigma) diluted 1:1000 in blocking buffer for 3 nights at room temperature. Samples were washed 4 times in PBS, 30 minutes per wash, and incubated in AlexaFluor™-568 conjugated secondary donkey anti mouse IgG (Invitrogen) antibody 1:1000 and 1:1000 Hoechst 33342 in blocking buffer for 3 nights. Samples were washed in PBS 4 times for 30 minutes each, and dehydrated through a gradient of 3-hour incubations in 30%, 50%, 75% and twice 100% methanol in dH2O. Samples were immersed in 30%, 70% and twice 100% methyl salicylate in methanol for 3-6 hours per incubation. After 2 days, the solvent was replaced with a 2:1 mixture of benzyl benzoate and benzyl alcohol. Samples were kept in the solvent until imaging.

Imaging was carried out on an inverted multiphoton confocal microscope (Leica TCS SP8 MP) with a 25X water immersion objective (Fluotar VISIR 25x/0.95). Tiled z-scans capturing the entire FFPE blocks were acquired in Resonant Mode (8-bit) with 512x512 or 256x256 pixel format, 8000 Hz scan speed, 1.25 zoom, 2x line average and 5-15 μm z-steps. Fluorophores were excited simultaneously with an Insight X3 tuneable two-photon laser at 800 nm. Three HyD-RLD detectors were used to simultaneously acquire SHG (390-410 nm), Hoechst emission (420-500 nm) and Alexa Fluor™ 568 emission (580-620 nm). Z-compensation of the detector gains was used to correct for lower detection levels in deeper tissue layers due to scattering of the emitted fluorescence. Imaris Viewer (9.7.2) was used for 3D visualization of the datasets and 3D reconstructions were performed in Aivia (10.5) through manual DCIS annotations in the pixel classifier module and 3D object generation from the annotated channel.

Pathology

All IHC and H&E slides were scored blindly by a pathologist in Slide Score (www.slidescore.com) (both from DCIS-MIND models and primary DCIS). ER, PR was scored on a scale from 0-100% (≥10% is positive), HER2 was scored as 0, 1, 2 or 3+ (3+ is positive), Ki67 was scored on a scale from 0-100%. Growth of human DCIS cells was assessed by Ku80 stainings as either negative or positive. Ku80 stainings and H&E stainings were scored blindly by a pathologist for grade, calcifications, periductal fibrosis, comedonecrosis, invasion and growth pattern. Further patient data was obtained from NKI patient records following institute guide lines.

Microdissection

The pathologist scored the tumor percentage and indicated the DCIS regions for isolation on a H&E slide.

From 5 to 15 (depending on the DCIS area size) FFPE 10 μm slides, the DCIS regions were dissected by scraping the areas off under a stereomicroscope using a needle. The scraped off tissue was stored in PKD digestion buffer (Qiagen, #80234) and stored at 4 ºC for up to a week. DNA and RNA was isolated simultaneously with the Allprep DNA/RNA FFPE isolation kit (Qiagen, #80234) by using the QIAcube, according to manufacturer's protocol.

CNA-seq

The total amount of DNA was quantified on the Nanodrop 2000 (Thermofisher). The amount of double stranded DNA in the genomic DNA samples was quantified by using the Qubit dsDNA HS Assay Kit (Invitrogen, cat no Q32851). A max of 2000 ng of double stranded genomic DNA were fragmented by Covaris shearing to obtain fragment sizes of 160-200 bp. Samples were purified using 2X Agencourt AMPure XP PCR Purification beads according to manufacturer's instructions (Beckman Coulter, cat no A63881). The sheared DNA samples were quantified and qualified on a BioAnalyzer system using the DNA7500 assay kit (Agilent Technologies cat no. 5067- 1506). With an input of maximum 1 μg sheared DNA, library preparation for Illumina sequencing was performed using the KAPA Hyper Prep Kit (KAPA Biosystems, KK8504). During library amplification 6-8 PCR cycles were used to obtain enough yield for the exome capture. After library preparation, the libraries were cleaned up using 1X AMPure XP beads. All DNA libraries are analyzed on a BioAnalyzer system using the DNA7500 chips for determining the molarity.

Up to 13 uniquely indexed samples are mixed together by equimolar pooling. The pools are analyzed on the Agilent Technologies 2100 Bioanalyzer. Pools are diluted to 10 nM, and measured on the qPCR. The pool is subjected to sequencing on an Illlumina HiSeq2500 machine, each pool in one lane of a single read 65 bp run, according to manufacturer's instructions. The resulting reads were trimmed using Cutadapt[61] to remove any remaining adapter sequences. The trimmed reads were aligned to the GRCh38 version 97 and GRCm38 version 89 reference genome using BWA aln.[62] Mouse reads were filtered out by AstraZeneca's tool disambiguate.[63] The resulting alignments were sorted and marked for duplicates using Picard tools. QC statistics from Fastqc,[64] Samtools[65] and the above-mentioned tools were collected and summarized using Multiqc.[66] The copy-number data was segmented using QDNAseq (version 1.22.0)[67] from Bioconductor. The entire analysis was implemented by Julian de Ruiter using Snakemake (snakemake version 7.2.1; wrapper version 0.60.0)[68] and is freely available on GitHub (https://github.com/jrderuiter/snakemake-cnvseq). Unsupervised clustering was performed on the segmented copy-number data. Copy-number instability was scored by calculating the fraction of bins with copy-number values above or below a threshold of respectively 2.5 and 1.5 in the segmented copy-number data. KCsmart R-package (version 2.48.0)[79] and GISTIC2.0[69] were used to determine focal copy number groupwise aberrations. For the oncoprint, genes were selected that were in the CGC-list (version August 2019; https://cancer.sanger.ac.uk/census).

Whole exome sequencing & panel sequencing

Genomescan prepared the samples according to the procedure for Hybridization Capture using an Agilent SureSelect custom 0.5-2.9Mb kit for the panelseq and the Agilent SureSelectXT Human All Exon V7 kit for the WES samples. The prepared libraries were sequenced with Illumina sequencing technology and prepared according to manufacturer's protocols. The reads were trimmed using Cutadapt[61] to remove any remaining adapter sequences, filtering reads shorter than 60 bp after trimming to ensure good mappability. The trimmed reads were aligned to the human (GRCh38) and mouse (GRCm38) reference genome using BWA. The human alignment was processed for duplicate marking, indel realignment, and base recalibration using Picard Tools and GATK, as recommended by GATK best practices, and filtered to remove contaminating mouse reads using AstraZeneca's tool disambiguate.[63] QC statistics from Fastqc.[64] FastQC: a quality control tool for high throughput sequence data. Available online at: http://www.bioinformatics.babraham.ac.uk/projects/fastqc) and the above-mentioned tools were collected and summarized using Multiqc.[66] Mutect2 was used for SNP calling followed by the LearnReadOrientationModel and FilterMutectCalls commands. SNPs that had a TLOD of <10, a coverage of less than 15, an alternative frequency of less than 0.2, had a different function than exonic or splicing, were classified as synonymous-SNV, and/or had a population frequency of more than 0.01 in one of the following databases downloaded with ANNOVAR (1) (1000g, Kaviar, hrcr1, gnomad_genome, gnomad_exome, esp6500siv2, exac_03, gme) were excluded. For the oncoprint, genes were selected that were in the PanelSeq-list (See supplemental informtion).

Whole genome sequencing

Whole genome sequencing was carried out by Illumina Cambridge Ltd, UK. Whole genome short insert 450-500bp libraries were prepared in accordance with Illumina protocols using either Illumina Truseq PCR free protocol or Truseq Nano kit with 5 PCR cycles, depending on the quantity of starting DNA available. 150bp paired-end sequencing was performed using a Hiseq X to achieve an

average sequence coverage of 113X in tumors and 38X in matched normal controls from the same individual. The resultant reads were aligned to the reference human genome (GRCh38) using a Burrows–Wheeler Aligner, bwa mem (version 0.7.17-r1188). Paired tumor-normal bam files were interrogated for somatic substitution mutations using Caveman (Cancer Variants through Expectation Maximisation) (1.13.15) https://github.com/cancerit/CaVEMan.[74] Small somatic insertions and deletions were called using split-read mapping using a modified Pindel version 3.2.0 https://github.com/cancerit/cgpPindel.[75] Annotation to Ensembl build 91 was used to identify mutations falling in coding regions of the genome. 5′UTR variants, silent, intronic, and upstream mutations were excluded. For the oncoprint, genes were selected that were in the PanelSeq-list (See Table S3).

RNA-seq

Quality and quantity of the total RNA from was assessed by the 2100 Bioanalyzer using a Nano chip (Agilent, Santa Clara, CA). The percentage of RNA fragments > 200nt fragment distribution values (DV200) were determined using the region analysis method according to the manufacturer's instructions manual (Illumina, technical-note-470-2014-001). Strand-specific libraries were generated using the TruSeq RNA Exome Library Prep Kit (Illumina Inc., San Diego) according to the manufacturer's instructions (Illumina, # 1000000039582v01). Briefly, total RNA was random primed and reverse transcribed using SuperScript II Reverse Transcriptase (Invitrogen, part # 18064-014) with the addition of Actinomycin D. Second strand synthesis was performed using Polymerase I and RNaseH with replacement of dTTP for dUTP. The generated cDNA fragments were 3′ end adenylated and ligated to Illumina Paired-end sequencing adapters and subsequently amplified by 15 cycles of PCR. The libraries were validated on a 2100 Bioanalyzer using a 7500 chip (Agilent, Santa Clara, CA) followed by a 1-4 plex library pooling containing up to 200ng of each sample.

The pooled libraries were enriched for target regions using the probe Coding Exome Oligos set (CEX, 45MB) according to the manufacturer's instruction (Illumina, # 1000000039582v01). Briefly, cDNA libraries and biotin- labeled capture probes were combined and hybridized using a denaturation step of 95°C for 10 minutes and an incubation step from 94°C to 58°C having a ramp of 18 cycles with 1 minute incubation and 2°C per cycle. The hybridized target regions were captured using streptavidin magnetic beads and subjected to two stringency washes, an elution step and a second round of enrichment followed by a cleanup using AMPure XP beads (Beckman, A63881) and PCR amplification of 10 cycles. The target enriched pools were analyzed on a 2100 Bioanalyzer using a 7500 chip (Agilent, Santa Clara, CA), diluted and subsequently pooled equimolar into a multi-plex sequencing pool. The libraries were sequenced with 65 base single reads on a HiSeq2500 using V4 chemistry (Illumina Inc., San Diego).

The reads were trimmed using Cutadapt[61] to remove any remaining adapter sequences, filtering reads shorter than 20 bp after trimming to ensure good mappability. The trimmed reads were aligned to the GRCh38 reference genome version 101 and GRCm38 reference genome version 100 using STAR.[73] Mouse reads were filtered out by AstraZeneca's tool disambiguate.[63] QC statistics from Fastqc[64] and the above-mentioned tools were collected and summarized using Multiqc.[66] Gene expression counts were generated by featureCounts[70] using gene definitions from Ensembl GRCh38 version 101. Normalized expression values were obtained by correcting for differences in sequencing depth between samples using DESeqs median-of-ratios approach[80] and then log-transforming the normalized counts. The entire analysis was implemented by Julian de Ruiter using Snakemake (snakemake version 7.2.1; wrapper version 0.63.0[68]; and is freely available on GitHub (https://github.com/jrderuiter/snakemake-rnaseq). DESeq2[71] version 1.30.1 was used for differential expression analysis followed by fgsea[72] version 1.16.0 for pathway analysis and DIDS[26] version 0.9.1 to identify subgroup markers in heterogeneous populations.

To analyze the 90 informative genes contributing to the three DCIS subtypes in the Translational Breast Cancer Research Consortium[15] we clustered the genes and the samples based on their normalized, log-transformed counts using hierarchical clustering algorithm (hclust function from the hclust R package). The distance measure was taken to be 1 - correlation (Pearson for genes and Spearman for samples) and the clustering method is ward.D2. The resulted heatmap with the dendograms was plotted using the ComplexHeatmap R package.

Single-sample gene set enrichment analysis (ssGSEA) has been done using GSVA package with the ssGSEA method and the default parameters.[81] The analysis has been done separately for the DCIS-MIND and primary DCIS samples (which were normalized separately in this case). After this we clustered the genes and the samples based on their normalized, log-transformed counts using hierarchical clustering algorithm (hclust function from the hclust R package). The distance measure was taken to be 1 - correlation (Pearson for genes and Spearman for samples) and the distance between DCIS-MIND and primary DCIS sample from the same patient were all set to zero, forcing them to be next to each other in the dendogram and the heatmap plot. The clustering method is ward.D2 and the resulted heatmap with the dendograms was plotted using the ComplexHeatmap R package.

RNAseq classifiers

To calculate the Oncotype DX DCIS score based on 12 genes[20] we used the following scheme: Normalization factor is obtained via raw counts of 5 reference genes: N = log2(ACTB + GAPDH + RPLP0 + GUSB + TFRC)/5. Then raw counts of genes are log2 transformed (with 0.5 pseudocount) and the normalization factor N is subtracted. Proliferation Group Score is calculated using the transformed counts: (MKI67 + AURKA + BIRC5 + CCNB1 + MYBL2)/5. DCIS score if finally obtained as 0.31*(Proliferation Group Score)-0.08*PGR - 0.09*GSTM1. Finally its normalized version is given by 66.7*(DCIS score)+10.

To calculate the Cox2, P16 and Ki67 classifier RNAseq data was used for COX2 and P16 with an expression level cut-off of 6 or 5 respectively for patient samples and 2 or 4 for PDX samples. For Ki67 IHC data was used, with a cut-off at 10%.

To calculate the 812-gene classifier, we performed the pipeline provided by Strand et al.[15]

CellPress
OPEN ACCESS

QUANTIFICATION AND STATISTICAL ANALYSIS

Linear regression analyses have been performed using IBM SPSS Statistics (IBM, Version 27). All quantifications were analyzed using a Student's t test, while survival curves were analyzed using a log-rank (Mantel-Cox) test performed in GraphPad Prism (GraphPad Software Inc, version 9). In all cases, the p values are represented as follows: ***$p < 0.001$, **$p < 0.01$, *$p < 0.05$, and not statistically significant when $p > 0.05$.

Cancer Cell, Volume *41*

Supplemental information

A living biobank of patient-derived ductal carcinoma *in situ* mouse-intraductal xenografts identifies risk factors for invasive progression

Stefan J. Hutten, Roebi de Bruijn, Catrin Lutz, Madelon Badoux, Timo Eijkman, Xue Chao, Marta Ciwinska, Michael Sheinman, Hendrik Messal, Andrea Herencia-Ropero, Petra Kristel, Lennart Mulder, Rens van der Waal, Joyce Sanders, Mathilde M. Almekinders, Alba Llop-Guevara, Helen R. Davies, Matthijs J. van Haren, Nathaniel I. Martin, Fariba Behbod, Serena Nik-Zainal, Violeta Serra, Jacco van Rheenen, Esther H. Lips, Lodewyk F.A. Wessels, Grand Challenge PRECISION Consortium, Jelle Wesseling, Colinda L.G.J. Scheele, and Jos Jonkers

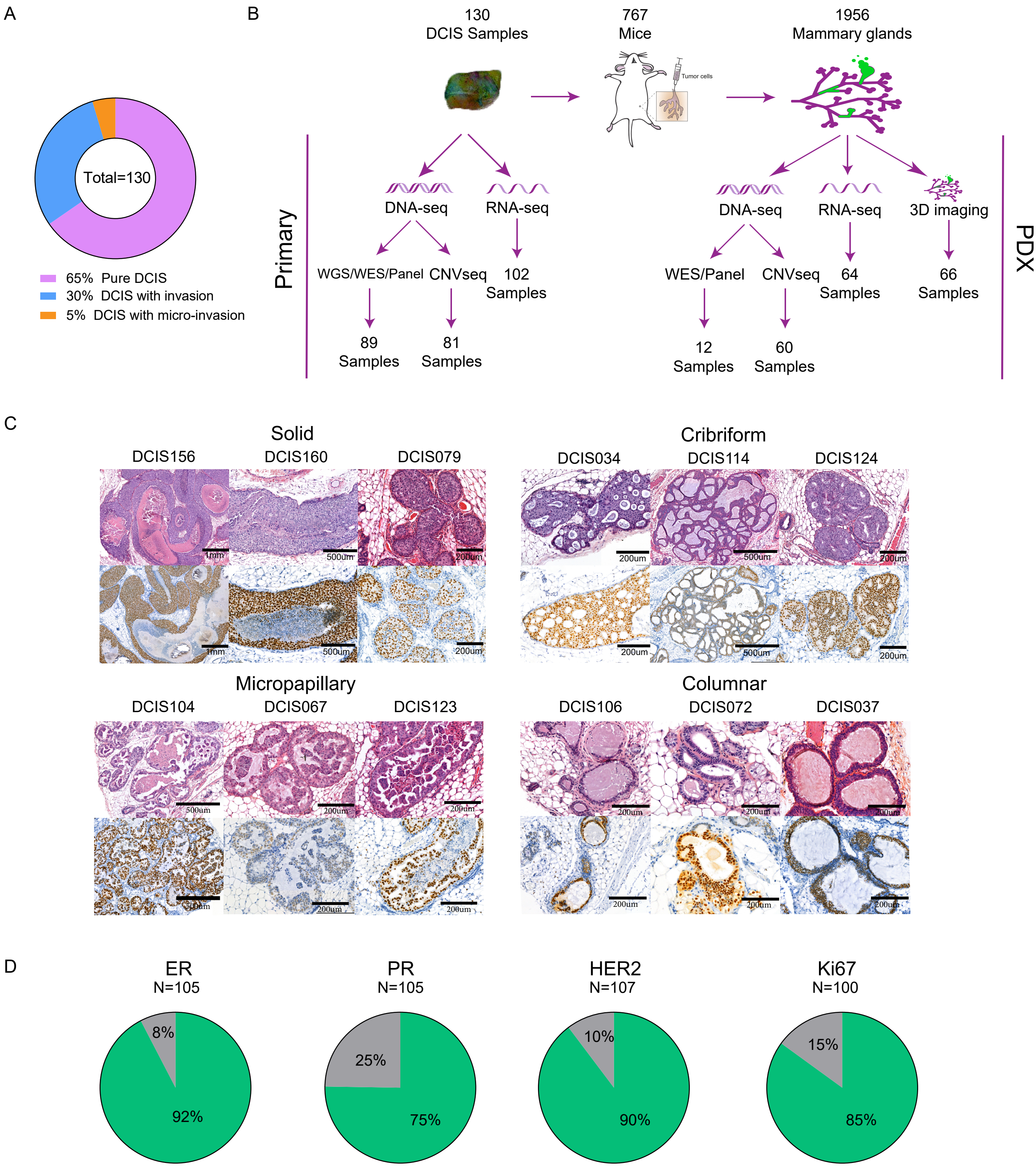

A
Total=130
65% Pure DCIS
30% DCIS with invasion
5% DCIS with micro-invasion
B
130 DCIS Samples
767 Mice
1956 Mammary glands
Tumor cells
Primary
DNA-seq
RNA-seq
WGS/WES/Panel
CNVseq
102 Samples
89 Samples
81 Samples
PDX
DNA-seq
RNA-seq
3D imaging
WES/Panel
CNVseq
64 Samples
66 Samples
12 Samples
60 Samples
C
Solid
DCIS156
DCIS160
DCIS079
Cribriform
DCIS034
DCIS114
DCIS124
Micropapillary
DCIS104
DCIS067
DCIS123
Columnar
DCIS106
DCIS072
DCIS037
D
ER
N=105
8%
92%
PR
N=105
25%
75%
HER2
N=107
10%
90%
Ki67
N=100
15%
85%

Figure S1. A comprehensive collection of 130 DCIS-MIND models, related to figure 1.

A) Pie chart of origin of DCIS with pure DCIS, DCIS with adjacent IBC or DCIS with micro-invasion. **B)** Schematic overview of the DCIS-MIND model collection and analyses performed on DCIS-MIND samples and primary DCIS lesions. **C)** Additional representative H&E (top) and human-specific Ku80 (bottom) images of DCIS-MIND models with a solid, cribriform, (micro-)papillary or columnar growth pattern. **D)** Pie charts of concordance between the primary DCIS lesion and matched DCIS-MIND lesion for ER, PR, HER2 and Ki67 respectively (green is concordant, grey is discordant). ER, PR and HER2 is concordant when both cases are positive (≥10% cells positive) or negative (≤10% cells positive), Ki67 is concordant when the percentage of positive cells is within 10% difference.

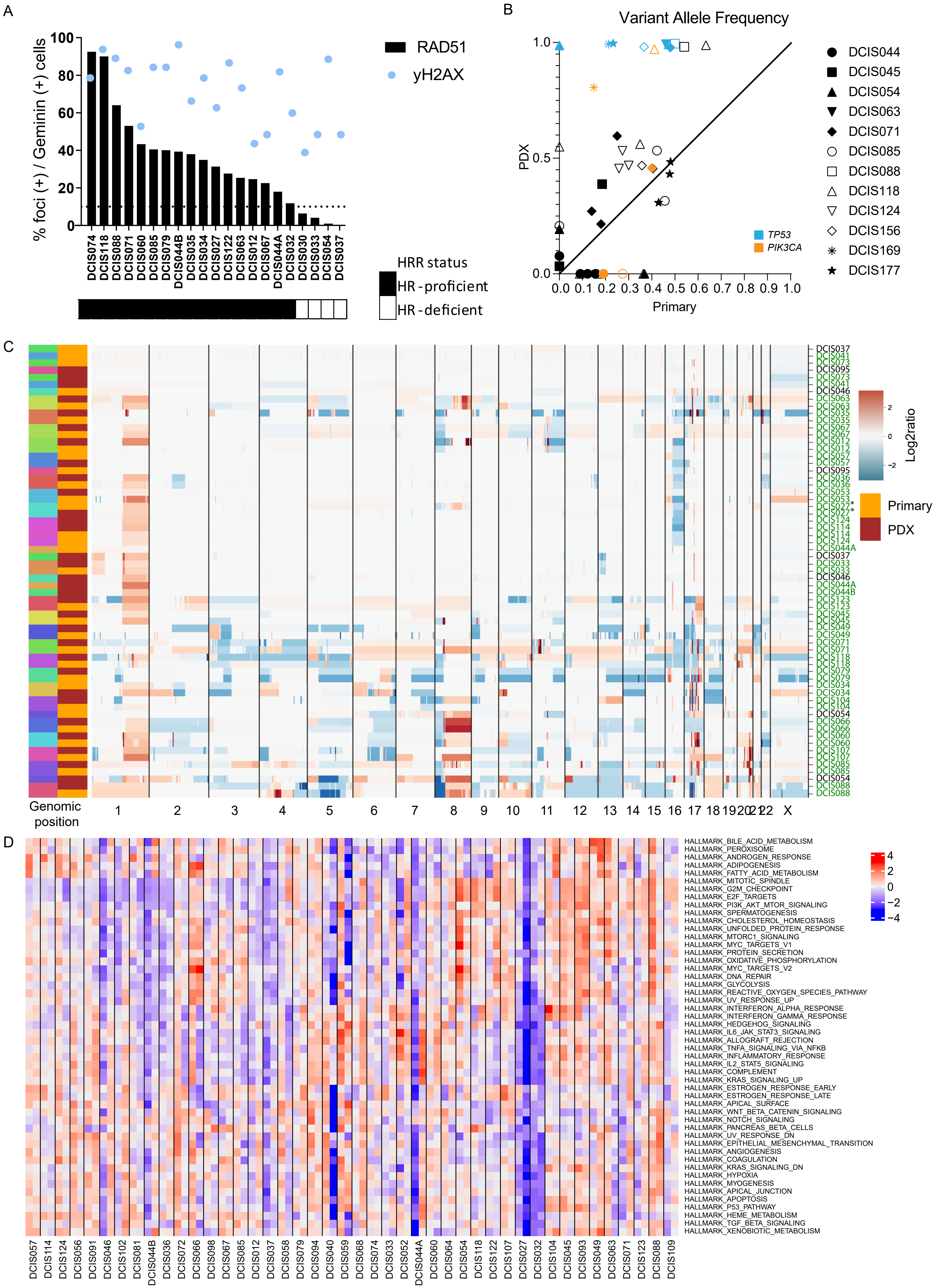

A
100
80
60
40
20
0
% foci (+) / Geminin (+) cells
RAD51
yH2AX
DCIS074
DCIS118
DCIS088
DCIS071
DCIS060
DCIS085
DCIS079
DCIS044B
DCIS035
DCIS034
DCIS027
DCIS122
DCIS063
DCIS012
DCIS067
DCIS044A
DCIS032
DCIS030
DCIS033
DCIS054
DCIS037
HRR status
HR-proficient
HR-deficient
B
Variant Allele Frequency
1.0
0.5
0.0
PDX
0.0 0.1 0.2 0.3 0.4 0.5 0.6 0.7 0.8 0.9 1.0
Primary
TP53
PIK3CA
DCIS044
DCIS045
DCIS054
DCIS063
DCIS071
DCIS085
DCIS088
DCIS118
DCIS124
DCIS156
DCIS169
DCIS177
C
Log2ratio
2
0
-2
Primary
PDX
Genomic position
1 2 3 4 5 6 7 8 9 10 11 12 13 14 15 16 17 18 19 20 21 22 X
D
4
2
0
-2
-4
HALLMARK_BILE_ACID_METABOLISM
HALLMARK_PEROXISOME
HALLMARK_ANDROGEN_RESPONSE
HALLMARK_ADIPOGENESIS
HALLMARK_FATTY_ACID_METABOLISM
HALLMARK_MITOTIC_SPINDLE
HALLMARK_G2M_CHECKPOINT
HALLMARK_E2F_TARGETS
HALLMARK_PI3K_AKT_MTOR_SIGNALING
HALLMARK_SPERMATOGENESIS
HALLMARK_CHOLESTEROL_HOMEOSTASIS
HALLMARK_UNFOLDED_PROTEIN_RESPONSE
HALLMARK_MTORC1_SIGNALING
HALLMARK_MYC_TARGETS_V1
HALLMARK_PROTEIN_SECRETION
HALLMARK_OXIDATIVE_PHOSPHORYLATION
HALLMARK_MYC_TARGETS_V2
HALLMARK_DNA_REPAIR
HALLMARK_GLYCOLYSIS
HALLMARK_REACTIVE_OXYGEN_SPECIES_PATHWAY
HALLMARK_UV_RESPONSE_UP
HALLMARK_INTERFERON_ALPHA_RESPONSE
HALLMARK_INTERFERON_GAMMA_RESPONSE
HALLMARK_HEDGEHOG_SIGNALING
HALLMARK_IL6_JAK_STAT3_SIGNALING
HALLMARK_ALLOGRAFT_REJECTION
HALLMARK_TNFA_SIGNALING_VIA_NFKB
HALLMARK_INFLAMMATORY_RESPONSE
HALLMARK_IL2_STAT5_SIGNALING
HALLMARK_COMPLEMENT
HALLMARK_KRAS_SIGNALING_UP
HALLMARK_ESTROGEN_RESPONSE_EARLY
HALLMARK_ESTROGEN_RESPONSE_LATE
HALLMARK_APICAL_SURFACE
HALLMARK_WNT_BETA_CATENIN_SIGNALING
HALLMARK_NOTCH_SIGNALING
HALLMARK_PANCREAS_BETA_CELLS
HALLMARK_UV_RESPONSE_DN
HALLMARK_EPITHELIAL_MESENCHYMAL_TRANSITION
HALLMARK_ANGIOGENESIS
HALLMARK_COAGULATION
HALLMARK_KRAS_SIGNALING_DN
HALLMARK_HYPOXIA
HALLMARK_MYOGENESIS
HALLMARK_APICAL_JUNCTION
HALLMARK_APOPTOSIS
HALLMARK_P53_PATHWAY
HALLMARK_HEME_METABOLISM
HALLMARK_TGF_BETA_SIGNALING
HALLMARK_XENOBIOTIC_METABOLISM
DCIS057
DCIS114
DCIS124
DCIS056
DCIS091
DCIS046
DCIS102
DCIS081
DCIS044B
DCIS036
DCIS072
DCIS066
DCIS098
DCIS067
DCIS085
DCIS012
DCIS037
DCIS058
DCIS079
DCIS094
DCIS040
DCIS059
DCIS068
DCIS074
DCIS033
DCIS052
DCIS044A
DCIS060
DCIS064
DCIS054
DCIS118
DCIS122
DCIS107
DCIS027
DCIS032
DCIS104
DCIS045
DCIS093
DCIS049
DCIS063
DCIS071
DCIS123
DCIS088
DCIS109

Figure S2. RAD51 assay and RNA expression profile correlation, related to figure 2

A) RAD51 score of a subset of DCIS-MIND models. Models are homologous recombination proficient (HR- proficient), when ≥10% of cells have ≥5 RAD51 foci. **B)** Variant allele frequency (VAF) analyses comparing matched primary DCIS and DCIS-MIND samples, TP53 mutations are indicated in blue and PIK3CA mutations in orange. **C)** Unsupervised clustering of primary DCIS lesions and corresponding DCIS-MIND lesions, based on Copy number aberration (CNA), where blue indicates losses and red indicates gains. Matched primary/MIND models are color-coded and green model annotation indicates a high concordance in CNA. **D)** ssGSEA analyses performed on matched primary DCIS and DCIS-MIND samples and clustered together to show retained expression.

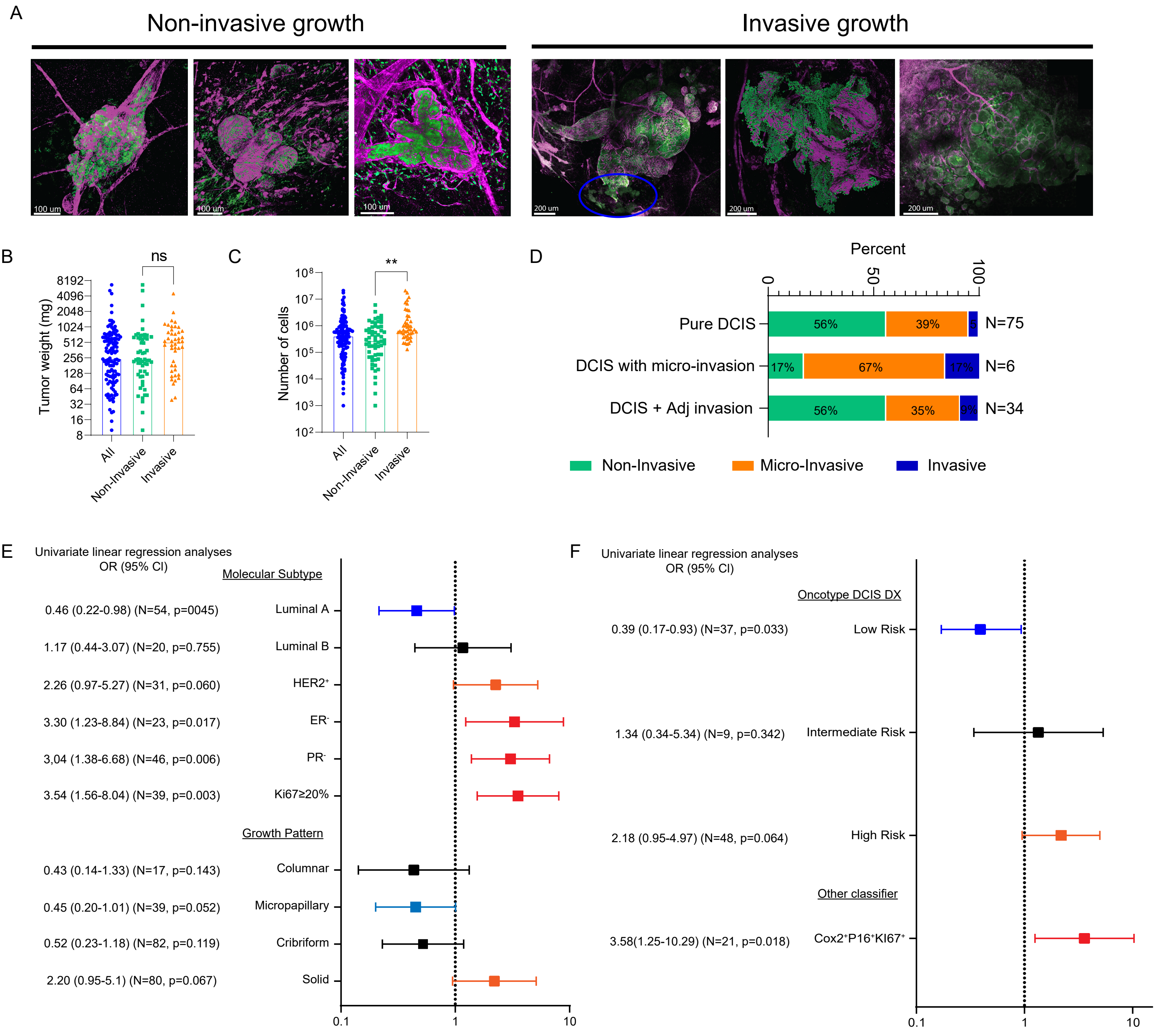
A
Non-invasive growth
Invasive growth
100 um
200 um
B
ns
Tumor weight (mg)
8192
4096
2048
1024
512
256
128
64
32
16
8
All
Non-Invasive
Invasive
C
**
Number of cells
10^8
10^7
10^6
10^5
10^4
10^3
10^2
D
Percent
0
50
100
Pure DCIS
56%
39%
N=75
DCIS with micro-invasion
17%
67%
17%
N=6
DCIS + Adj invasion
56%
35%
N=34
Non-Invasive
Micro-Invasive
Invasive
E
Univariate linear regression analyses
OR (95% CI)
Molecular Subtype
0.46 (0.22-0.98) (N=54, p=0045)
Luminal A
1.17 (0.44-3.07) (N=20, p=0.755)
Luminal B
2.26 (0.97-5.27) (N=31, p=0.060)
HER2+
3.30 (1.23-8.84) (N=23, p=0.017)
ER-
3,04 (1.38-6.68) (N=46, p=0.006)
PR-
3.54 (1.56-8.04) (N=39, p=0.003)
Ki67≥20%
Growth Pattern
0.43 (0.14-1.33) (N=17, p=0.143)
Columnar
0.45 (0.20-1.01) (N=39, p=0.052)
Micropapillary
0.52 (0.23-1.18) (N=82, p=0.119)
Cribriform
2.20 (0.95-5.1) (N=80, p=0.067)
Solid
0.1
1
10
F
Univariate linear regression analyses
OR (95% CI)
Oncotype DCIS DX
0.39 (0.17-0.93) (N=37, p=0.033)
Low Risk
1.34 (0.34-5.34) (N=9, p=0.342)
Intermediate Risk
2.18 (0.95-4.97) (N=48, p=0.064)
High Risk
Other classifier
3.58(1.25-10.29) (N=21, p=0.018)
Cox2+P16+KI67+
0.1
1
10

Figure S3. Whole-mount images of non-invasive vs invasive growth and additional parameters related to invasive progression, related to figure 3

A) Additional whole-mount images of non-invasive growth (left) and invasive growth (right). Cells of human origin are marked with Ku80 (green), myoepithelial cells are marked with αSMA (magenta). Blue circle indicates invasive cells. **B)** Boxplot of tumor weight of DCIS samples received, divided by all, non-invasive and invasive DCIS-MIND models. Data are represented as mean ± SEM. Unpaired two-tailed Student's t test. ns, not significant. **C)** Bar plot of number of cells obtained after processing of DCIS samples received, divided by all, non-invasive and invasive DCIS-MIND models. Data are represented as mean ± SEM. Unpaired two-tailed Student's t test. $**p < 0.01$. **D**) Diagram showing the distribution of non-invasive, micro-invasive and invasive progression of DCIS-MIND models, comparing models originating from pure DCIS, DCIS with micro-invasion and DCIS with adjacent invasion **E)** Odds ratio table showing univariate risk scores for association between molecular subtype and growth pattern parameters and invasive progression of DCIS in MIND-PDX models based on patient characteristics. **F)** Odds ratio table showing univariate risk scores for the Oncotype DX DCIS- and $COX2^{+}P16^{+}Ki67^{+}$ classifier based on primary data.

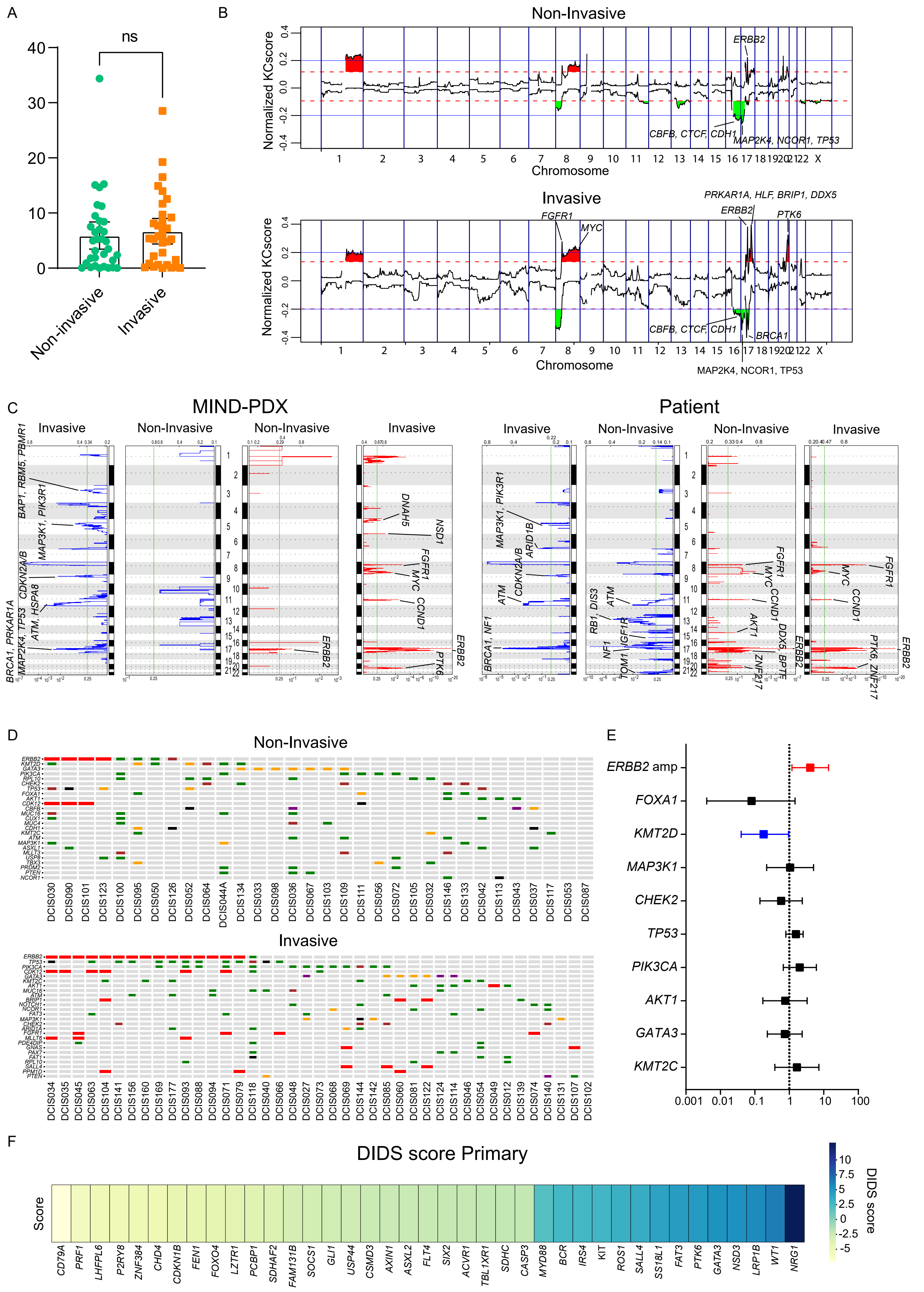

A
ns
40
30
20
10
0
Non-invasive
Invasive
B
Non-Invasive
Normalized KCscore
Chromosome
ERBB2
CBFB, CTCF, CDH1
MAP2K4, NCOR1, TP53
Invasive
FGFR1
MYC
PRKAR1A, HLF, BRIP1, DDX5
ERBB2
PTK6
BRCA1
MAP2K4, NCOR1, TP53
C
MIND-PDX
Patient
Invasive
Non-Invasive
Non-Invasive
Invasive
Invasive
Non-Invasive
Non-Invasive
Invasive
D
Non-Invasive
Invasive
E
ERBB2 amp
FOXA1
KMT2D
MAP3K1
CHEK2
TP53
PIK3CA
AKT1
GATA3
KMT2C
0.001
0.01
0.1
1
10
100
F
DIDS score Primary
Score
DIDS score
CD79A
PRF1
LHFPL6
P2RY8
ZNF384
CHD4
CDKN1B
FEN1
FOXO4
LZTR1
PCBP1
SDHAF2
FAM131B
SOCS1
GLI1
USP44
CSMD3
AXIN1
ASXL2
FLT4
SIX2
ACVR1
TBL1XR1
SDHC
CASP3
MYD88
BCR
IRS4
KIT
ROS1
SALL4
SS18L1
FAT3
PTK6
GATA3
NSD3
LRP1B
WT1
NRG1

Figure S4. Comprehensive molecular analyses of primary DCIS for low-risk DCIS vs high-risk DCIS and GISTIC2, related to figure 4

A) Scatter plot of percentage of genome with copy number alterations in matched primary DCIS lesions of DCIS-MIND models. Data are represented as mean ± SEM. Unpaired two-tailed Student's t test. ns, not significant. **B)** KCSmart analysis of CNV-seq data of primary DCIS from non-invasive DCIS models (top panel) and invasive DCIS models (lower panel), showing recurrent DNA copy number losses and gains. Non-invasive DCIS models show recurrent 1q gains and 16q losses, whereas invasive DCIS models show recurrent amplifications of *FGFR1*, *MYC*, *CCND1*, *ERBB2* and *PTK6* as well as losses of *MAP3K1* and *CDKN2A/B*. Genes from our breast cancer gene panel were annotated. **C)** GISTIC2 analysis of CNV-seq data of DCIS-MIND models (left panels) and primary DCIS lesions (right panels) from non-invasive DCIS models and invasive DCIS models. Blue: deletion; Red: Gain, Genes from our breast cancer gene panel were annotated. **D)** Oncoprint showing the mutational landscape of the primary DCIS lesions corresponding to the non-invasive (Top) and invasive (Bottom) DCIS-MIND models, respectively, including amplifications, single-nucleotide variants and insertion-deletions (indels) for the top mutated genes. **E)** Odds ratio table showing risk scores for commonly mutated genes, indicating ERBB2 amplifications and KMT2D mutations as significant odds ratio scores. **F)** Detection of Imbalanced Differential Signal (DIDS) scores for differentially expressed genes between primary DCIS lesions from non-invasive and invasive DCIS models.

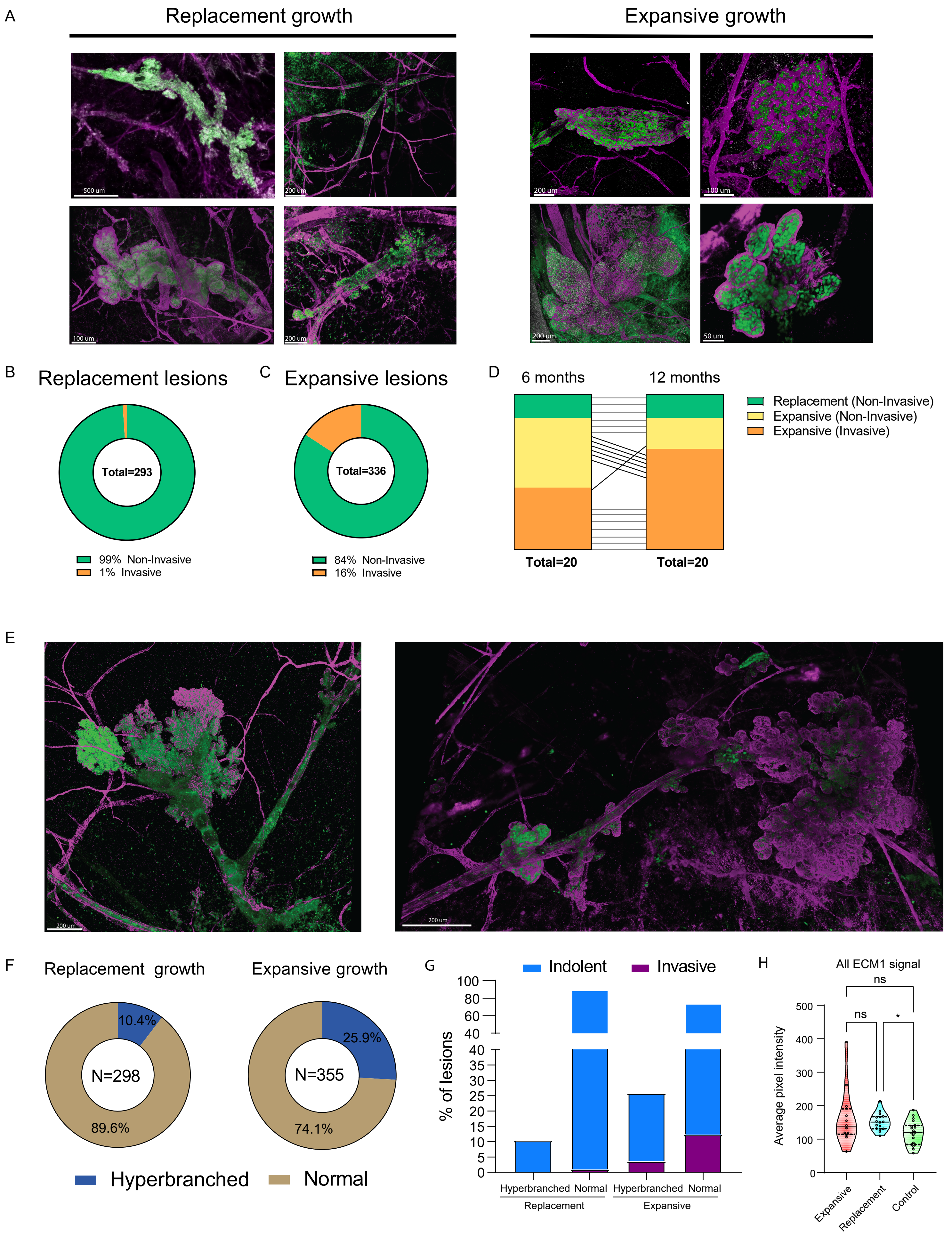

A
Replacement growth
Expansive growth
500 um
200 um
100 um
200 um
200 um
100 um
200 um
50 um
B
Replacement lesions
Total=293
99% Non-Invasive
1% Invasive
C
Expansive lesions
Total=336
84% Non-Invasive
16% Invasive
D
6 months
12 months
Total=20
Total=20
Replacement (Non-Invasive)
Expansive (Non-Invasive)
Expansive (Invasive)
E
200 um
200 um
F
Replacement growth
Expansive growth
10.4%
N=298
89.6%
25.9%
N=355
74.1%
Hyperbranched
Normal
G
Indolent
Invasive
% of lesions
Hyperbranched
Normal
Hyperbranched
Normal
Replacement
Expansive
H
All ECM1 signal
ns
ns
*
Average pixel intensity
Expansive
Replacement
Control

Figure S5. 3D growth pattern analyses and hyperbranching, related to figure 5

A) Examples of 3D whole-mount imaging of DCIS-MIND lesions, showing replacement growth (left panel) and expansive growth (right panel). Cells of human origin are marked with Ku80 (green), myoepithelial cells are marked with αSMA (magenta). **B)** Percentage of DCIS lesions showing invasion with replacement growth. **C)** Percentage of DCIS lesions showing invasion with expansive growth. **D)** Comparison of 3D growth pattern and invasion at an early- and late time point, 6 months and 12 months respectively. Grey line indicates concordance between early- and late time point, whereas a black line indicates a discordance between early-and late time point. **E)** Representative confocal whole-mount images of hyperbranching occurring around DCIS lesions. Cells of human origin are marked with Ku80 (green), myoepithelial cells are marked with αSMA (magenta) **F)** Percentage of replacement (left) and expansive (right) lesions with hyperbranching. **G)** Bar chart of percentage of DCIS lesions with invasive progression per 3D growth pattern, with or without hyperbranching. **H)** Quantification of total ECM1 expression in DCIS lesions for expansive or replacement growth, and normal mammary ducts. Lines indicate Q^1, Median and Q^3. Two-tailed Mann-Whitney test. $* \ p < 0.05$, ns, not significant.

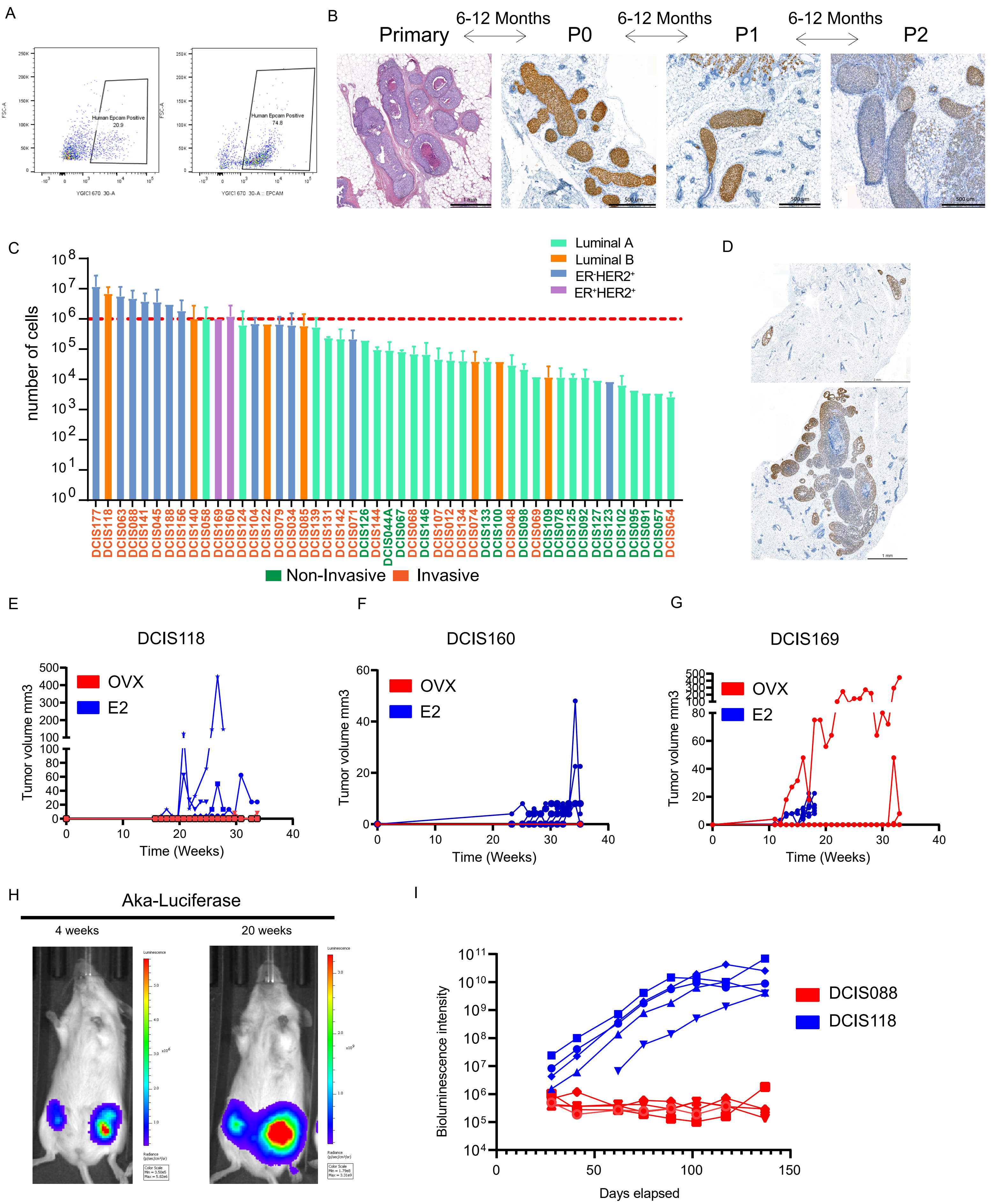

A
B
Primary
6-12 Months
P0
6-12 Months
P1
6-12 Months
P2
C
number of cells
Luminal A
Luminal B
ER-HER2+
ER+HER2+
Non-Invasive
Invasive
D
E
DCIS118
OVX
E2
Tumor volume mm3
Time (Weeks)
F
DCIS160
G
DCIS169
H
Aka-Luciferase
4 weeks
20 weeks
I
Bioluminescence intensity
Days elapsed
DCIS088
DCIS118

Figure S6. Sequential transplantation of DCIS and applications, related to figure S6

A) Representative FACS plot of human epithelial cells in a passaged sample marked with EpCAM, before (left) and after (right) magnetic bead sorting. **B)** Representative images of the growth pattern of DCIS118 as primary lesion (H&E), P0 (Ku80), P1 (Ku80) and P2 (Ku80). **C)** Distribution of the average number of tumor cells harvested from DCIS passages. Nineteen transplantable DCIS models yield more than 1,000,000 cells per passage and are therefore suitable for distribution to other labs. Data are represented as mean ± SEM. **D)** Representative images of DCIS085 outgrowth (Ku80) in ovariectomized (OVX) mice (top) and in E2 supplemented mice (bottom) **E-G)** Growth curves of DCIS118 **(E)**, DCIS160 **(F)** and DCIS169 **(G)** outgrowth in OVX mice and in E2-supplemented mice (E2 supplemented mice of DCIS169 had to be sacrificed at 18 weeks due to an infection). **H)** Representative images of bioluminescence of DCIS118 with Akaluciferase at 4 weeks (left) and 20 weeks (right) after injection. **I)** Bioluminescence intensity curves of DCIS118-Akaluciferase and DCIS088-Akaluciferase.

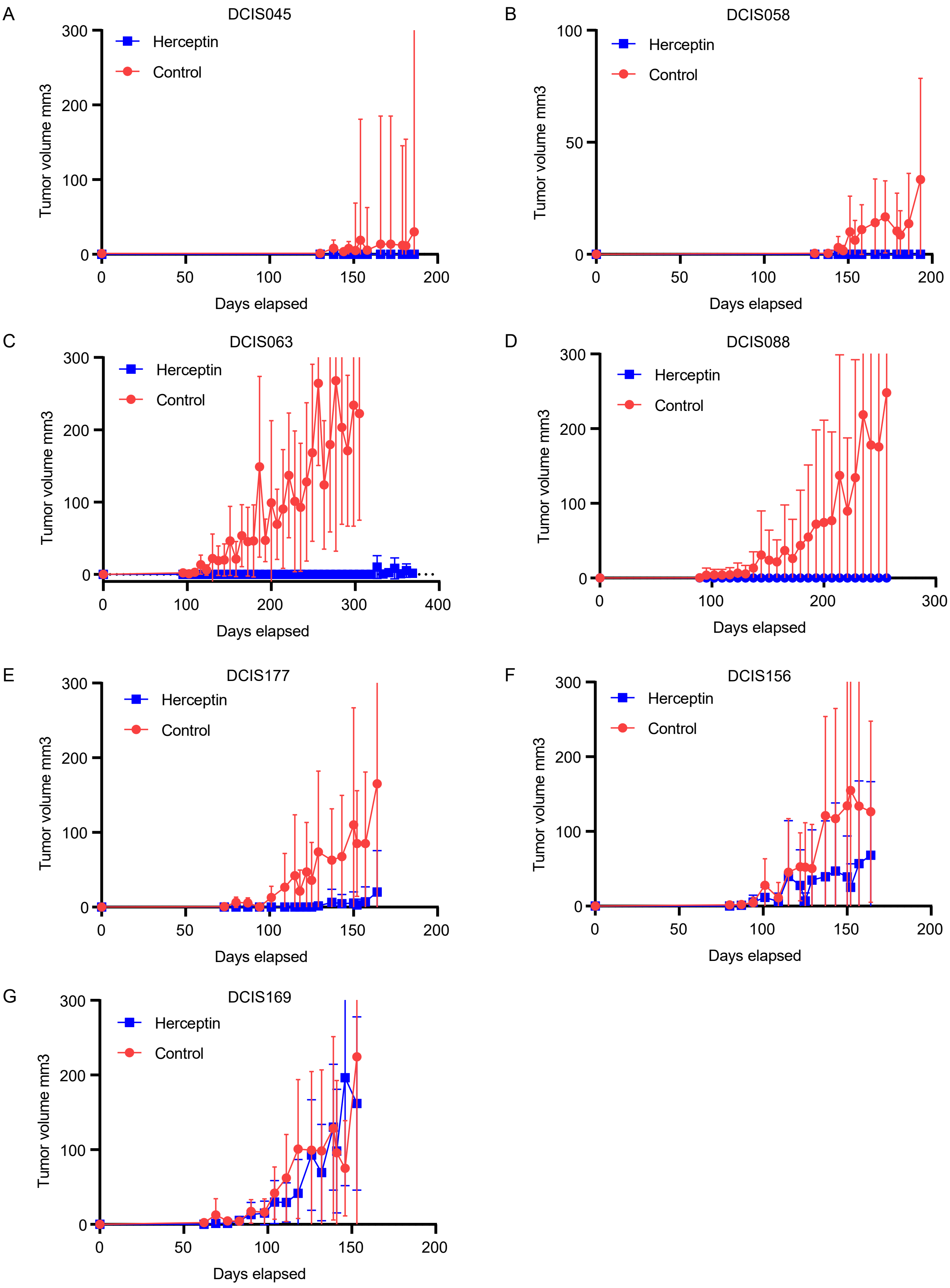

Figure S7. Altering HER2 expression changes growth kinetics of DCIS, related to figure S7

A-G) Growth curves of DCIS045 **(A)**, DCIS058 **(B)**, DCIS063 **(C)**, DCIS088 **(D)**, DCIS177 **(E)**, DCIS156 **(F)**, DCIS169 **(G)** with Herceptin treatment (10 mg/kg) or vehicle.
